# Gold(I)-catalyzed synthesis of γ-vinylbutyrolactones by intramolecular oxaallylic alkylation with alcohols

**DOI:** 10.3762/bjoc.7.139

**Published:** 2011-09-01

**Authors:** Michel Chiarucci, Mirko Locritani, Gianpiero Cera, Marco Bandini

**Affiliations:** 1 Dipartimento di Chimica “G. Ciamician”, Alma Mater Studiorum – Università di Bologna, Via Selmi 2, 40126 Bologna, Italy

**Keywords:** alcohol, butyrolactone, carbene, gold-catalysis, intramolecular oxaallylic alkylation

## Abstract

Gold(I)-*N*-heterocyclic carbene (NHC) complexes proved to be a reliable catalytic system for the direct synthesis of functionalized γ-vinylbutyrolactones by intramolecular oxaallylic alkylation with primary alcohols. Good isolated chemical yields were obtained for a range of malonyl and acetate derivatives. The good performance in reagent-grade solvents and the functional group/moisture tolerance make this catalytic process a promising route for the synthesis of architecturally complex polycyclic structures.

## Introduction

Allylic alcohols are highly desirable, readily available, cheap, and environmental sustainable reaction partners for allylic alkylation reactions in the presence of C- as well as X-based (X: heteroatom) nucleophiles [1–2]. Despite their undoubted synthetic/economic advantages (i.e., water is the only stoichiometric byproduct produced), the intrinsic lower reactivity of allylic alcohols compared to allyl halides/acetates/carbonates generally necessitates harsher reaction conditions and/or the need for activating agents (i.e., Brønsted or Lewis acids) [3–4].

Recently, late-transition metal (LTM) catalysis (i.e., Hg, Pd, Pt, Au, and Ru) has received growing attention in organic synthesis and enables unprecedented manipulations of unfunctionalized hydrocarbons under mild reaction conditions [5–9]. In this context, electrophilic LTM activation of carbon–carbon unsaturations, adjacent to alcoholic moieties (i.e., allylic, benzylic, and propargylic alcohols, usually referred to as π-activated alcohols), deserves a particular mention [10–13].

As a part of our ongoing interest in the gold-catalyzed allylic functionalization of C- and heteroatom-based nucleophiles with alcohols [14–17], we previously observed the formation of synthetically useful vinylbutyrolactones [18–22] as minor products in the Friedel–Crafts-type allylic alkylation of arenes [23]. The wide impact of functionalized γ-lactones on the synthesis of naturally occurring compounds [24–26] prompted us to optimize a direct synthesis of vinylbutyrolactones by direct gold activation of allylic alcohols [27–31] with esters [32–37].

**Figure 1:**
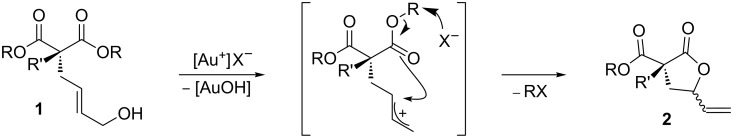
Working hypothesis for the present gold-catalyzed oxaallylic alkylation reaction.

In this direction, we targeted malonyl alcohols **1** as a readily available class of model acyclic precursors to create chemical diversity through an oxaallylic ring-closing reaction (Figure 1).

It should be noted that the synthesis of such a class of heterocyclic compounds has been the subject of several investigations. Among them, a multi-step synthetic pathway with final TBAF-promoted cyclization was proposed by Lepore [38] and, almost simultaneously, Poli and Prestat described a Pd-catalyzed Tsuji–Trost-type allylic alkylation procedure to obtain valuable precursors (i.e., lactams and lactones) of podophyllotoxin analogs [39–40]. However, to the best of our knowledge, no examples of metal-catalyzed lactonization through direct activation of allylic alcohols have been described so far.

## Results and Discussion

At the outset of our investigation, we focused our attention on the non-enolizable allyl alcohol (*Z*)-**1a**, as a model candidate for the intramolecular oxaallyl alkylation. Our choice was dictated by the well-known reluctance of disubstituted malonyl derivatives to provide vinylbutyrolactones. This aspect was convincingly highlighted in a recent report by Chen and coworkers that described an analogous catalytic approach based on allyl acetate derivatives [41].

At this stage, an extended survey of reaction parameters (metal source, solvent, and temperature) was conducted in order to ascertain the optimal catalytic conditions (Table 1).

**Table 1:** Optimization of the reaction conditions for the lactonization of **1a**.^a^

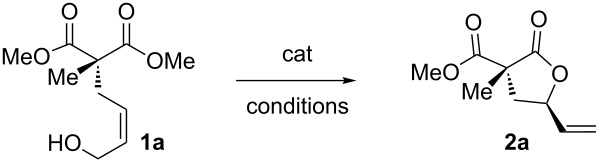				

Entry	Cat (%)	Solvent	Yield (%)^b^	(*trans*:*cis*)^c^

1	[P(*t*-Bu)_2_*o-*biphenyl](AuCH_3_CN)SbF_6_ (5)	DCE	42	nd
2	[P(Cy)_2_*o-*biphenyl-2,4,6(iPr)_3_]AuNTf_2_ (5)	DCE	82	1.5:1
3	PPh_3_AuNTf_2_ (5)	DCE	52	1.1:1
4	[(PPh_3_Au)_3_O]BF_4_ (2)	DCE	Trace	nd
5	AuCl_3_ (5)	DCE	<20	nd
6	[biphepAu_2_Cl_2_/AgOTf] (2.5)	DCE	56	1.3:1
7	[dppf(AuNTf_2_)_2_] (2.5)	DCE	98	1.2:1
8^d^	[dppf(AuNTf_2_)_2_] (0.5)	DCE	96	1.4:1
9^d,e^	IMesAuOTf (5)	DCE	94	2.1:1
10^f^	IMesAuOTf (5)	DCE	Trace	nd
11	IMesAuOTf (5)	Toluene	31	1.9:1
12	IMesAuOTf (5)	CH_3_CN	Trace	nd
13	IMesAuOTf (5)	THF	79	1.9:1
14	AgOTf (5)	DCE	35	nd
15	TsOH (10)	DCE	64	1.3:1
16^d,e,g^	IMesAuOTf (5)	DCE	Trace	nd

^a^All the reactions were carried out under nitrogen atmosphere at 80 °C for 16 h, unless otherwise stated. ^b^Isolated yield after flash chromatography. ^c^Determined by GC on the reaction crude. The relative configuration was determined by NOE experiments on the single diastereoisomers separated by flash chromatography. ^d^Under no moisture restriction, with reagent-grade solvent. ^e^Reaction time: 4 h. ^f^At room temperature. IMes: 1,3-bis(2,4,6-trimethylphenyl)imidazol-2-ylidene. ^g^In the presence of K_2_CO_3_ (1 equiv). nd: not determined.

Initial attempts to perform the lactonization reaction of **1a** were carried out by means of a silver-free cationic complex [P(*t*-Bu)_2_*o-*biphenyl](AuCH_3_CN)SbF_6_ (5 mol %). The desired butyrolactone **2a** was obtained selectively under reflux in DCE for 16 h (entry 1), although only in low yield. With the less bulky triphenylphosphine ligand, the corresponding cationic gold(I) complex (i.e., PPh_3_AuNTf_2_) led to an increase in the isolated yield up to 52%, although the diastereoselection remained elusive (≈ 1:1, entry 3). After demonstrating that the Au(III) catalysis promoted the cyclization in lower extent compared to the Au(I) counterpart (entry 5 versus entries 1–3), we also observed that dinuclear [dppf(AuNTf_2_)_2_] provided **2a** with almost complete conversion (entry 7). The possibility to reduce the loading of the catalyst (0.5 mol %) further, without the need for moisture restriction, was successfully verified by the isolation of **2a** in 96% isolated yield (entry 8). Interestingly, the diastereoselection of the protocol was slightly improved (up to 2.1:1) and the reaction time shortened to 4 h, by employing the carbene-based gold complex IMesAuCl/AgOTf (5 mol %, entry 9) [42–43]. Therefore, by addressing NHCAuOTf as the optimal catalytic system, the impact of the reaction media on the chemical output of the process was investigated (entries 10–13). Here, although **2a** was also isolated in good yield in reagent-grade THF (yield = 79%, dr = 1.9:1, entry 13), DCE was employed as the solvent of choice.

In order to confirm that the catalysis was indeed due to the presence of gold, a control experiment with AgOTf (5 mol %) was performed on compound **1a**. Under comparable reaction conditions (80 °C, 16 h), lactone **2a** was isolated in poor yield (35%). Finally, the hypothetical cocatalysis by Brønsted acids (BA) was verified by means of experimental controls with TsOH (entry 15), and also in the presence of an acid scavenger (entry 16). Here, the desired cyclic compound **2a** was obtained in lower yield (64%) with concomitant substantial decomposition of the starting allylic alcohol. Such evidence confirms the allylic S_N_1 mechanism for the present methodology [44].

The high chemoselectivity guaranteed by the gold catalysts is worthy of note, as it channels the reaction toward the allylic alkylation mechanism without any contamination deriving from transesterification reactions. This evidence is reasonably rationalized in terms of the high π-acidity and poor oxophilicity of the Au(I) species [37].

With the optimal catalytic systems in hand (IMesAuOTf or [dppf(AuNTf_2_)_2_], DCE, 80 °C), we verified the generality of the method by subjecting a range of malonyl alcohols **1b**–**j** to the gold-catalyzed lactonization (Table 2).

**Table 2:** Proving the scope of the gold-catalyzed intramolecular allylation of **1**.^a^

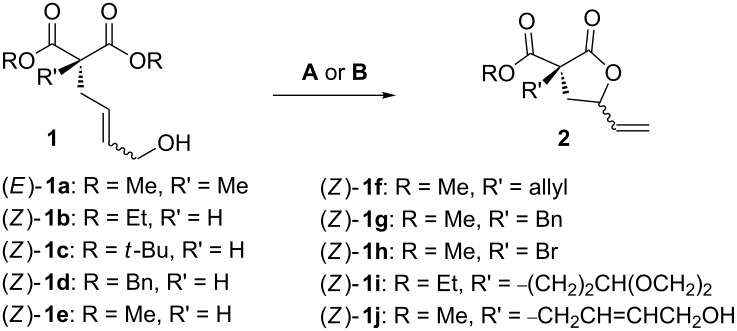					

Entry	**1**	Catalytic system	Product	Yield (%)^b^	*trans*:*cis*^c^

1	(*E*)-**1a**	**A**	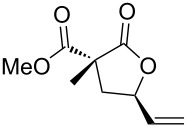 **2a**	72	1.3:1
2	(*Z*)-**1b**	**A**	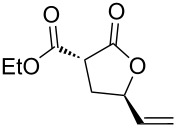 **2b**	95	1:1
3	(*Z*)-**1c**	**A**	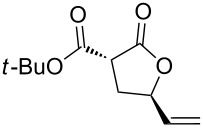 **2c**	66	1.1:1
4	(*Z*)-**1d**	**A**	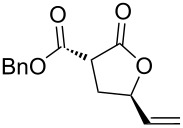 **2d**	67	1.4:1
5	(*Z*)-**1e**	**A**	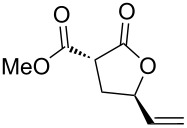 **2e**	85	1:1
6	(*Z*)-**1f**	**A**, **B**	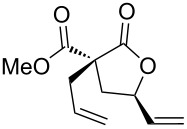 **2f**	94, 35	1.5:1, 1.5:1
7	(*Z*)-**1g**	**A**^d^, **B**^e^	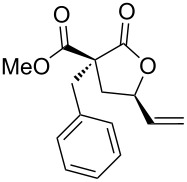 **2g**	63, 12	1:1.4, 1:1.4
8	(*Z*)-**1h**	**A**	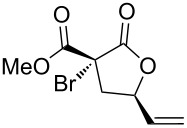 **2h**	54	3.2:1
9	(*Z*)-**1i**	**A**	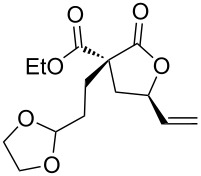 **2i**	45	1.4:1
10	(*Z*)-**1j**	**A**^f^	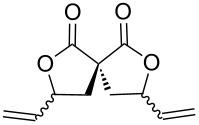 **2j**	93	1.1:1

^a^All the reactions were carried out in reagent-grade solvents under air (80 °C, 0.3 M). Catalytic systems: **A** = IMesAuCl/AgOTf (5 mol %), DCE, 80 °C, 7–9 h. **B** = [dppf(AuNTf_2_)_2_] (2.5 mol %), THF, 80 °C, 16 h. ^b^Isolated yield after flash chromatography. ^c^Determined by GC on the reaction crude. ^d^Dihydronaphthalene derived from undesired Friedel–Crafts alkylation (yield = 14%). ^e^A considerable amount of Friedel–Crafts dihydronaphthalene (yield = 67%) was isolated [23]. ^f^10 mol % of catalyst was used.

The impact of the carbon–carbon double bond configuration on both chemical and stereochemical outputs of the process was initially investigated. Here, by subjecting (*E*)-**1a** to the reaction conditions **A** (i.e., IMesAuCl/AgOTf, DCE, 80 °C) the corresponding lactone **2a** was isolated in comparable yield (72%, entry 1) and similar diastereomeric ratio. Here, although the impact of the C─C double bond configuration on the stereochemical outcome of S_N_2′-type gold-catalyzed intramolecular *O*- [45] and *N-*alkylations [37,46] with allylic alcohols was demonstrated, we consider it likely that an allylic S_N_1 mechanism is involved in the present methodology, due to the similar optical outcomes obtained in the presence of BA metal-free catalysts (entry 15, Table 1).

Then, enolizable substrates carrying different malonyl residues (**1b**–**e**) were taken into account. In all cases the cyclization occurred smoothly leading to the disappearance of the acyclic precursors within 7–9 h reaction time (entries 2–5). Interestingly, in this case no appreciable differences in reaction rate were observed between substrates carrying labile and nonlabile ester alkyl groups.

In some specific cases, both catalytic systems were tested and a direct comparison of performances can be made. Clear evidence was gained for the higher activity of the catalytic system **A** in the expected oxaallylic alkylation process. As an example, when multiple reactive channels were available (i.e., lactonization and Friedel–Crafts-type alkylation, **1g**) dppf-based species (catalytic system **B**) led to a complex mixture of crude reaction products (entry 7), while carbene–gold complex provided mainly the butyrolactone **2g**. Moreover, the methodology proved to be tolerant toward several functional groups/atoms at the methylene carbon atom of the malonyl derivative. In particular, 3,5-*trans*-3-bromo-γ-vinylbutyrolactone **2h** was isolated with 54% yield and in 76:24 diastereoisomeric ratio (entry 8). A protected carbonyl moiety was also tested leading to the corresponding lactone **2i** in moderate yield (45%, entry 9). Finally, the methodology proved to be adaptable allowing a double lactonization event with **1j**, hence providing spiro-lactone **2j** in 93% yield.

**Scheme 1:**
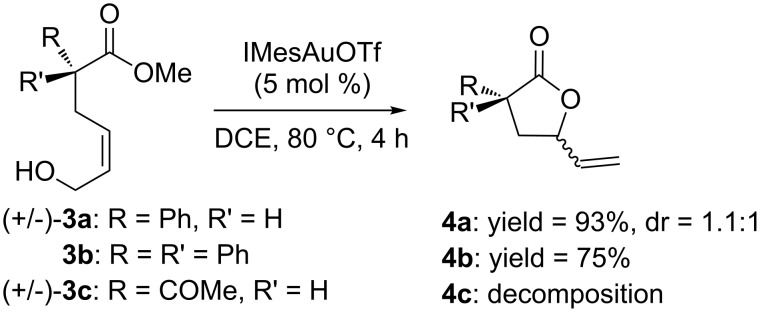
Gold-catalyzed synthesis of γ-lactones **4** from the corresponding monoesters **3**.

Apart from the generality on malonyl substrates, we decided to explore the applicability of the present methodology to less reactive monoester analogs [47]. In this context, readily available alcohols **3a**,**b** were subjected to cyclization in the presence of the gold catalytic system **A**. In both cases lactones **4a**,**b** were isolated in good to excellent yields (93 and 75%, respectively, Scheme 1).

Finally, the 1,3-ketoester **3c** was also subjected to the optimized conditions, but a complex reaction mixture was observed with concomitant decomposition of the starting material.

**Scheme 2:**
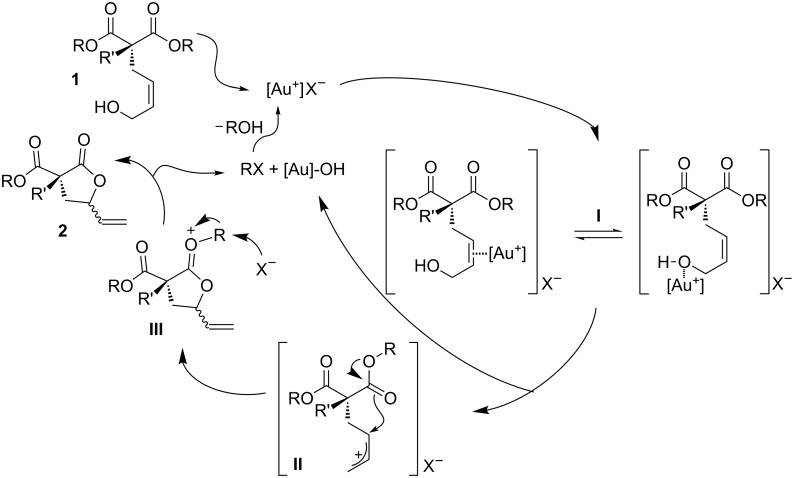
Mechanistic sketch of the gold-promoted oxaallylic alkylation reaction.

The mechanistic proposal for the formation of γ-vinylbutyrolactones **2** is depicted in Scheme 2. As previously mentioned, the formation of an allylic cationic species (**II**) is assumed, upon coordination of the gold catalyst to the allylic alcohol (**I**). In Scheme 2, the possible coordination modes for [Au^+^] to the allylic alcohol are reported. As a matter of fact, although we have previously demonstrated the C=C···Au interaction in the presence of allylic alcohols [16], a concomitant [Au]···OH contact cannot be ruled out [48–49]. Subsequently, the direct nucleophilic attack by the carboxylate unit would lead to an oxonium intermediate **III** [50–51] that, after dealkylation, resulted in the final lactone **2**. Control experiments have been performed to indentify the presence of a Brønsted acid cocatalysis in the ring-closing procedure (see [52] and entry 15 in Table 1). Regeneration of the active cationic gold species or assistance in the formation of the reactive allylic carbocation intermediate **II** are key steps in which the Brønsted cocatalysis could be exerted [52]. Finally, the mandatory role of enol tautomer (or gold–enolate intermediates) [53–55] in the nucleophilic attack was excluded; non-enolizable compounds being suitable candidates for the cyclization reaction.

## Conclusions

In conclusion, we have documented an unprecedented example of gold-catalyzed lactonization with primary allylic alcohols. Cationic NHCAu carbene gold complexes allowed the preparation of a range of functionalized malonyl esters by direct activation of the allylic alcohol by gold. The methodology appears highly chemoselective toward the allylic lactonization, with the possibility to extend the protocol also to acetate derivatives.

## Supporting Information

File 1 Experimental details and characterization of the synthesized compounds.
