# Supplementary material for: Gold(I)-catalyzed synthesis of γ-vinylbutyrolactones by intramolecular oxaallylic alkylation with alcohols
Source: Beilstein J Org Chem. 2011 Sep 1;7:1198–204. doi: 10.3762/bjoc.7.139 (PMC3182428; doi:10.3762/bjoc.7.139)
Supplement: File 1 — Experimental details and characterization of the synthesized compounds. [file Beilstein_J_Org_Chem-07-1198-s001.pdf]

Supporting Information

for

Gold(I)-catalyzed synthesis of γ-vinyl butyrolactones by intramolecular oxaallylic alkylation with alcohols

Michel Chiarucci, Mirko Locritani, Gianpiero Cera and Marco Bandini*

Address: Dipartimento di Chimica Organica “G. Ciamician”, Alma Mater Studiorum – Università di Bologna, Via Selmi 2, 40126 Bologna, Italy

Email: Marco Bandini* - marco.bandini@unibo.it

*Corresponding author

Experimental details and characterization of the synthesized compounds

General methods

^{1}H NMR spectra were recorded on Varian 200 (200 MHz) and Varian 400 (400 MHz) spectrometers. Chemical shifts are reported in ppm from TMS with the solvent resonance as the internal standard (deuterochloroform: δ 7.27 ppm). Data are reported as follows: Chemical shift, multiplicity (s = singlet, d = duplet, , t = triplet, q = quartet, pq = pseudo quartet, pquint = pseudo quintet, b = broad, m = multiplet), coupling constants (Hz). ^{13}C NMR spectra were recorded on Varian 200 (50 MHz) and Varian 400 (100 MHz) spectrometers with complete proton decoupling. Chemical shifts are reported in ppm from TMS with the solvent as the internal standard (deuterochloroform: δ 77.0 ppm). GC–MS spectra were taken by EI ionization at 70 eV on a Hewlett-Packard 5971 with GC injection. They are reported as: *m*/*z* (rel. intense). LC–electrospray ionization mass spectra were obtained with Agilent Technologies MSD1100 single-quadrupole mass spectrometer. Chromatographic purification was performed with 240–400 mesh silica gel. THF was distilled from sodium-benzophenone prior to use. Other anhydrous solvents were supplied by Fluka in Sureseal® bottles and used without any further purification. (*Z*)-allyl bromide **5a** was obtained from commercially available (*Z*)-1,4-but-2-en-ol following a known procedure [1]. The corresponding (*E*)-allyl bromide **5b** was obtained analogously starting from (*E*)-1,4-but-2-en-ol.

General procedures for the synthesis of OTBS-protected alcohols 1a–j′

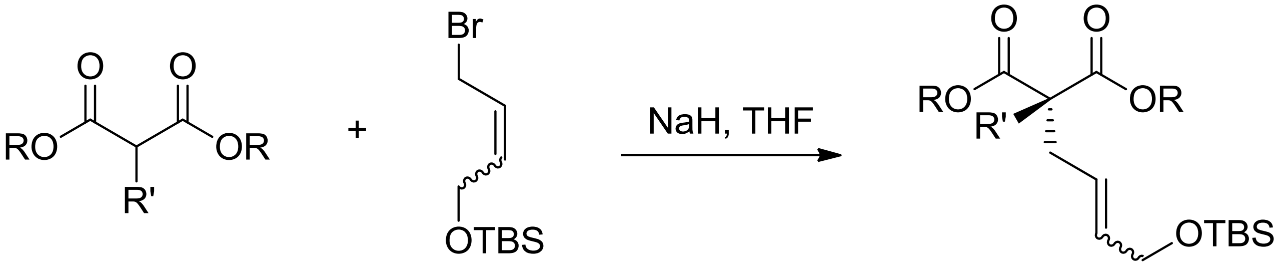

5a-b **1a-j'**

In an oven-dried Schlenk tube, under nitrogen atmosphere, 1.2 mmol (1.2 eq.) of malonate was dissolved in 10 ml of anhydrous THF and cooled to 0 °C. NaH (1.1 mmol, 1.1 eq., 60% dispersion in mineral oil) was added portionwise and the solution was stirred for 30 min at rt. The allyl bromide **5** (1 mmol, 1 eq.) was added at 0 °C and the reaction mixture was stirred overnight at rt. The reaction was quenched with water (10 mL) and extracted with ethyl acetate (3 × 10 ml). The combined organic layers were washed with brine (2 × 10 mL) and dried over Na_2SO_4. The solvent was evaporated at reduced pressure and the crude product was purified with flash chromatography on silica gel eluting with cyclohexane/ethyl acetate, to afford the pure product as a clear oil in variable yield.

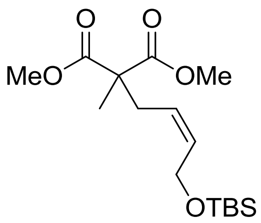

(*Z*)-Dimethyl 2-(4-(*tert*-butyldimethylsilyloxy)but-2-en-1-yl)-2-methylmalonate (1a′): Flash chromatography (*c*-Hex:AcOEt = 90:10). Yield = 84%. ^{1}H NMR (200 MHz, $CDCl_3$): δ 5.73–5.62 (m, 1H), 5.38–5.24 (m, 1H), 4.21 (d, *J* = 6.0 Hz, 2H), 3.73 (s, 6H), 2.63 (d, *J* = 7.6 Hz, 2H), 1.43 (s, 3H), 0.90 (s, 9H), 0.07 (s, 6H). GC–MS (*m*/*z*): 315 (2) $[M - CH_3]^+$, 299 (3), 241 (11), 213 (8), 203 (8), 181 (8), 165 (8), 139 (23), 127 (13), 107 (24), 89 (100), 79 (55), 59 (36).

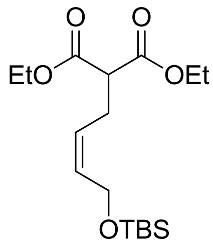

(*Z*)-Diethyl 2-(4-(*tert*-butyldimethylsilyloxy)but-2-en-1-yl)malonate (1b′): Flash chromatography (*c*-Hex:AcOEt = 90:10). Yield = 46%. ^{1}H NMR (200 MHz, $CDCl_3$): δ 5.65–5.57 (m, 1H), 5.44–5.31 (m, 1H), 4.27–4.15 (m, 6H), 3.37 (t, *J* = 7.6 Hz, 1H), 2.65 (dd, *J* = 7.6 Hz, *J* = 7.6 Hz, 1H), 1.27 (t, *J* = 7.2 Hz, 6H), 0.90 (s, 9H), 0.08 (s, 6H). GC–MS (*m*/*z*): 329 (2) $[M - Me]^+$, 299 (10), 287 (100) $[M - t\text{-Bu}]^+$, 241 (37), 213 (12), 185 (22), 169 (24), 151 (17), 139 (40), 121 (40), 95 (43).

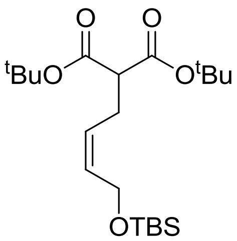

(*Z*)-Di-*tert*-butyl 2-(4-(*tert*-butyldimethylsilyloxy)but-2-en-1-yl)malonate (1c′): Flash chromatography (*c*-Hex:Et_2O = 90:10). Yield = 80%. ^{1}H NMR (400 MHz, $CDCl_3$): δ 5.61–5.57 (m, 1H), 5.41–5.34 (m, 1H), 4.26 (d, *J* = 6.0 Hz, 2H), 3.16 (t, *J* = 7.2 Hz, 1H), 2.55 (t, *J* = 7.2 Hz, 2H), 1.46 (s, 18H), 0.90 (s, 9H), 0.07 (s, 6H). GC–MS (*m*/*z*): 287 (5), 271 (18), 231 (34), 213 (82), 185 (11), 169 (36), 139 (9), 75 (45), 57 (100).

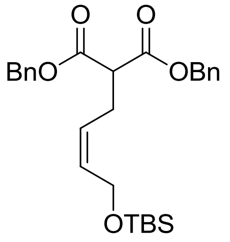

(*Z*)-Dibenzyl 2-(4-(*tert*-butyldimethylsilyloxy)but-2-en-1-yl)malonate (1d′): Flash chromatography (*c*-Hex:Et_2O = 95:5). Yield = 27%. ^{1}H NMR (200 MHz, $CDCl_3$): δ 7.40–7.28 (m, 10 H), 5.71–5.59 (m, 1H), 5.47–5.34 (m, 1H), 5.18 (s, 4H), 4.25 (d, *J* = 5. Hz, 2H), 3.57 (t, *J* = 7.4 Hz, 1H), 2.73 (t, *J* = 7.4 Hz, 2H), 0.94 (s, 9H), 0.10 (s, 6H).

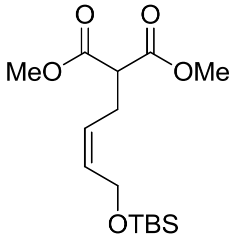

(*Z*)-Dimethyl 2-(4-(*tert*-butyldimethylsilyloxy)but-2-en-1-yl)malonate (1e′): Flash chromatography (*c*-Hex:AcOEt = 90:10). Yield = 40%. ^{1}H NMR (400 MHz, $CDCl_3$): δ 5.65–5.62 (m, 1H), 5.38–5.35 (m, 1H), 4.25 (d, *J* = 6.4 Hz, 2H), 3.75 (s, H), 3.42 (t, *J* = 7.6 Hz, 1H), 2.66 (t, *J* = 7.6 Hz, 2H), 0.91 (s, 9H), 0.08 (s, 6H). ESI-MS (*m*/*z*): 339 $[M + Na]^+$.

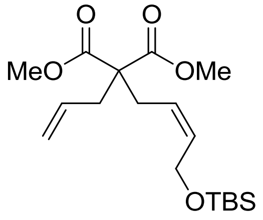

(*Z*)-Dimethyl 2-allyl-2-(4-(*tert*-butyldimethylsilyloxy)but-2-en-1-yl)-malonate (1f′): Flash chromatography (*c*-Hex:AcOEt = 80:20). Yield = 87%. ^{1}H NMR (200 MHz, $CDCl_3$): δ 5.75–5.58 (m, 2H), 5.33–5.20 (m, 1H), 5.14–5.07 (m, 2H), 4.20 (d, *J* = 6.0 Hz, 2H), 3.72 (s, 6H), 2.64 (d, *J* = 7.4 Hz, 4H), 0.90 (s, 9H), 0.07 (s, 6H). GC–MS (*m*/*z*): 341 (2) $[M – Me]^+$, 299 (100) $[M – t\text{-}Bu]^+$, 229 (11), 207 (18), 187 (23), 165 (18), 133 (23), 105 (76), 89 (86), 73 (50).

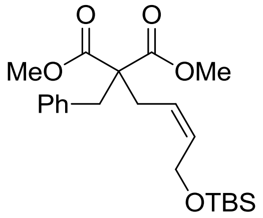

(*Z*)-Dimethyl 2-benzyl-2-(4-(*tert*-butyldimethylsilyloxy)but-2-en-1-yl)malonate (1g′): Flash chromatography (*c*-Hex:AcOEt = 80:20). Yield = 54%. ^{1}H NMR (200 MHz, $CDCl_3$): δ 7.32–7.27 (m, 3H), 7.12–7.08 (m, 2H), 5.76–5.71 (m, 1H), 5.45–5.40 (m, 1H), 4.21 (d, *J* = 6.2 Hz, 2H), 3.76 (s, 6H), 3.29 (s, 2H), 2.58 (d, *J* = 7.4 Hz, 2H), 0.94 (s, 9H), 0.10 (s, 6H). GC–MS (*m*/*z*): 349 (59) $[M – t\text{-}Bu]^+$, 183 (18), 155 (27), 129 (14), 115 (14), 73 (27), 59 (18).

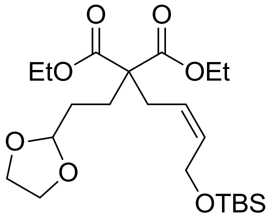

(*Z*)-Diethyl 2-(2-(1,3-dioxolan-2-yl)ethyl)-2-(4-(*tert*-butyldimethylsilyloxy)but-2-en-1-yl)malonate (1i′): Flash chromatography (*c*-Hex:AcOEt = 90:10). Yield = 57%. ^{1}H NMR (200 MHz, $CDCl_3$): δ 5.70–5.58 (m, 1H), 5.36–5.22 (m, 1H), 4.86 (t, *J* = 4.4 Hz, 1H), 4.23–4.13 (m, 6H), 4.00–3.78 (m, 4H), 2.64 (d, *J* = 6.6 Hz, 2H), 2.04–1.96 (m, 2H), 1.63–1.54 (m, 2H), 1.25 (t, *J* = 7.0 Hz, 6H), 0.90 (s, 9H), 0.07 (s, 6H).

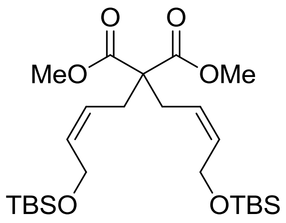

Dimethyl 2,2-bis((*Z*)-4-(*tert*-butyldimethylsilyloxy)but-2-en-1-yl)-malonate (1j′): Flash chromatography (*c*-Hex:Et_2O = 90:10). Yield = 95%. ^{1}H NMR (200 MHz, $CDCl_3$): δ 5.69–5.60 (m, 2H), 5.31–5.24 (m, 2H), 4.20 (d, *J* = 5.6 Hz, 4H), 3.72 (s, 6H), 2.65 (d, *J* = 7.0 Hz, 4H), 0.90 (s, 18H), 0.06 (s, 12H). GC–MS (*m*/*z*): 485 (2) $[M – Me]^+$, 443 (100) $[M – t\text{-}Bu]^+$, 219 (5), 189 (18), 147 (18), 117 (27), 89 (64), 73 (73).

Synthesis of (*Z*)-dimethyl 2-bromo-2-(4-(*tert*-butyldimethylsilyloxy)but-2-en-1-yl) malonate (1h′) [2]: In an oven-dried Schlenk tube, under nitrogen atmosphere, 1.0 mmol (1.0 eq.) of methyl 2-bromomalonate and 1.2 mmol (1.2 eq.) of allyl bromide **5a** were dissolved in 3 mL of anhydrous DMF. K_2CO_3 (1.1 mmol, 1.1 eq.) was added and the reaction mixture was stirred overnight at rt. The solution was diluted with water (5 mL) and extracted with ethyl acetate (3 × 5 mL). The combined organic layers were washed with brine (2 × 10 mL) and dried over Na_2SO_4.The solvent was evaporated at reduced pressure and the crude product was purified with flash chromatography

on silica gel eluting with cyclohexane/ethyl acetate 95:5, affording the pure product as a clear oil (Yield 73%).

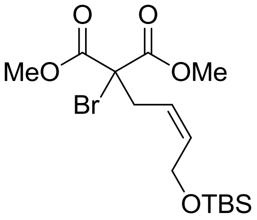

(*Z*)-Dimethyl 2-bromo-2-(4-(*tert*-butyldimethylsilyloxy)but-2-en-1-yl) malonate (1h′): ^{1}H NMR (200 MHz, $CDCl_3$): δ 5.78–5.70 (m, 1H), 5.46–5.36 (m, 1H), 4.24 (d, *J* = 6.2 Hz, 2H), 3.83 (s, 6H), 3.09 (d, *J* = 7.2 Hz, 2H), 0.92 (s, 9H), 0.08 (s, 6H). GC–MS (*m*/*z*): 339 (45), 337 (41) [*M* – *t*-Bu]$^+$, 226 (50), 189 (18), 151 (25), 137 (23), 109 (23), 89 (100), 73 (59), 59 (64).

General procedures for the synthesis of OTBS-protected alcohols 3a–b′: In an oven-dried, three-necked round bottom flask, diisopropylamine (2.1 mmol, 1.05 eq.) was dissolved in 2 mL of anhydrous THF and cooled to 0 °C. BuLi (hexane solution 2.5 M, 2.1 mmol, 1.05 eq.) was added and the solution was stirred for 30 min at 0 °C. Then methyl 2-phenylacetate or methyl 2,2-diphenylacetate was dissolved in 1 mL of THF and added dropwise to the reaction mixture at −78 °C. The solution was stirred at this temperature for 30 min; then the bromide **5a** was added (2.2 mmol, 1.1 eq.) and the mixture was warmed to rt and stirred overnight. The reaction was quenched with saturated ammonium chloride (3 mL) and extracted with Et_2O. The combined organic layers were washed with brine and dried over Na_2SO_4. The solvent was evaporated at reduced pressure and the crude product was purified with flash chromatography on silica gel eluting with cyclohexane/ethyl acetate.

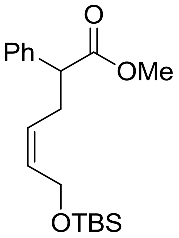

(*Z*)-Methyl 6-(*tert*-butyldimethylsilyloxy)-2-phenylhex-4-enoate (3a′): Flash chromatography (*c*-Hex:Et_2O = 95:5). Yield = 42%. ^{1}H NMR (400 MHz, $CDCl_3$): δ 7.35–7.25 (m, 5H), 5.59–5.52 (m, 1H), 5.37–5.29 (m, 1H), 4.21–4.09 (m, 2H), 3.67 (s, 3H), 3.59 (t, *J* = 7.6 Hz, 1H), 2.82 (pquint, *J* = 7.6 Hz, 1H), 2.52 (pquint, *J* = 7.6 Hz, 1H), 0.90 (s, 9H), 0.06 (s, 6H).

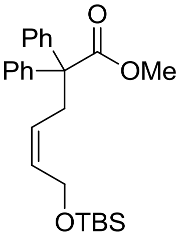

(*Z*)-Methyl 6-(*tert*-butyldimethylsilyloxy)-2,2-diphenylhex-4-enoate (3b′): Flash chromatography (*c*-Hex:Et_2O = 90:10). Yield = 36%. ^{1}H NMR (200 MHz, $CDCl_3$): δ 7.31–7.23 (m, 10H), 5.51–5.43 (m, 1H), 5.37–5.28 (m, 1H), 3.85 (d, *J* = 4.8 Hz, 2H), 3.70 (s, 3H), 3.13 (d, *J* = 7.0 Hz, 2H), 0.87 (s, 9H), 0.06 (s, 6H). GC–MS (*m*/*z*): 395 (2) [*M* – Me]$^+$, 353 (100) [*M* – *t*-Bu]$^+$, 247 (14), 19 (36), 197 (32), 165 (73), 141 (27), 105 (42), 89 (69), 73 (50), 57 (23).

General procedure for the removal of TBS group

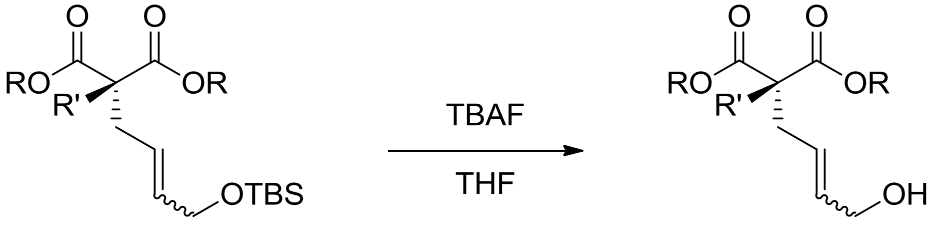

1a-j' **1a-j**

In a round bottom flask the *O*-TBS protected alcohol **1a–j′** (1 mmol, 1 eq.) was dissolved in 10 mL of THF. TBAF (1.2 mmol, 1.2 eq.) was added to the solution at 0 °C and the reaction mixture was stirred at rt until complete consumption of the starting material (TLC, 4–6 h). The solution was

diluted with water (10 mL) and extracted with ethyl acetate (3 × 10 mL). The combined organic layers were washed with brine (2 × 10 mL) and dried over $NaSO_4$. The solvent was evaporated at reduced pressure and the crude product purified by flash chromatography on silica gel (eluent: cyclohexane/ethyl acetate), to afford the pure product as a clear oil.

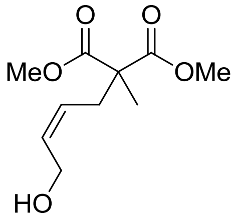

(*Z*)-Dimethyl-2-(4-hydroxybut-2-en-1-yl)-2-methylmalonate (1a): Flash chromatography (*c*-Hex:AcOEt = 70:30). Yield = 84%. ^{1}H NMR (400 MHz, $CDCl_3$): δ 5.82–5.76 (m, 1H), 5.51–5.44 (m, 1H), 4.20 (t, *J* = 6.0 Hz, 2H), 3.74 (s, 6H), 2.67 (d, *J* = 7.6 Hz, 2H), 1.59 (t, *J* = 6.0 Hz, 1H), 1.45 (s, 3H). ^{13}C NMR (100 MHz, $CDCl_3$): δ 172.3 (2C), 132.4, 126.0, 58.2, 53.7 (2C), 52.6 (2C), 33.5, 20.0. ESI–MS (*m*/*z*): 239 [*M* + Na]. Anal. calcd for ($C_{10}H_{16}O_5$: 216.10): C, 55.55; H, 7.46. Found: C, 55.50; H, 7.38.

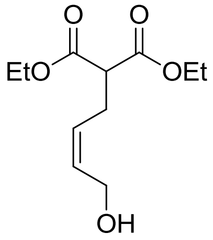

(*Z*)-Diethyl 2-(4-hydroxybut-2-en-1-yl)malonate (1b): Flash chromatography (*c*-Hex:AcOEt = 80:20). Yield = 66%. ^{1}H NMR (200 MHz, $CDCl_3$): δ 5.83–5.71 (m, 1H), 5.55–5.32 (m, 1H), 4.21 (q, *J* = 7.2 Hz, 6H), 3.42 (t, *J* = 7.6 Hz, 1H), 2.71 (t, *J* = 7.6 Hz, 2H), 1.85 (bs, 1H), 1.28 (t, *J* = 7.2 Hz, 6H). ^{13}C NMR (100 MHz, $CDCl_3$): δ 169.0 (2C), 131.6, 127.7, 61.6 (2C), 58.0, 51.6, 26.9, 14.0 (2C). GC–MS (*m*/*z*): 212 (2) $[M - H_2O]^+$, 161 (50), 139 (50), 125 (20), 111 (36), 87 (36), 67 (100), 53 (17). Anal. calcd for ($C_{11}H_{18}O_5$: 230.12): C, 57.38; H, 7.88. Found: C, 57.25; H, 7.80.

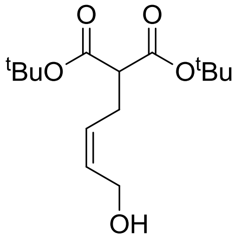

(*Z*)-Di-*tert*-butyl-2-(4-hydroxybut-2-en-1-yl)malonate (1c): Flash chromatography (*c*-Hex:AcOEt = 70:30). Yield = 52%. ^{1}H NMR (400 MHz, $CDCl_3$): δ 5.78–5.72 (m, 1H), 5.51–5.45 (m, 1H), 4.09 (bd, *J* = 5.2 Hz, 2H), 3.22 (t, *J* = 7.2 Hz, 1H), 2.61 (t, *J* = 7.2 Hz, 2H), 1.41 (s, 18H). ^{13}C NMR (100 MHz, $CDCl_3$): δ 168.5 (2C), 131.1, 128.4, 81.9 (2C), 58.1, 53.4, 27.9, 26.6 (6C). ESI-MS: 309 $[M + Na]^+$. Anal. calcd for ($C_{15}H_{26}O_5$: 286.18): C, 62.91; H, 9.15. Found: C, 62.85; H, 9.12.

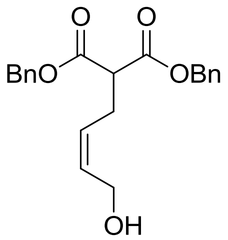

(*Z*)-Dibenzyl-2-(4-hydroxybut-2-en-1-yl)malonate (1d): Flash chromatography (*c*-Hex:AcOEt = 70:30). Yield = 22%. ^{1}H NMR (400 MHz, $CDCl_3$): δ 7.38–7.26 (m, 10H), 5.75–5.69 (m, 1H), 5.49–5.42 (m, 1H), 5.15 (s, 4H), 4.15 (d, *J* = 6.8 Hz, 2H), 3.54 (t, *J* = 7.6 Hz, 1H), 2.74 (t, *J* = 7.6 Hz, 2H). ^{13}C NMR (100 MHz, $CDCl_3$): δ 168.7 (2C), 135.1 (2C), 131.9, 128.6 (4C), 128.4 (4C), 128.2 (2C), 127.4, 67.3 (2C), 58.1, 51.7, 26.7. Anal. calcd for ($C_{21}H_{22}O_5$: 354.15): C, 71.17; H, 6.26. Found: C, 71.15; H, 6.18.

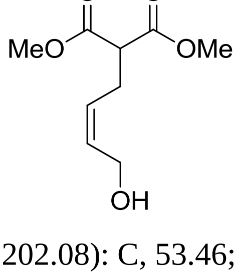

(*Z*)-Dimethyl-2-(4-hydroxybut-2-en-1-yl)malonate (1e): Flash chromatography (*c*-Hex:AcOEt = 80:20). Yield = 38%. ^{1}H NMR (400 MHz, $CDCl_3$): δ 5.80–5.74 (m, 1H), 5.50–5.44 (m, 1H), 4.20 (d, *J* = 7.2 Hz, 2H), 3.75 (s, 6H), 3.47 (t, *J* = 7.6 Hz, 1H), 2.72 (t, *J* = 7.6 Hz, 2H). ^{13}C NMR (100 MHz, $CDCl_3$): δ 169.3 (2C), 131.8, 127.2, 57.9, 52.9 (2C), 51.3. GC–MS (*m*/*z*): 184 (5) $[M - H_2O]^+$, 171 (1), 152 (9),145 (9), 133 (100), 125 (50), 109 (36), 101 (64), 93 (32), 81 (32), 69 (64), 59 (51), 53 (32). Anal. calcd for ($C_9H_{14}O_5$: 202.08): C, 53.46; H, 6.98. Found: C, 53.39; H, 6.91.

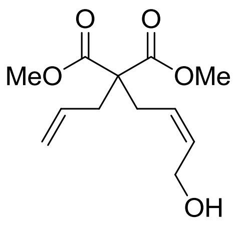

(*Z*)-Dimethyl-2-allyl-2-(4-hydroxybut-2-en-1-yl)malonate (1f): Flash chromatography (*c*-Hex:AcOEt = 70:30). Yield = 74%. ^{1}H NMR (400 MHz, $CDCl_3$): δ 5.80–5.74 (m, 1H), 5.71–5.60 (m, 1H), 5.45–5.35 (m, 1H), 5.12 (d, *J* = 15.6 Hz, 1H), 5.12 (d, *J* = 115.6 Hz, 1H), 4.17 (d, *J* = 6.8 Hz, 2H), 3.73 (s, 6H), 2.67 (t, *J* = 7.2 Hz, 4H). ^{13}C NMR (100 MHz, $CDCl_3$): δ 171.3 (2C), 132.4, 131.2, 125.6, 119.4, 58.3 (2C), 57.7, 52.5, 37.3, 30.6. GC–MS (*m*/*z*): 225 (3), 201 (3), 192 (3), 172 (27), 164 (32), 151 (45), 137 (41), 123 (18), 108 (100), 91 (41), 79 (54), 67 (27), 59 (56). Anal. calcd for ($C_{12}H_{18}O_5$: 242.12): C, 59.49; H, 7.49. Found: C, 59.41; H, 7.41.

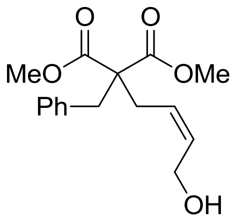

(*Z*)-Dimethyl 2-benzyl-2-(4-hydroxybut-2-en-1-yl)malonate (1g): Flash chromatography (*c*-Hex:AcOEt = 70:30). Yield = 56%.^{1}H NMR (400 MHz, $CDCl_3$): δ 7.30–7.24 (m, 3H), 7.07 (dd, *J* = 8.0 Hz, *J* = 1.6 Hz, 2H), 5.82–5.75 (m, 1H), 5.57–5.49 (m, 1H), 5.12 (t, *J* = 6.0 Hz, 2H), 3.74 (s, 6H), 3.28 (s, 2H), 2.58 (d, *J* = 7.6 Hz, 2H). ^{13}C NMR (100 MHz, $CDCl_3$): δ 171.3 (2C), 135.6, 132.3, 129.8 (2C), 128.4 (2C), 127.1, 125.7, 59.0, 58.3 (2C), 52.5, 38.7, 30.3. GC–MS (*m*/*z*): 221 (9), 214 (14), 189 (18), 169 (9), 155 (27), 130 (23), 115 (18), 65 (23). Anal. calcd for ($C_{16}H_{20}O_5$: 292.13): C, 65.74; H, 6.90. Found: C, 64.71; H, 6.85.

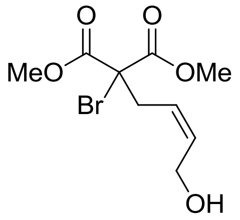

(*Z*)-Dimethyl-2-bromo-2-(4-hydroxybut-2-en-1-yl)malonate (1h): Flash chromatography (*c*-Hex:AcOEt = 70:30). Yield = 13%. ^{1}H NMR (200 MHz, $CDCl_3$): δ 5.93–5.80 (m, 1H), 5.61–5.48 (m, 1H), 4.20 (d, *J* = 6.6 Hz, 2H), 3.83 (s, 6H), 3.11 (d, *J* = 8.2 Hz, 2H). ^{13}C NMR (100 MHz, $CDCl_3$): δ 167.1 (2C), 133.7, 124.6, 60.9, 58.5, 54.0 (2C), 36.3. GC–MS (*m*/*z*): 212 (27), 210 (27), 180 (32), 169 (50), 151 (35), 137 (68), 109 (82), 81 (59), 59 (100). Anal. calcd for ($C_9H_{12}BrO_5$: 279.99): C, 38.45; H, 4.66. Found: C, 38.41; H, 4.61.

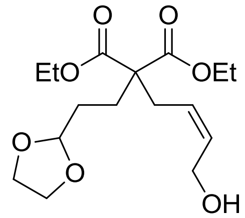

(*Z*)-Diethyl-2-(2-(1,3-dioxolan-2-yl)ethyl)-2-(4-hydroxybut-2-en-1-yl)-malonate (1i): Flash chromatography (*c*-Hex:AcOEt = 50:50). Yield = 90%. ^{1}H NMR (400 MHz, $CDCl_3$): δ 5.81–5.77 (m, 1H), 5.39–5.33 (m, 1H), 4.88 (t, *J* = 4.4 Hz, 1H), 4.21 (q, *J* = 7.2 Hz, 4H), 4.15 (d, *J* = 6.4 Hz, 2H), 3.98–3.96 (m, 2H), 3.87–3.84 (m, 2H), 2.70 (d, *J* = 8.0 Hz, 2H), 2.09 (bt, *J* = 6.4 Hz, 1H), 2.03–1.99 (m, 2H), 1.62–1.57 (m, 2H), 1.26 (t, *J* = 7.2 Hz, 6H). ^{13}C NMR (100 MHz, $CDCl_3$): δ 171.0 (2C), 132.7, 125.2, 103.7, 64.9 (2C), 61.3 (2C), 57.7, 56.7, 29.9, 28.3, 25.9, 14.0 (2C). GC–MS (*m*/*z*): 258 (5), 229 (5), 183 (9), 166 (5), 137 (9), 99 (20), 73 (100), 57 (9). Anal. calcd for ($C_{16}H_{26}O_7$: 330.13): C, 58.17; H, 7.93. Found: C, 58.10; H, 7.85.

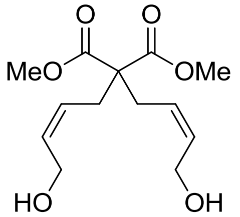

Dimethyl-2,2-bis((*Z*)-4-hydroxybut-2-en-1-yl)malonate (1j): Flash chromatography (*c*-Hex:AcOEt = 80:20). Yield = 83%. ^{1}H NMR (400 MHz, $CDCl_3$): δ 5.75–5.69 (m, 2H), 5.28–5.21 (m, 2H), 4.14 (d, *J* = 6.4 Hz, 4H), 3.74 (s, 6H), 3.08 (bs, 2H), 2.65 (d, *J* = 7.2 Hz, 4H). ^{13}C NMR (100 MHz, $CDCl_3$): δ 171.2 (2C), 132.9 (2C), 124.7 (2C), 58.0 (2C), 57.0, 52.7 (2C), 30.3 (2C). ESI-MS (*m*/*z*): 295 $[M + Na]^+$. Anal. calcd for ($C_{13}H_{20}O_6$: 272.13): C, 57.34; H, 7.40. Found: C, 57.21; H, 7.21.

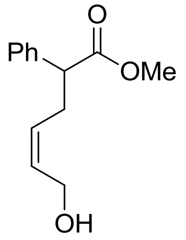

(*Z*)-Methyl 6-hydroxy-2-phenylhex-4-enoate (3a): Flash chromatography (*c*-Hex:AcOEt = 70:30). Yield = 92%. ^{1}H NMR (400 MHz, $CDCl_3$): δ 7.37–7.28 (m, 5H), 5.73–5.66 (m, 1H), 5.51–5.45 (m, 1H), 4.20 (dd, *J* = 12.4 Hz, *J* = 6.8 Hz, 1H), 4.02 (dd, *J* = 12.4 Hz, *J* = 6.8 Hz, 1H), 3.67 (s, 3H), 3.67 (dd, *J* = 8.4 Hz, *J* = 6.8 Hz, 1H), 2.94 (dt, *J* = 14.0 Hz, *J* = 8.4 Hz, 1H), 2.48 (dt, *J* = 14.0 Hz, *J* = 6.8 Hz, 1H), 1.48 (bs, 1H). ^{13}C NMR (100 MHz, $CDCl_3$): δ 174.1, 138.4, 130.9, 129.0, 128.7 (2C), 127.9 (2C), 127.5, 58.2, 52.2, 51.4, 31.7. GC–MS (*m*/*z*): 202 (5) [M – $H_2O]^+$, 188 (5), 151 (50), 143 (100), 121 (82), 91 (64), 77 (27), 65 (14), 51 (14). Anal. calcd for ($C_{13}H_{16}O_3$: 220.11): C, 70.89; H, 7.32. Found: C, 70.78; H, 7.25.

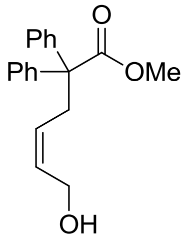

(*Z*)-Methyl 6-hydroxy-2,2-diphenylhex-4-enoate (3b): Flash chromatography (*c*-Hex:AcOEt = 70:30). Yield = 46%.^{1}H NMR (200 MHz, $CDCl_3$): δ 7.39–7.28 (m, 10H), 5.63–5.42 (m, 2H), 3.85 (d, *J* = 6.6 Hz, 2H), 3.73 (s, 3H), 3.19 (d, *J* = 7.4 Hz, 2H). ^{13}C NMR (50 MHz, $CDCl_3$): δ 174.4, 142.3 (2C), 131.5, 129.0 (4C), 127.8 (4C), 127.7 (2C), 127.0, 60.6, 58.2, 52.5, 36.2. ESI-MS (*m*/*z*): 279 $[M – OH]^+$, 335 $[M + K]^+$. Anal. calcd for ($C_{19}H_{20}O_3$: 296.14): C, 77.00; H, 6.80. Found: C, 76.95; H, 6.79.

General procedure for the gold(I)-catalyzed synthesis of vinyl lactones 2a–j or 4a,b

In a screw-capped vial, under air atmosphere, (NHC)AuCl (0.05 eq.), and AgOTf (0.05 eq) were dissolved in 300 µL of reagent-grade dichloroethane (DCE) and the solution was stirred for 30 min at rt in the dark. Then, 20 mg (1 eq.) of allylic alcohol **1a–j** or **3a,b** was added and the reaction mixture stirred at 80 °C until complete consumption of the starting material (TLC, 7–9 h). The crude product was purified with flash chromatography on a short pad of silica gel eluting with diethyl ether or cyclohexane/ethyl acetate mixture, to afford the analytical pure product as an oil.

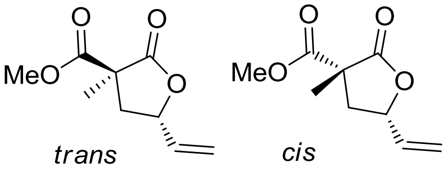

Methyl 3-methyl-2-oxo-5-vinyltetrahydrofuran-3-carboxylate (2a): Flash chromatography (*c*-Hex:AcOEt = 90:10). Yield =94%, dr (*trans*:*cis*) = 2.1:1 (Table 1, entry 1). ^{1}H NMR (400 MHz, $CDCl_3$): δ (*trans*): 5.89–5.82 (m, 1H), 5.43 (dd, *J* = 17.2 Hz, *J* = 1.2 Hz, 1H), 5.31 (dd, *J* = 10.8 Hz, *J* = 1.2 Hz, 1H), 5.00 (m, 1H), 3.80 (s, 3H), 2.87 (dd, *J* = 12.8 Hz, *J* = 6.4 Hz, 1H), 1.92 (dd, *J* = 12.8 Hz, *J* = 10.0 Hz, 1H), 1.55 (s, 3H). δ (*cis*): 5.89–5.82 (m, 1H), 5.41 (d, *J* = 16.4 Hz, 1H), 5.30 (d, *J* = 10.8 Hz, 1H), 4.94 (pq, *J* = 7.2 Hz, 1H), 3.78 (s, 3H), 2.65 (dd, *J* = 12.8 Hz, *J* = 8.0 Hz, 1H), 2.32 (dd, *J* = 12.8 Hz, *J* = 7.2 Hz, 1H), 1.57 (s, 3H). ^{13}C NMR (100 MHz, $CDCl_3$): δ (*trans*): 174.8, 170.8, 135.0, 118.7, 78.4, 53.2, 51.5, 41.4, 20.9. δ (*cis*): 175.1, 171.0, 135.0, 118.5, 78.1, 53.1, 50.8, 40.3, 20.1. GC–MS (*m*/*z*): 169 (2) $[M – Me]^+$, 156 (9), 125 (71), 97 (14), 81 (100), 69 (50).

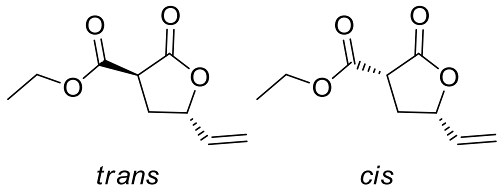

Ethyl 2-oxo-5-vinyltetrahydrofuran-3-carboxylate (2b): Flash chromatography (Et_2O). Yield = 95%, dr (*trans*:*cis*) = 1:1 (Table 2, entry 2). ^{1}H NMR (400 MHz, $CDCl_3$): δ 5.97–5.84 (m, 1H_{cis}, 1H_{trans}), 5.43 (d, *J* = 17.2 Hz, 1H_{trans}), 5.41 (d, *J* = 16.8 Hz, 1H_{cis}), 5.33 (d, *J* = 10.4 Hz, 1H_{trans}), 5.31 (d, *J* = 10.8 Hz, 1H_{cis}), 5.13 (pq, *J* = 6.4 Hz, 1H_{trans}), 4.88 (pq, *J* = 8.0 Hz, 1H_{cis}), 4.27 (q, *J* = 7.2 Hz, 2H_{cis}, 2H_{trans}), 3.66–3.58 (m, 1H_{cis}, 1H_{trans}), 2.80 (ddd, *J* = 12.8 Hz, *J* = 6.4 Hz, *J* = 6.4 Hz, 1H_{trans}), 2.68–2.61 (m, 1H_{cis}), 2.52–2.44 (m,

1H$_{cis}$), 2.25 (ddd, *J* = 12.8 Hz, *J* = 9.2 Hz, *J* = 6.4 Hz, 1H$_{trans}$), 1.33 (t, *J* = 7.2 Hz, 3H$_{cis}$, 3H$_{trans}$). ^{13}C NMR (100 MHz, $CDCl_3$): δ (*trans*): 171.4, 167.5, 134.9, 119.0, 79.5, 62.3, 47.1, 32.4, 14.0. δ (*cis*): 171.6, 167.6, 134.8, 118.1, 79.4, 62.3, 44.3, 32.2, 14.0. GC–MS (*m*/*z*): 184 (2) [*M*]$^+$, 156 (14), 138 (23), 111 (50), 97 (23), 73 (14), 63 (91), 55 (100).

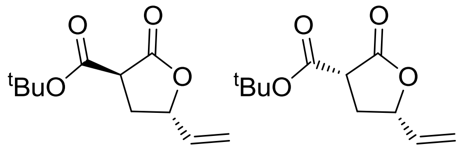

trans *cis*

***tert*-Butyl 2-oxo-5-vinyltetrahydrofuran-3-carboxylate (2c):** Flash chromatography (Et_2O). Yield = 66%. dr (*trans*:*cis*) = 1.1:1 (Table 2, entry 3). ^{1}H NMR (400 MHz, $CDCl_3$): δ 5.98–5.83 (m, 1H$_{cis}$, 1H$_{trans}$), 5.42 (d, *J* = 16.8 Hz, 1H$_{trans}$), 5.40 (d, *J* = 17.6 Hz, 1H$_{cis}$), 5.31 (d, *J* = 10.0 Hz, 1H$_{trans}$), 5.29 (d, *J* = 10.4 Hz, 1H$_{cis}$), 5.10 (pq, *J* = 6.8 Hz, 1H$_{trans}$), 4.88–4.83 (m, 1H$_{cis}$), 3.53 (dd, *J* = 10.4 Hz, *J* = 9.2 Hz, 1H$_{cis}$), 3.50 (dd, *J* = 9.2 Hz, *J* = 6.0 Hz, 1H$_{trans}$), 2.74 (ddd, *J* = 13.2 Hz, *J* = 7.2 Hz, *J* = 6.0 Hz, 1H$_{trans}$), 2.61 (ddd, *J* = 13.2 Hz, *J* = 9.2 Hz, *J* = 6.4 Hz, 1H$_{cis}$), 2.41 (ddd, *J* = 13.2 Hz, *J* = 10.4 Hz, *J* = 9.2 Hz, 1H$_{cis}$), 2.21 (ddd, *J* = 13.2 Hz, *J* = 9.2 Hz, *J* = 6.8 Hz, 1H$_{trans}$), 1.44 (s, 9H$_{cis}$, 9H$_{trans}$). ^{13}C NMR (400 MHz, $CDCl_3$): δ (*trans*): 171.8, 166.8, 135.1, 118.8, 82.9, 79.5, 48.0, 29.7, 27.9. δ (*cis*): 171.9, 166.6, 135.0, 118.0, 83.1, 79.3, 47.4, 29.7, 27.9. GC–MS (*m*/*z*): 157 (9), 139 (18), 121 (9), 95 (7), 64 (32), 57 (100).

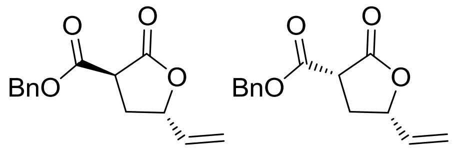

trans *cis*

Benzyl 2-oxo-5-vinyltetrahydrofuran-3-carboxylate (2d): Flash chromatography (*c*-Hex:AcOEt = 90:10). Yield = 67%, dr (*trans*:*cis*) = 1.4:1 (Table 2, entry 4). ^{1}H NMR (400 MHz, $CDCl_3$): δ 7.40–7.35 (m, 5H$_{cis}$, 5H$_{trans}$), 5.96–5.82 (m, 1H$_{cis}$, 1H$_{trans}$), 5.41(d, *J* = 17.2 Hz, 1H$_{trans}$), 5.40 (d, *J* = 17.2 Hz, 1H$_{cis}$), 5.31 (d, *J* = 10.4 Hz, 1H$_{trans}$), 5.30 (d, *J* = 10.4 Hz, 1H$_{cis}$), 5.25 (s, 2H$_{trans}$), 5.24 (s, 2H$_{cis}$), 5.12 (pq, *J* = 6.4 Hz, 1H$_{trans}$), 4.91–4.8 (m, 1H$_{cis}$), 3.77–3.64 (m, 1H$_{cis}$, 1H$_{trans}$), 2.83–2.77 (m, 1H$_{trans}$), 2.68–2.61 (m, 1H$_{cis}$), 2.33–2.44 (m, 1H$_{cis}$), 2.30–2.22 (m, 1H$_{trans}$). GC–MS (*m*/*z*): 178 (1), 140 (23), 122 (14), 107 (43), 91 (100), 77 (14), 65 (32), 54 (36).

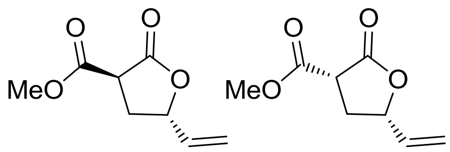

trans *cis*

Methyl 2-oxo-5-vinyltetrahydrofuran-3-carboxylate (2e): Flash chromatography (*c*-Hex:AcOEt = 90:10). Yield = 85%. dr (*trans*:*cis*) = 1:1 (Table 2, entry 5). ^{1}H NMR (400 MHz, $CDCl_3$): δ 5.98–5.83 (m, 1H$_{cis}$, 1H$_{trans}$), 5.43 (d, *J* = 17.2 Hz, 1H$_{trans}$), 5.41 (d, *J* = 17.2 Hz, 1H$_{cis}$), 5.34 (d, *J* = 10.4 Hz, 1H$_{trans}$), 5.31 (d, *J* = 10.8 Hz, 1H$_{cis}$), 5.13 (q, *J* = 6.8 Hz, 1H$_{trans}$), 4.92–4.86 (m, 1H$_{cis}$), 3.83 (s, 3H$_{trans}$), 3.82 (s, 3H$_{cis}$), 3.67 (dd, *J* = 10.8 Hz, *J* = 9.2 Hz, 1H$_{cis}$), 3.63 (dd, *J* = 9.2 Hz, *J* = 6.0 Hz, 1H$_{trans}$), 2.81 (ddd, *J* = 13.2 Hz, *J* = 6.8 Hz, *J* = 6.0 Hz, 1H$_{trans}$), 2.65 (ddd, *J* = 13.2 Hz, *J* = 9.2 Hz, *J* = 6.4 Hz, 1H$_{cis}$), 2.49 (ddd, *J* = 13.2 Hz, *J* = 10.8 Hz, *J* = 6.4 Hz, 1H$_{cis}$), 2.26 (ddd, *J* = 13.2 Hz, *J* = 9.2 Hz, *J* = 6.8 Hz, 1H$_{trans}$). ^{13}C NMR (100 MHz, $CDCl_3$): δ (*trans*): 171.2, 167.9, 134.7, 119.1, 79.6, 53.2, 47.0, 32.4. δ (*cis*): 171.4, 168.0, 134.8, 118.2, 79.5, 53.1, 46.1, 32.2. ESI-MS (*m*/*z*): 193 [*M* + Na]$^+$, 188 [*M* + H_2O]$^+$, 171 [*M* + H]$^+$.

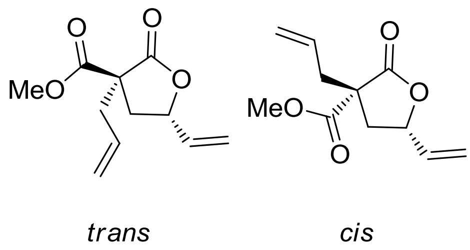

Methyl 3-allyl-2-oxo-5-vinyltetrahydrofuran-3-carboxylate (2f): Flash chromatography (*c*-Hex:AcOEt = 90:10). Yield = 94%. dr (*trans*:*cis*) = 1.5:1 (Table 2, entry 6). ^{1}H NMR (400 MHz, $CDCl_3$): δ 5.95–5.81 (m, 1H$_{cis}$, 1H$_{trans}$), 5.79–5.64 (m, 1H$_{cis}$, 1H$_{trans}$), 5.42 (d, *J* = 17.2 Hz, 1H$_{trans}$), 5.38 (d, *J* = 16.8 Hz, 1H$_{cis}$), 5.31 (d, *J* = 10.4 Hz, 1H$_{trans}$), 5.28 (d, *J* = 10.4 Hz, 1H$_{cis}$), 5.25–5.16 (m, 2H$_{cis}$, 2H$_{trans}$), 5.01–4.95 (m, 1H$_{trans}$), 4.89 (q, *J* = 7.2 Hz, 1H$_{cis}$), 3.81 (s, 3H$_{trans}$), 3.79 (s, 3H$_{cis}$), 2.84–2.74 (m, 3H$_{trans}$), 2.68–2.58 (m, 3H$_{cis}$), 2.48 (dd, *J* = 13.6 Hz, *J* = 7.2 Hz, 1H$_{cis}$), 2.01 (dd, *J* = 13.6 Hz, *J* = 10.0 Hz, 1H$_{trans}$). ^{13}C NMR (100 MHz, $CDCl_3$): δ (*trans*): 173.9, 169.7, 135.1, 131.8, 120.3, 118.8, 78.9, 55.6, 53.3, 38.4, 37.4. δ (*cis*): 174.0, 170.1, 135.4, 131.5, 120.7, 118.2, 78.1, 54.7, 53.2, 38.4, 36.5. GC–MS (*m*/*z*): 178 (5), 160 (32), 133 (32), 105 (50), 79 (100), 54 (77).

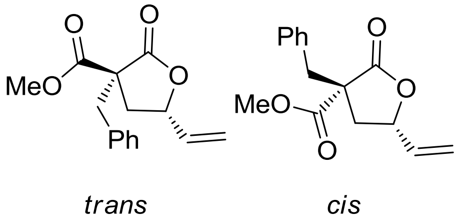

Methyl 3-benzyl-2-oxo-5-vinyltetrahydrofuran-3-carboxylate (2g): Flash chromatography (*c*-Hex:AcOEt = 80:20). Yield = 63%. dr (*trans*:*cis*) = 1:1.4 (Table 2, entry 7). ^{1}H NMR (400 MHz, $CDCl_3$): δ 7.33–7.23 (m, 3H$_{cis}$, 3H$_{trans}$), 7.17 (d, *J* = 6.8 Hz, 2H$_{cis}$), 7.06 (d, *J* = 6.8 Hz, 2H$_{trans}$), 5.78 (ddd, *J* = 17.2 Hz, *J* = 10.4 Hz, *J* = 6.4 Hz, 1H$_{trans}$), 5.55 (ddd, *J* = 17.2 Hz, *J* = 10.4 Hz, *J* = 6.8 Hz, 1H$_{cis}$), 5.30 (d, *J* = 17.2 Hz, 1H$_{trans}$), 5.29 (d, *J* = 10.4 Hz, 1H$_{trans}$), 5.20 (d, *J* = 17.2 Hz, 1H$_{cis}$), 5.19 (d, *J* = 10.4 Hz, 1H$_{cis}$), 4.93–4.87 (m, 1H$_{trans}$), 4.04 (pq, *J* = 7.6 Hz, 1H$_{cis}$), 3.82 (s, 3H$_{cis}$), 3.81 (s, 3H$_{trans}$), 3.42–3.18 (m, 2H$_{cis}$, 2H$_{trans}$), 2.72 (dd, *J* = 13.2 Hz, *J* = 6.4 Hz, 1H$_{trans}$), 2.55 (dd, *J* = 13.6 Hz, *J* = 7.6 Hz, 1H$_{cis}$), 2.47 (dd, *J* = 13.6 Hz, *J* = 7.6 Hz, 1H$_{cis}$), 1.97 (dd, *J* = 13.2 Hz, *J* = 10.0 Hz, 1H$_{trans}$). ^{13}C NMR (100 MHz, $CDCl_3$): δ (*trans*): 173.6, 169.6 135.0, 130.0, 128.8, 128.7, 127.7, 118.0, 79.0, 56.4, 52.5, 39.7, 36.3. δ (*cis*): 174.5, 170.5, 134.9, 130.0, 128.8, 128.7, 127.4, 118.8, 78.1, 57.3, 53.3, 39.5, 37.0. ESI-MS (*m*/*z*): 261 [*M* + H]$^{+}$, 283 [*M* + Na]$^{+}$, 299 [*M* + K]$^{+}$.

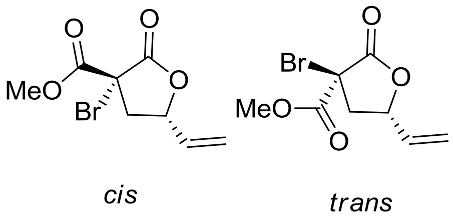

Methyl 3-bromo-2-oxo-5-vinyltetrahydrofuran-3-carboxylate (2h): Flash chromatography (*c*-Hex:AcOEt = 90:10). Yield = 54 %. dr (*cis*:*trans*) = 1:3.2 (Table 2, entry 8). ^{1}H NMR (400 MHz, $CDCl_3$): δ 6.01–5.89 (m, 1H$_{cis}$, 1H$_{trans}$), 5.52 (d, *J* = 17.2 Hz, 1H$_{cis}$), 5.47 (d, *J* = 17.6 Hz, 1H$_{trans}$), 5.41 (d, *J* = 10.4 Hz, 1H$_{cis}$), 5.39 (d, *J* = 10.4 Hz, 1H$_{trans}$), 5.14–5.08 (m, 1H$_{cis}$), 4.99 (pq, *J* = 6.8 Hz, 1H$_{trans}$), 3.92 (s, 3H$_{cis}$), 3.91 (s, 3H$_{trans}$), 3.43 (dd, *J* = 14.0 Hz, *J* = 6.4 Hz, 1H$_{trans}$), 2.91 (dd, *J* = 14.4 Hz, *J* = 9.6 Hz, 1H$_{cis}$), 2.79 (dd, *J* = 14.4 Hz, *J* = 5.2 Hz, 1H$_{cis}$), 2.62 (dd, *J* = 14.0 Hz, *J* = 8.0 Hz, 1H$_{trans}$). ^{13}C NMR (50 MHz, $CDCl_3$): δ (*trans*): 168.8, 165.4, 158.1,133.7, 120.1, 79.1, 54.7, 44.1, 29.7. δ (*cis*): 168.8, 165.4, 132.9, 120.4, 79.0, 54.6, 44.3, 29.7. GC–MS (*m*/*z*): 191 (27), 189 (18) [*M* – COOMe]$^{+}$,137 (45), 93 (100), 79 (28), 65 (86).

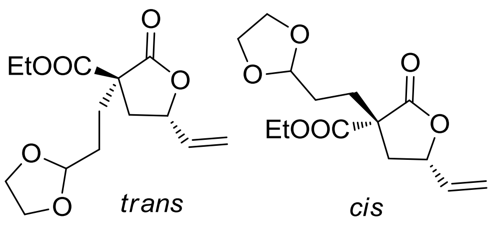

Ethyl 3-(2-(1,3-dioxolan-2-yl)ethyl)-2-oxo-5-vinyltetrahydrofuran-3-carboxylate (2i): Flash chromatography (*c*-Hex:AcOEt = 80:20). Yield = 45 %. dr (*trans*:*cis*) = 1.4:1 (Table 2, entry 9). ^{1}H NMR (400 MHz, $CDCl_3$): δ 5.91 (ddd, *J* = 16.8 Hz, *J* = 10.4 Hz, *J* = 6.4 Hz, 1H$_{cis}$), 5.86 (ddd, *J* = 17.2 Hz, *J* = 10.4 Hz, *J* = 6.8 Hz, 1H$_{trans}$), 5.42 (d, *J* = 17.2 Hz, 1H$_{trans}$), 5.34 (d, *J* = 16.8 Hz,

1H$_{cis}$), 5.31 (d, J = 10.4 Hz, 1H$_{trans}$), 5.28 (d, J = 10.4 Hz, 1H$_{cis}$), 4.99–4.89 (m, 2H$_{cis}$, 2H$_{trans}$), 4.27–4.21 (m, 2H$_{cis}$, 2H$_{trans}$), 3.97–4.96 (m, 2H$_{cis}$, 2H$_{trans}$), 3.89–3.84 (m, 2H$_{cis}$, 2H$_{trans}$), 2.85 (dd, J = 12.8 Hz, J = 6.4 Hz, 1H$_{trans}$), 2.64 (dd, J = 13.2 Hz, J = 6.8 Hz, 1H$_{cis}$), 2.42 (dd, J = 13.2 Hz, J = 7.2 Hz, 1H$_{cis}$), 2.27 (ddd, J = 13.6 Hz, J = 12.4 Hz, J = 4.4 Hz, 1H$_{trans}$), 2.16 (ddd, J = 13.6 Hz, J = 12.4 Hz, J = 4.8 Hz, 1H$_{cis}$), 2.05–1.77 (m, 3H$_{cis}$, 3H$_{trans}$), 1.93 (dd, J = 12.8 Hz, J = 10.0 Hz, 1H$_{trans}$), 1.32 (t, J = 7.2 Hz, 3H$_{trans}$), 1.29 (t, J = 7.2 Hz, 3H$_{cis}$). GC–MS (*m*/*z*): 256 (5), 183 (5), 99 (11), 73 (100), 55 (9).

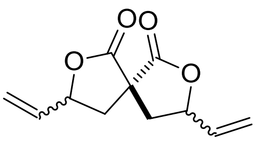 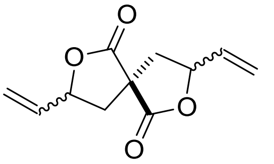

3,8-Divinyl-2,7-dioxaspiro[4.4]nonane-1,6-dione (2j): Flash chromatography (*c*-Hex:AcOEt = 80:20). Yield = 93%. dr: 1.1:1 (two diastereoisomers detected, Table 2, entry 10). ^{1}H NMR (400 MHz, $CDCl_3$): δ 6.05 (ddd, J = 17.6 Hz, J = 10.4 Hz, J = 7.2 Hz, 2H$_{major}$), 5.88 (ddd, J = 17.2 Hz, J = 10.8 Hz, J = 6.8 Hz, 2H$_{minor}$), 5.47 (d, J = 16.8, 2H$_{minor}$), 5.44 (d, J = 16.8, 2H$_{major}$), 5.38–5.28 (m, 2H$_{major}$, 4H$_{minor}$), 4.97 (pq, J = 7.2 Hz, 2H$_{major}$), 2.88 (dd, J = 12.8 Hz, J = 6.0 Hz, 2H$_{minor}$), 2.77 (dd, J = 13.6 Hz, J = 8.4 Hz, 2H$_{major}$), 2.41 (dd, J = 13.6 Hz, J = 7.2 Hz, 2H$_{major}$), 2.10 (dd, J = 12.8 Hz, J = 9.6 Hz, 2H$_{minor}$). GC–MS (*m*/*z*): 208 (2) $[M]^{+}$, 193 (2) 175 (2), 154 (27), 126 (27), 108 (18), 79 (100), 55 (45).

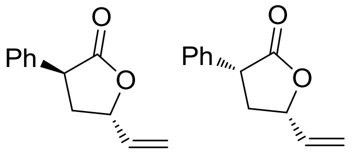

trans *cis*

2-Phenyl-4-vinylbutyrolactone (4a): Flash chromatography (*c*-Hex:AcOEt = 90:10). Yield = 93%. dr (*trans*:*cis*) = 1.1:1. ^{1}H NMR (400 MHz, $CDCl_3$): δ 7.40–7.37 (m, 2H$_{cis}$, 2H$_{trans}$), 7.33–7.29 (m, 3H$_{cis}$, 3H$_{trans}$), 5.98 (dd, J = 17.2 Hz, J = 10.4 Hz, 1H$_{cis}$), 5.96 (dd, J = 16.8 Hz, J = 10.8 Hz, 1H$_{trans}$), 5.47 (d, J = 16.8 Hz, 1H$_{trans}$), 5.44 (d, J = 17.2 Hz, 1H$_{cis}$), 5.34 (d, J = 10.8 Hz, 1H$_{trans}$), 5.33 (d, J = 10.4 Hz, 1H$_{cis}$), 5.14–5.09 (m, 1H$_{cis}$), 4.97–4.92 (m, 1H$_{trans}$), 3.95–3.87 (m, 1H$_{cis}$, 1H$_{trans}$), 2.85 (ddd, J = 12.8 Hz, J = 8.4 Hz, J = 5.6 Hz, 1H$_{trans}$), 2.65–2.49 (m, 2H$_{cis}$), 2.20 (ddd, J = 12.8 Hz, J = 12.8 Hz, J = 10.8 Hz, 1H$_{trans}$). ^{13}C NMR (50 MHz, $CDCl_3$): δ(*trans*): 176.5, 136.3, 129.0, 128.8, 128.2, 127.9, 118.6, 78.7, 47.1, 38.3. δ (*cis*): 177.0, 136.7, 129.1, 128.2, 128.0, 127.8, 117.4, 78.1, 44.9, 36.8. GC–MS (*m*/*z*): 144 (64), 129 (100), 115 (23), 102 (23), 66 (50), 51 (23).

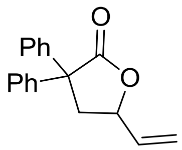

2,2-Diphenyl-4-vinylbutyrolacton (4b): Flash chromatography (*c*-Hex:AcOEt = 90:10). Yield = 75%. ^{1}H NMR (400 MHz, $CDCl_3$): δ 7.40–7.39 (m, 4H), 7.32 (d, J = 4.0 Hz, 6H), 5.94 (ddd, J = 17.2 Hz, J = 10.4 Hz, J = 6.8 Hz, 1H), 5.42 (d, J = 17.2 Hz, 1H), 5.31 (d, J = 10.4 Hz, 1H), 4.78 (pquint, J = 5.2 Hz, 1H), 3.11 (dd, J = 13.2 Hz, J = 5.2 Hz, 1H), 2.77 (dd, J = 13.2 Hz, J = 10.4 Hz, 1H). ^{13}C NMR (100 MHz, $CDCl_3$): δ 176.8, 141.7, 139.6, 134.9, 129.0, 128.4, 127.8, 127.7, 127.3, 127.3, 118.9, 77.5, 58.1, 43.8. GC–MS (*m*/*z*): 220 (64), 205 (50), 191 (18), 165 (54), 143 (50), 129 (100), 115 (36), 91 (32), 77 (23), 51 (18). Anal. calcd for ($C_{18}H_{16}O_2$: 264.12): C, 81.79; H, 6.10. Found: C, 81.70; H, 6.05.

References

1. Oppolzer, W.; Moretti, R.; Zhou, C. *Helv. Chim. Acta* **1994**, *77*, 2663.

2. Odabachian, Y.; Gagosz, F. *Adv. Synt. Cat.* **2009**, *351*, 379. doi: 10.1002/adsc.200900056

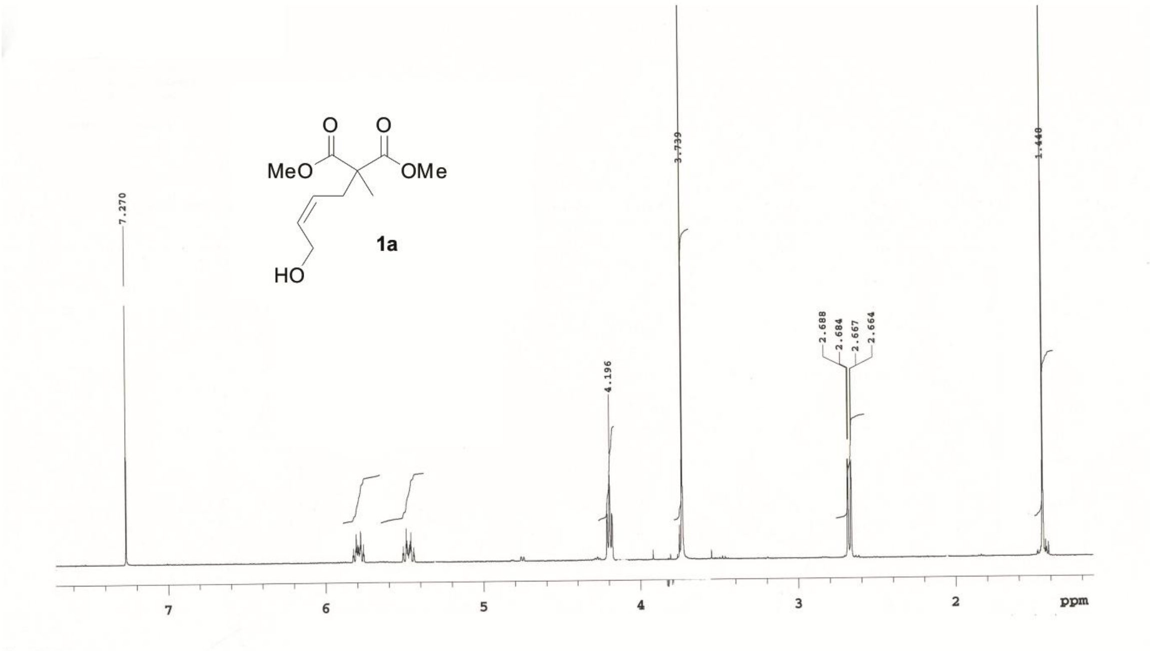

MeO
O
O
OMe
HO
1a
7.270
4.196
3.739
2.688
2.684
2.667
2.664
1.440
7
6
5
4
3
2
ppm

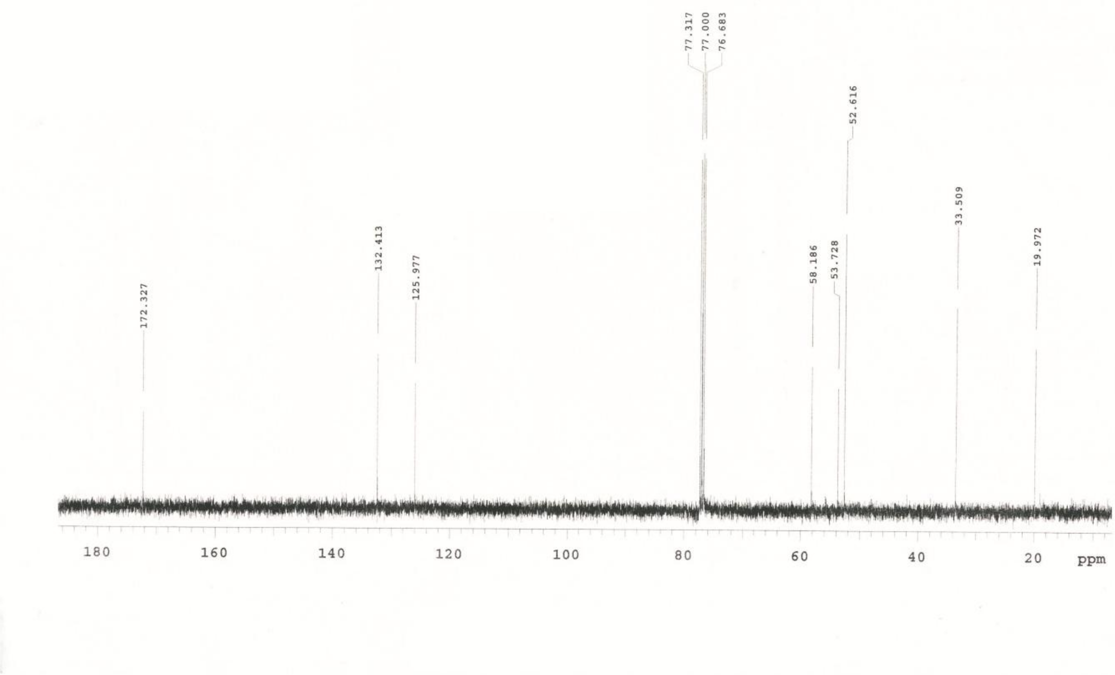

172.327
132.413
125.977
77.317
77.000
76.683
58.186
53.728
52.616
33.509
19.972
180
160
140
120
100
80
60
40
20
ppm

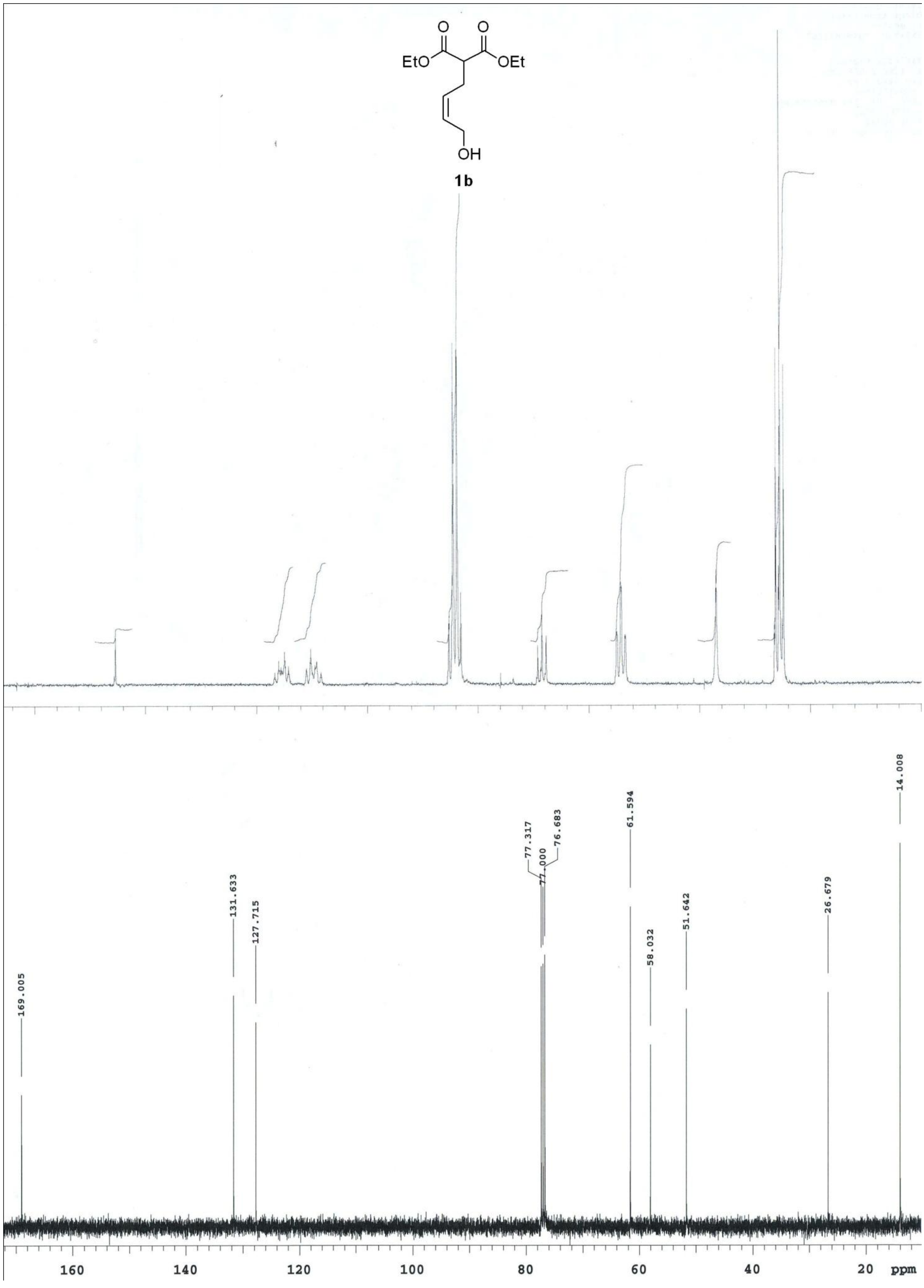
EtO
OEt
O
O
OH
1b
169.005
131.633
127.715
77.317
77.000
76.683
61.594
58.032
51.642
26.679
14.008
160
140
120
100
80
60
40
20
ppm

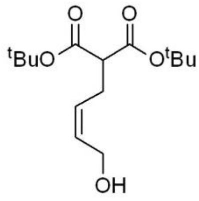

tBuO
OtBu
OH

1c

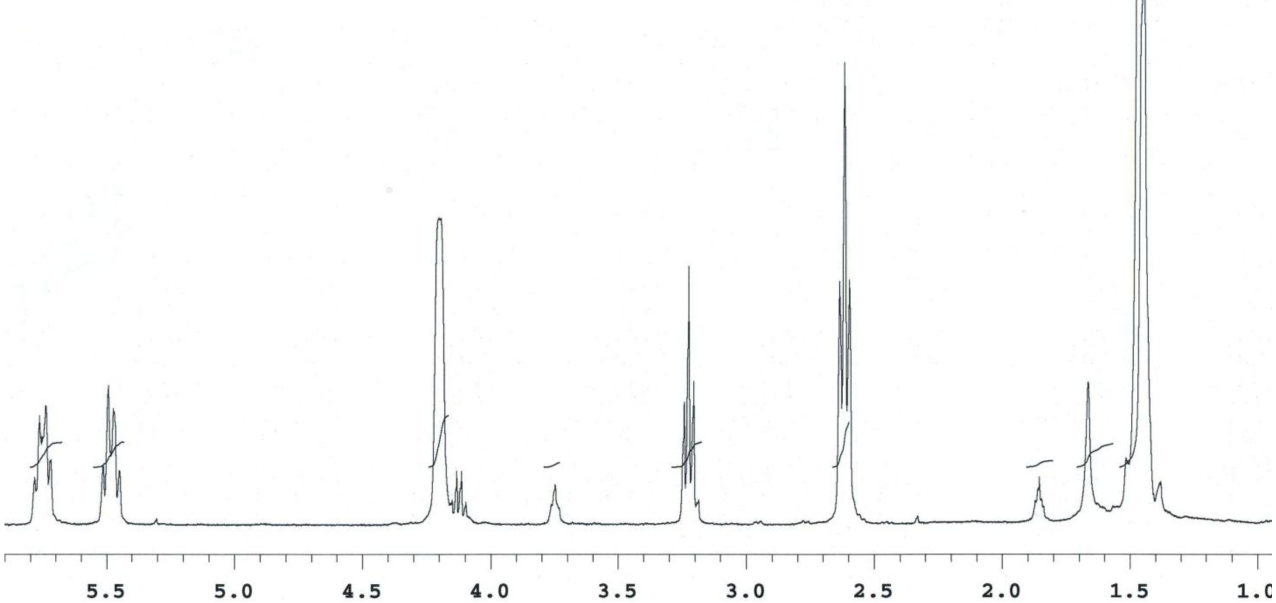

5.5
5.0
4.5
4.0
3.5
3.0
2.5
2.0
1.5
1.0

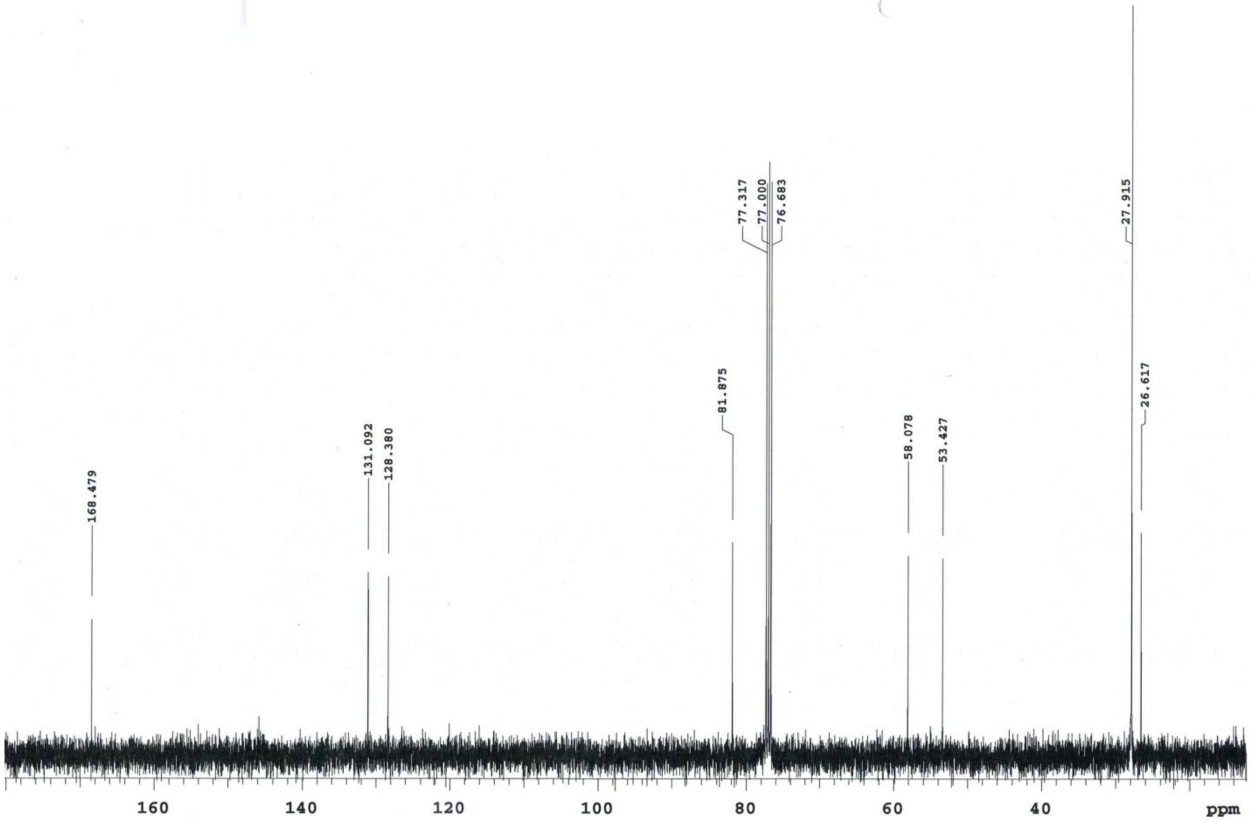

168.479
131.092
128.380
81.875
77.317
77.000
76.683
58.078
53.427
27.915
26.617
160
140
120
100
80
60
40
ppm

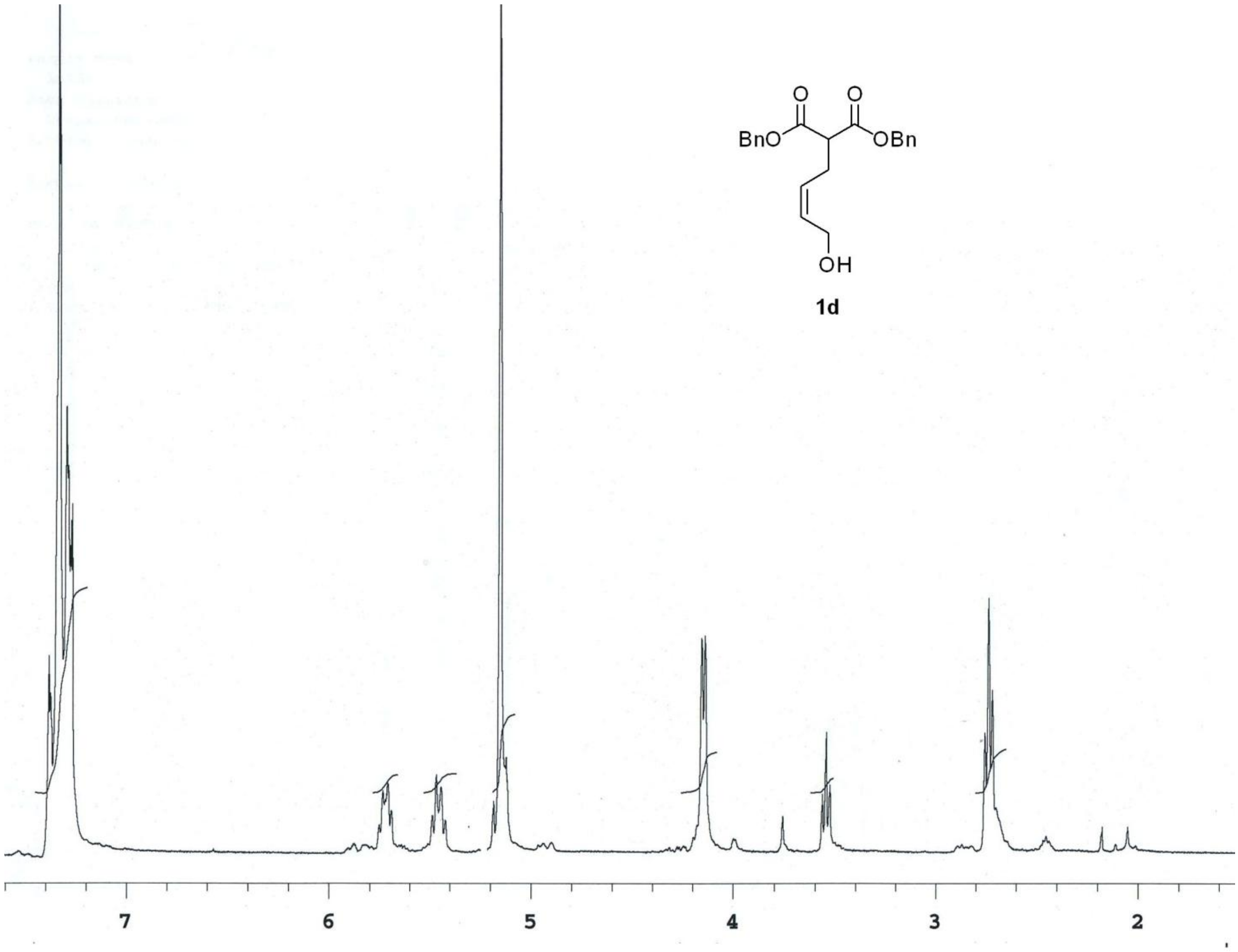
BnO
OBn
OH
1d
7
6
5
4
3
2

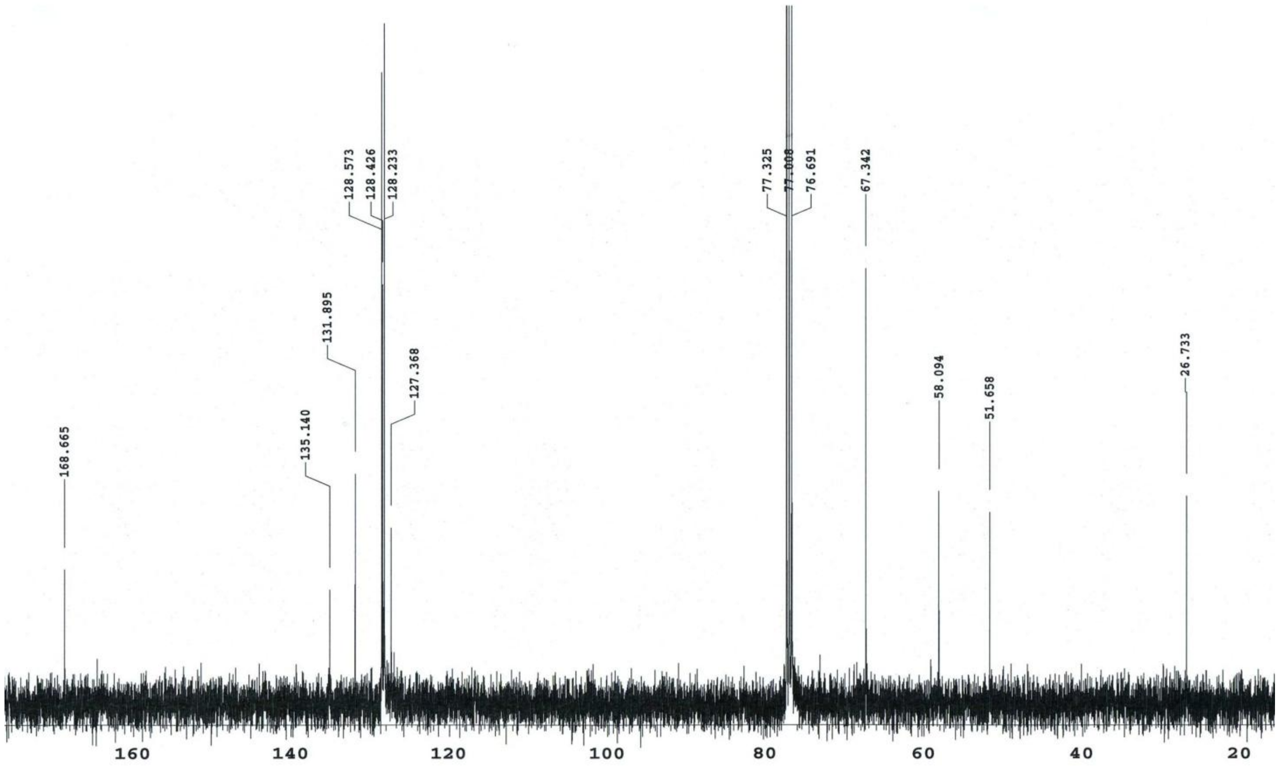
128.573
128.426
128.233
131.895
127.368
135.140
168.665
77.325
77.008
76.691
67.342
58.094
51.658
26.733
160
140
120
100
80
60
40
20

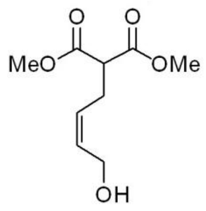
MeO
OMe
O
O
OH

1e

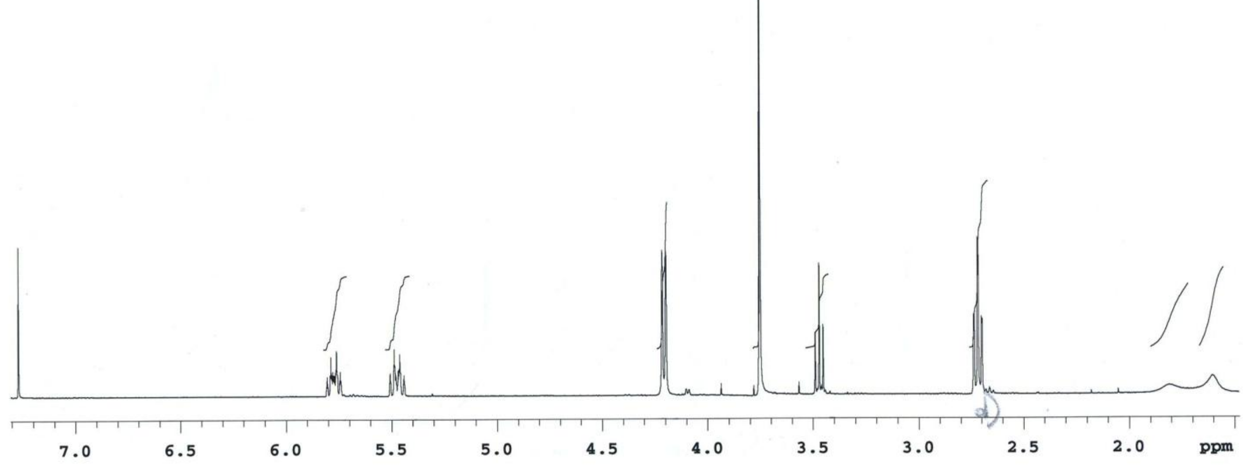
7.0
6.5
6.0
5.5
5.0
4.5
4.0
3.5
3.0
2.5
2.0
ppm

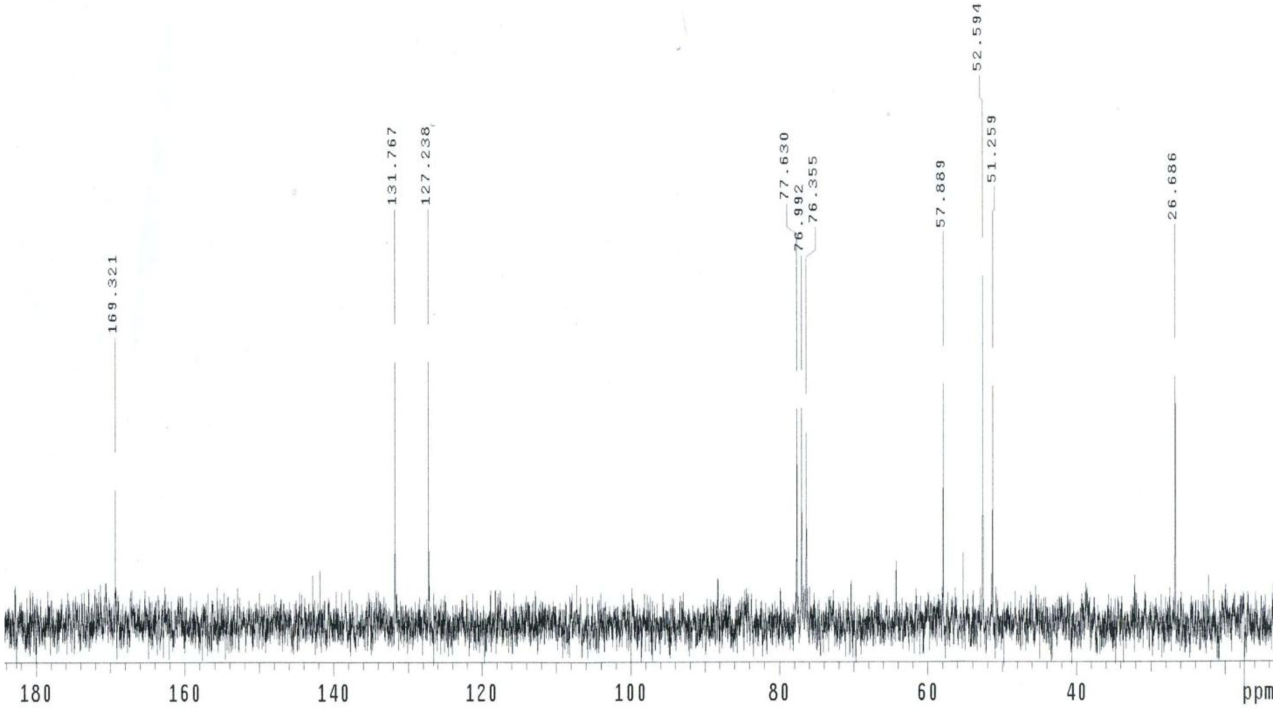
169.321
131.767
127.238
77.630
76.992
76.355
57.889
52.594
51.259
26.686
180
160
140
120
100
80
60
40
ppm

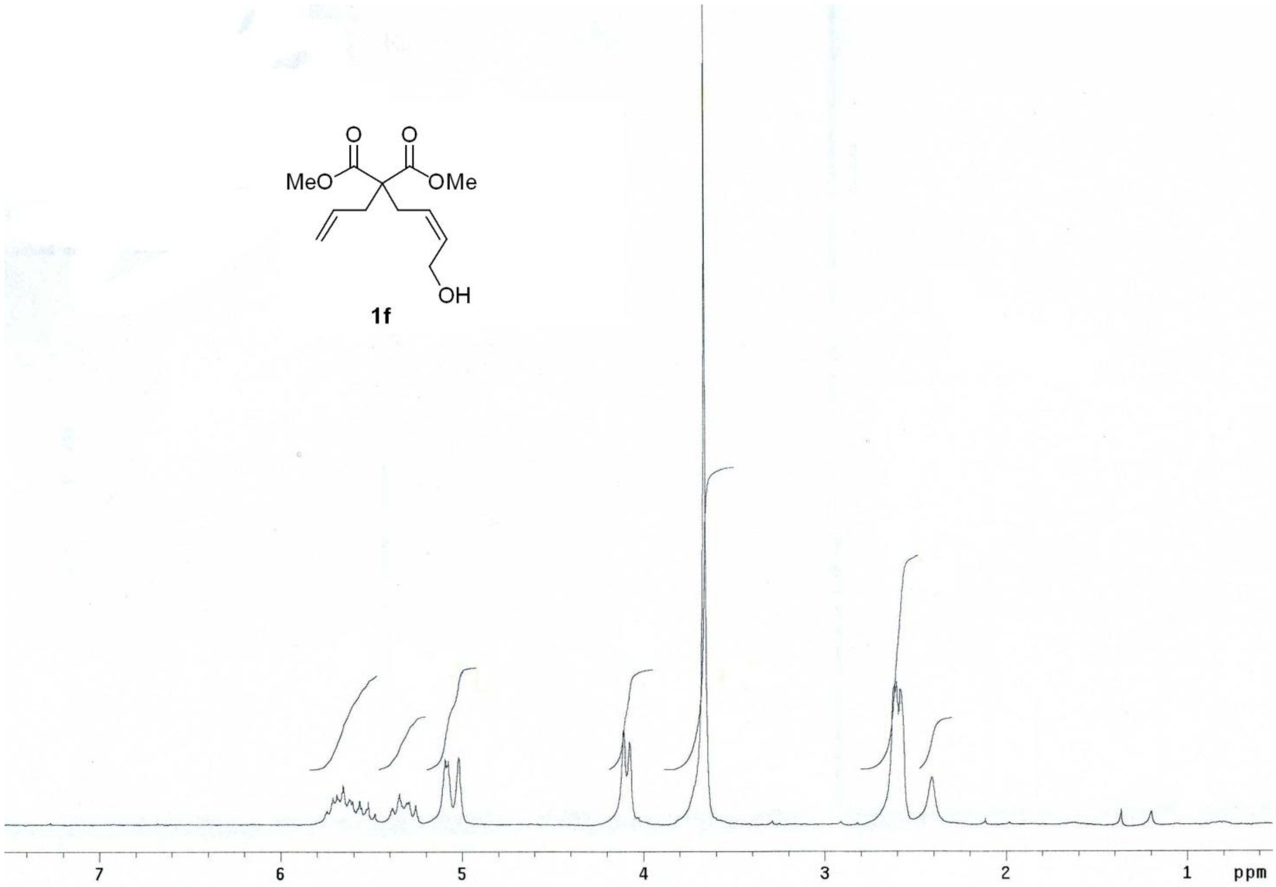
MeO
OMe
O
O
OH
1f
7
6
5
4
3
2
1
ppm

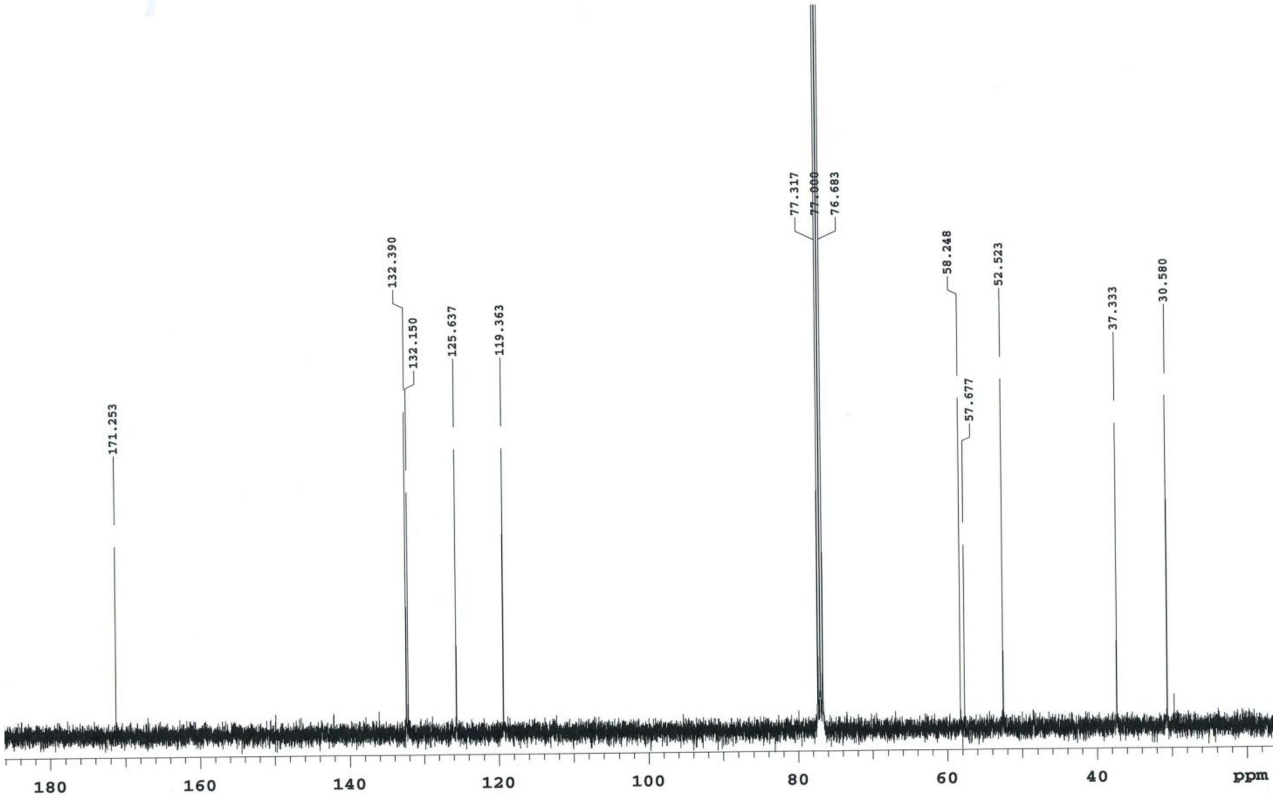
171.253
132.390
132.150
125.637
119.363
77.317
77.000
76.683
58.248
57.677
52.523
37.333
30.580
180
160
140
120
100
80
60
40
ppm

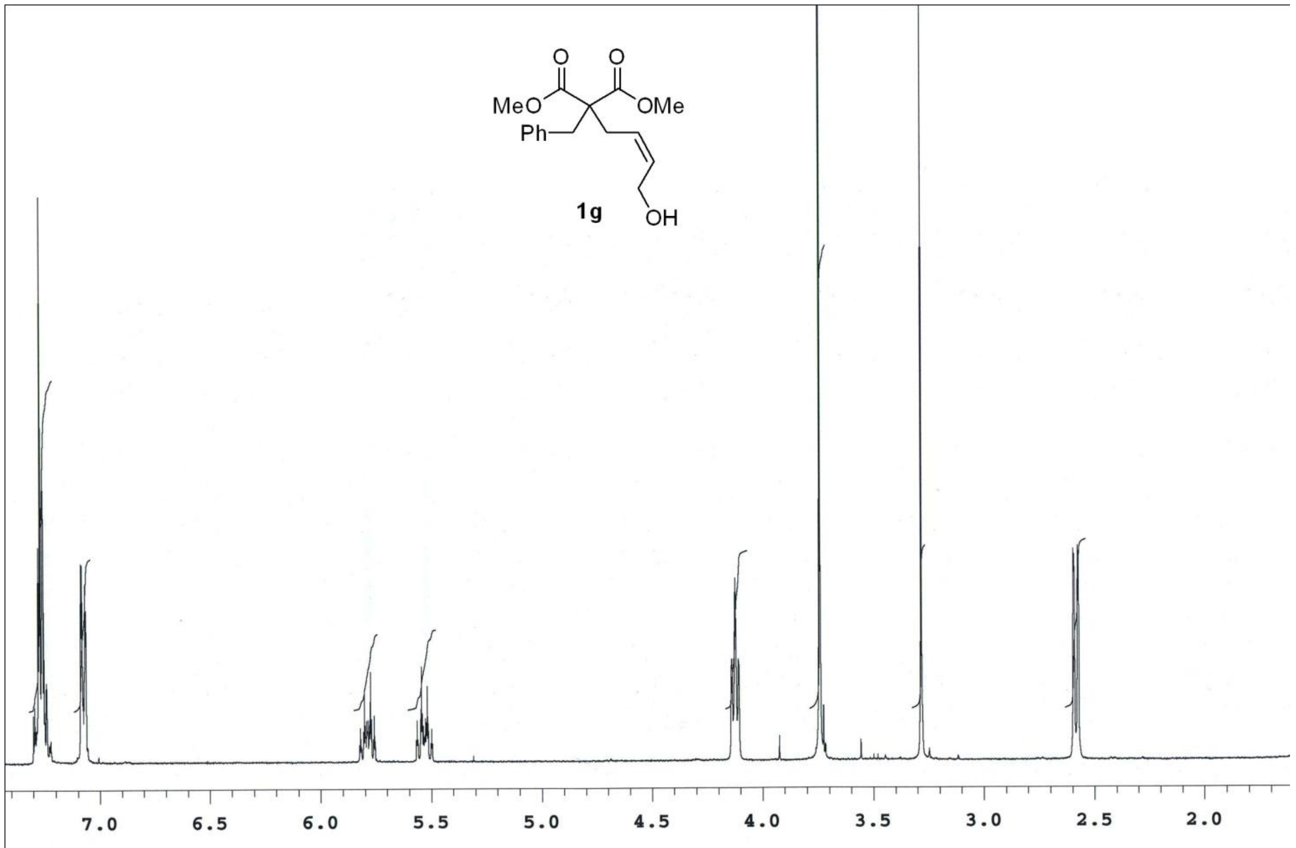
MeO
OMe
Ph
1g
OH
7.0
6.5
6.0
5.5
5.0
4.5
4.0
3.5
3.0
2.5
2.0

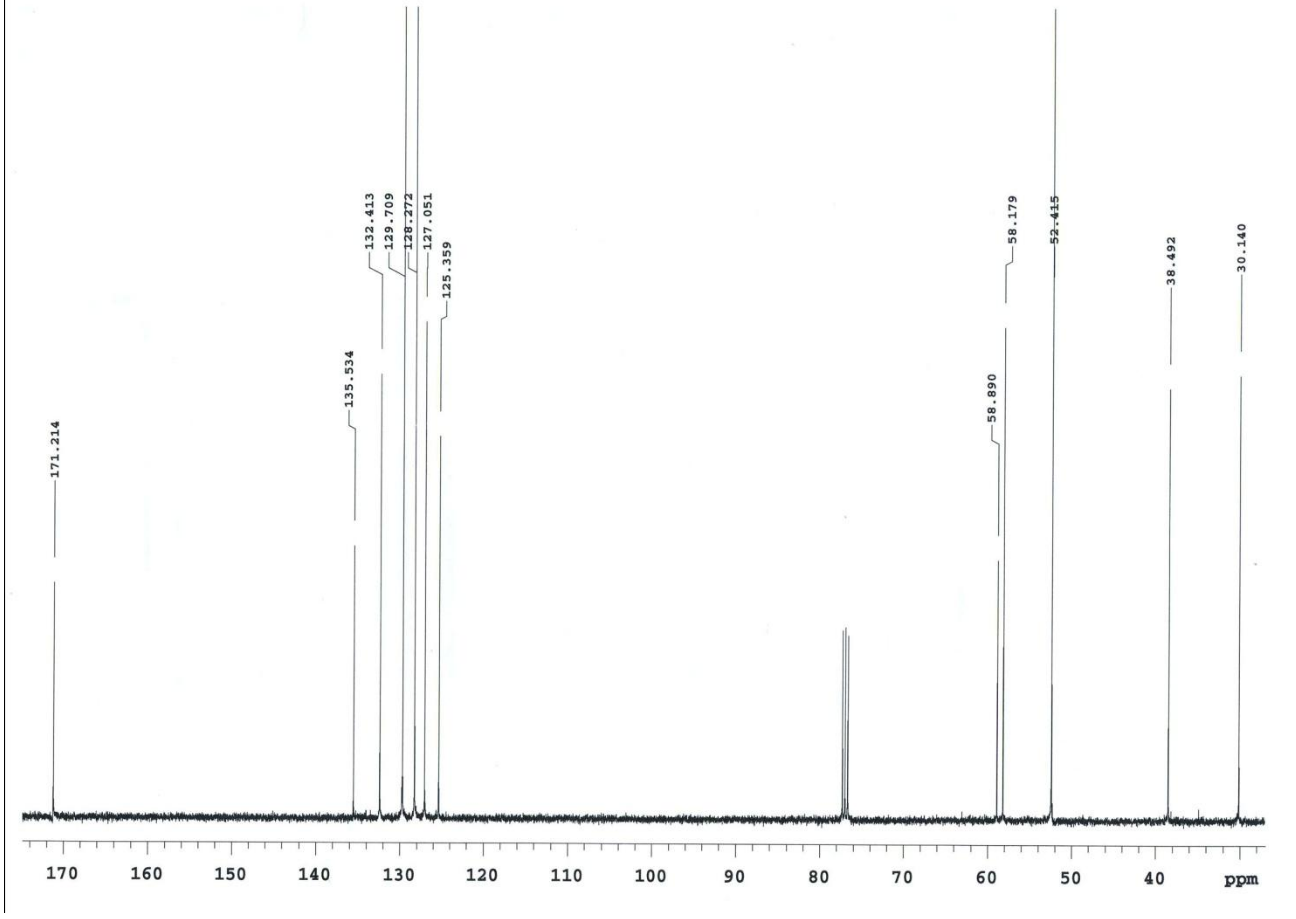
171.214
135.534
132.413
129.709
128.272
127.051
125.359
58.890
58.179
52.415
38.492
30.140
170
160
150
140
130
120
110
100
90
80
70
60
50
40
ppm

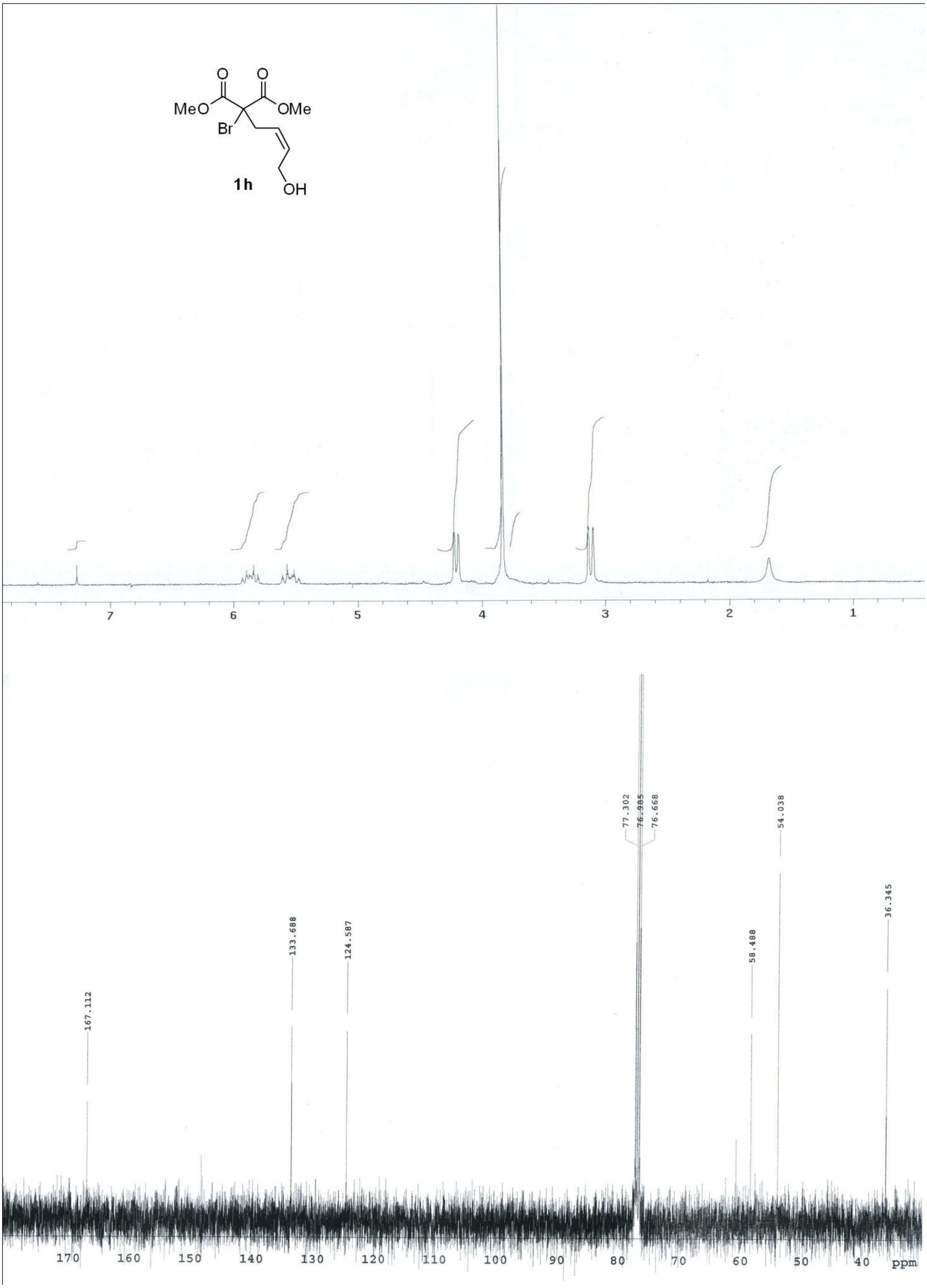
MeO
OMe
Br
OH
1h
7
6
5
4
3
2
1
167.112
133.688
124.587
77.302
76.985
76.668
58.488
54.038
36.345
170
160
150
140
130
120
110
100
90
80
70
60
50
40
ppm

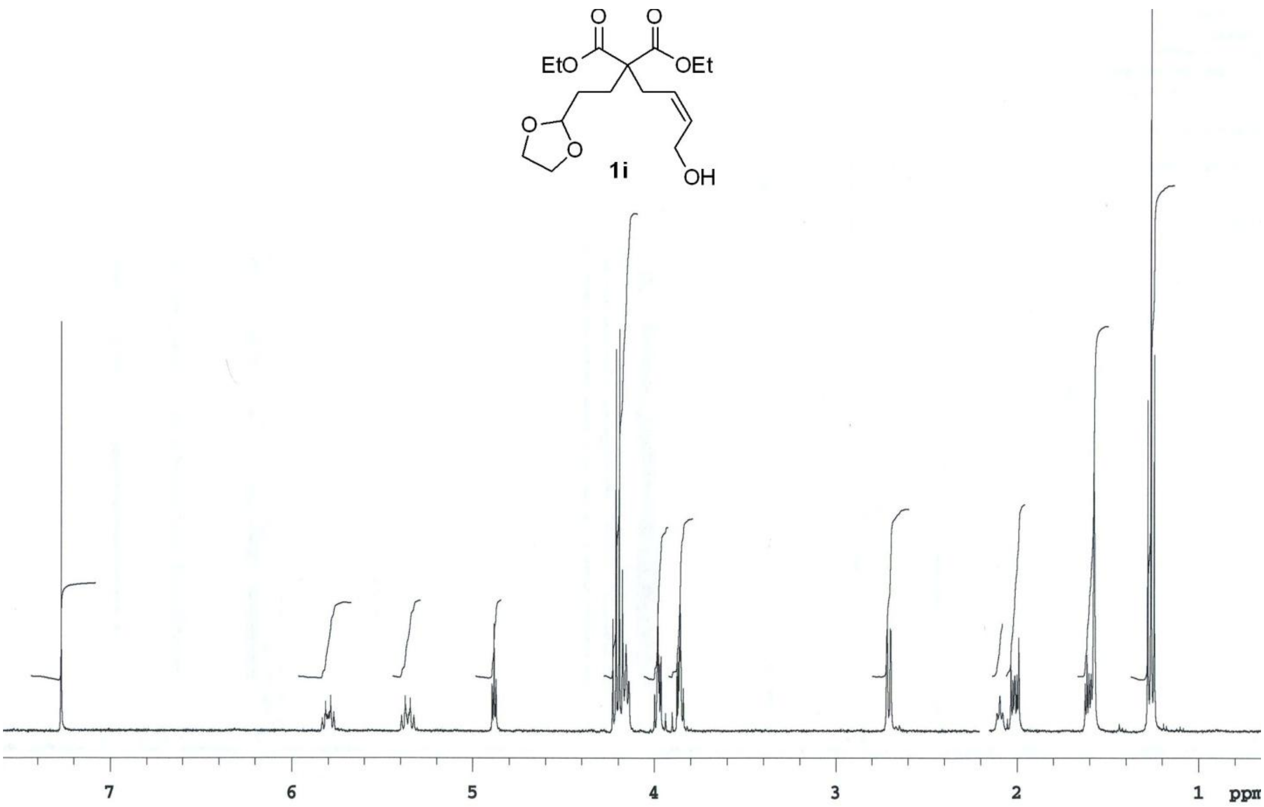

EtO
OEt
O
O
O
O
1i
OH
7
6
5
4
3
2
1
ppm

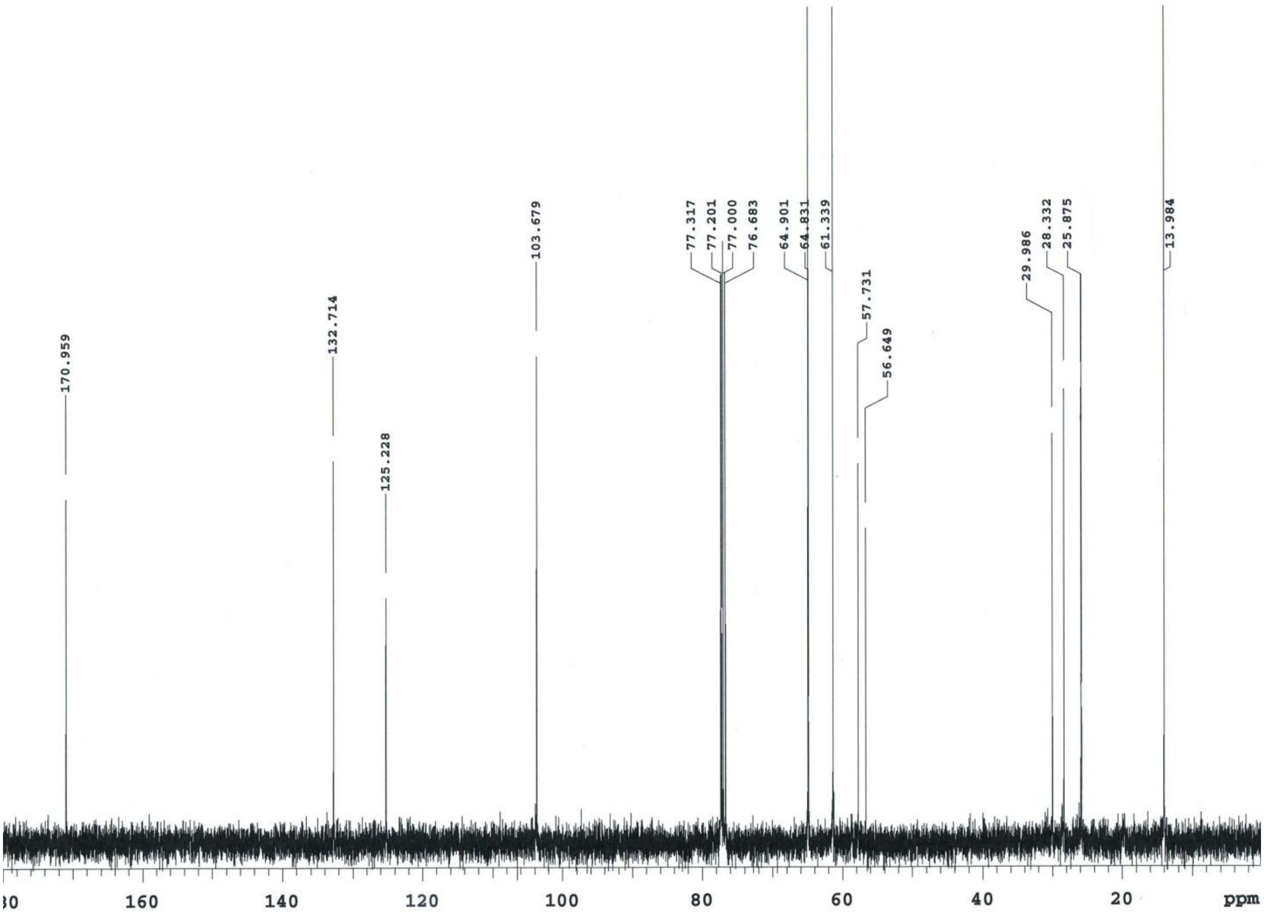

170.959
132.714
125.228
103.679
77.317
77.201
77.000
76.683
64.901
64.831
61.339
57.731
56.649
29.986
28.332
25.875
13.984
80
160
140
120
100
80
60
40
20
ppm

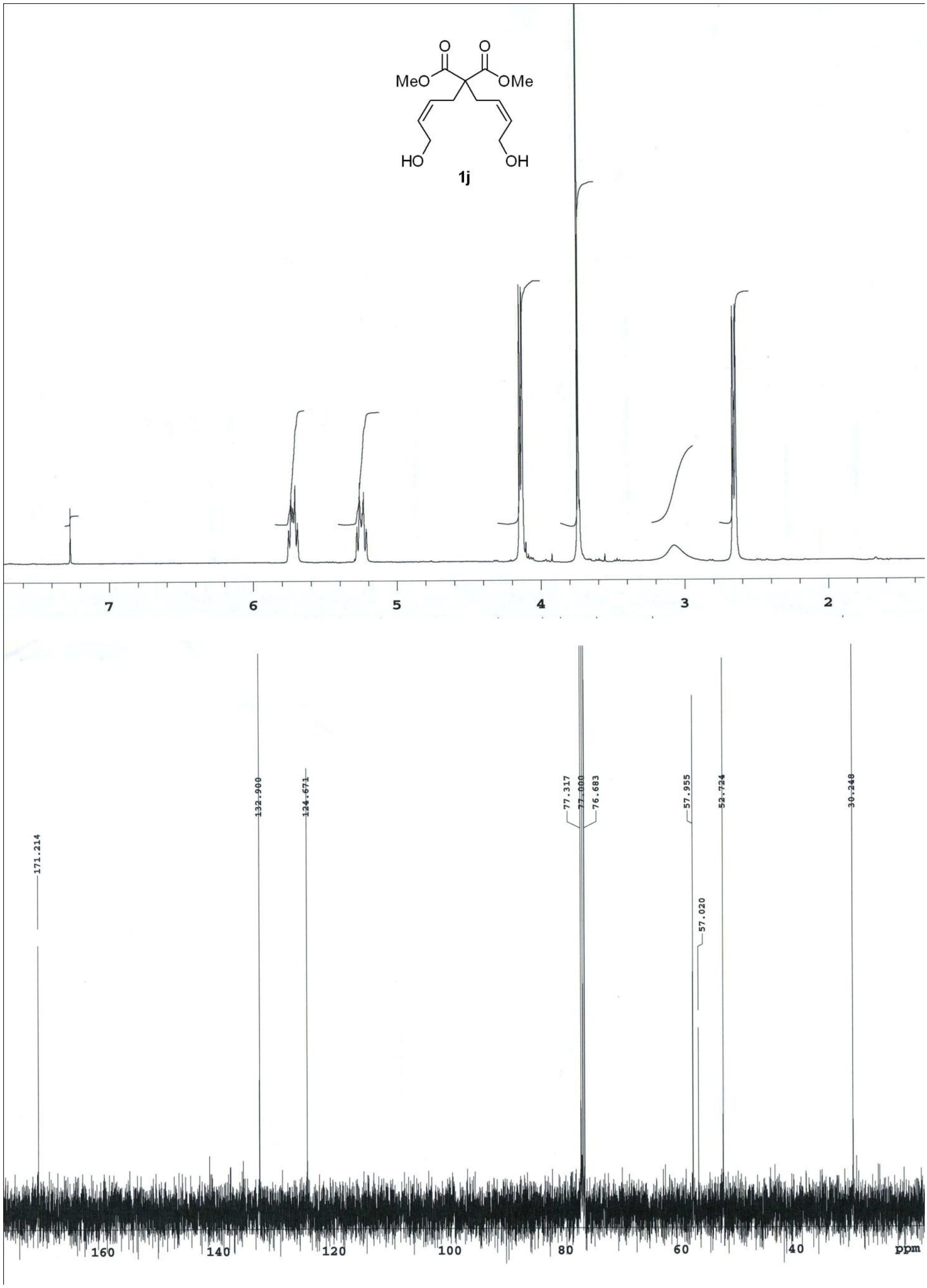
MeO
OMe
HO
OH
1j
171.214
132.900
124.671
77.317
77.000
76.683
57.955
57.020
52.724
30.248
ppm

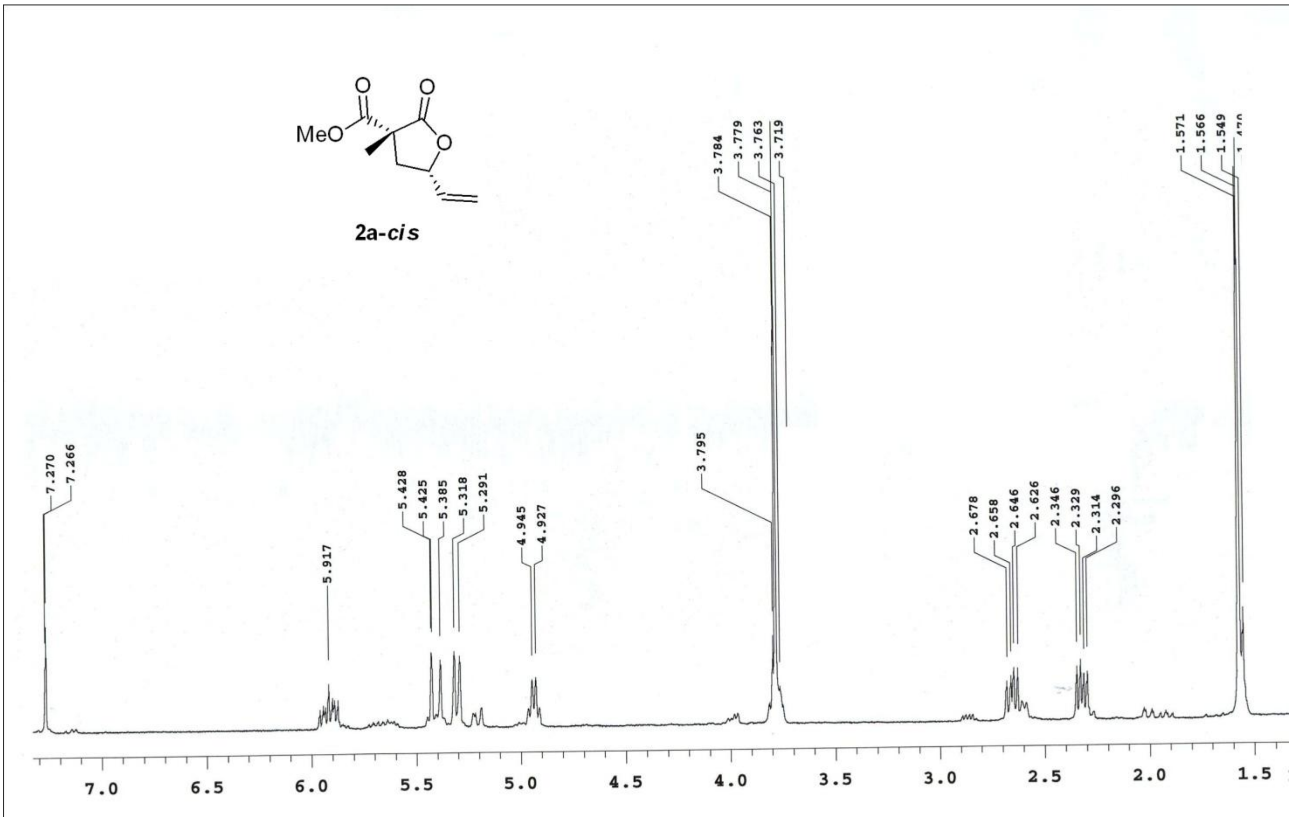
MeO
2a-cis
7.270
7.266
5.917
5.428
5.425
5.385
5.318
5.291
4.945
4.927
3.795
3.784
3.779
3.763
3.719
2.678
2.658
2.646
2.626
2.346
2.329
2.314
2.296
1.571
1.566
1.549
7.0
6.5
6.0
5.5
5.0
4.5
4.0
3.5
3.0
2.5
2.0
1.5

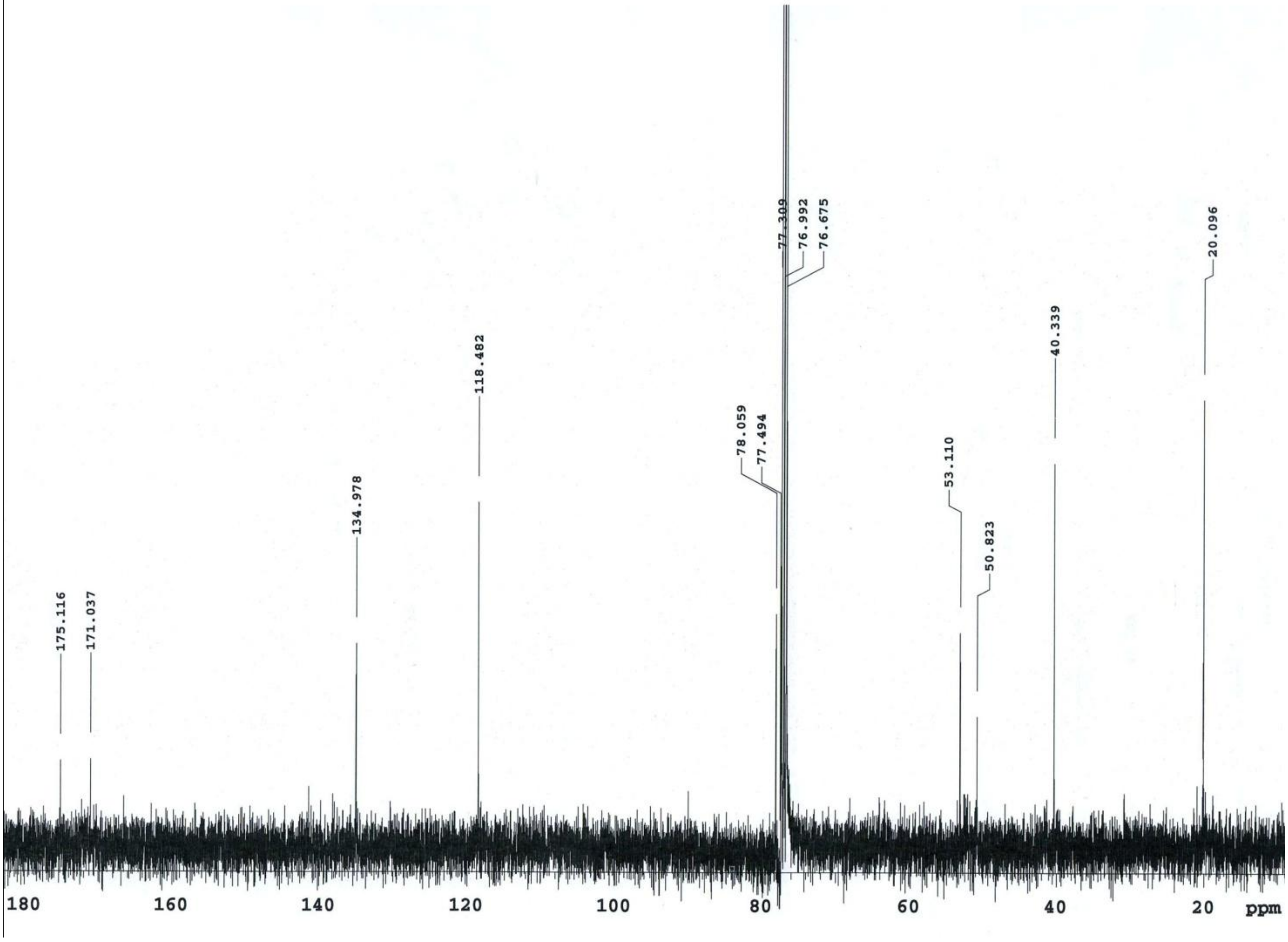
175.116
171.037
134.978
118.482
78.059
77.494
77.309
76.992
76.675
53.110
50.823
40.339
20.096
180
160
140
120
100
80
60
40
20
ppm

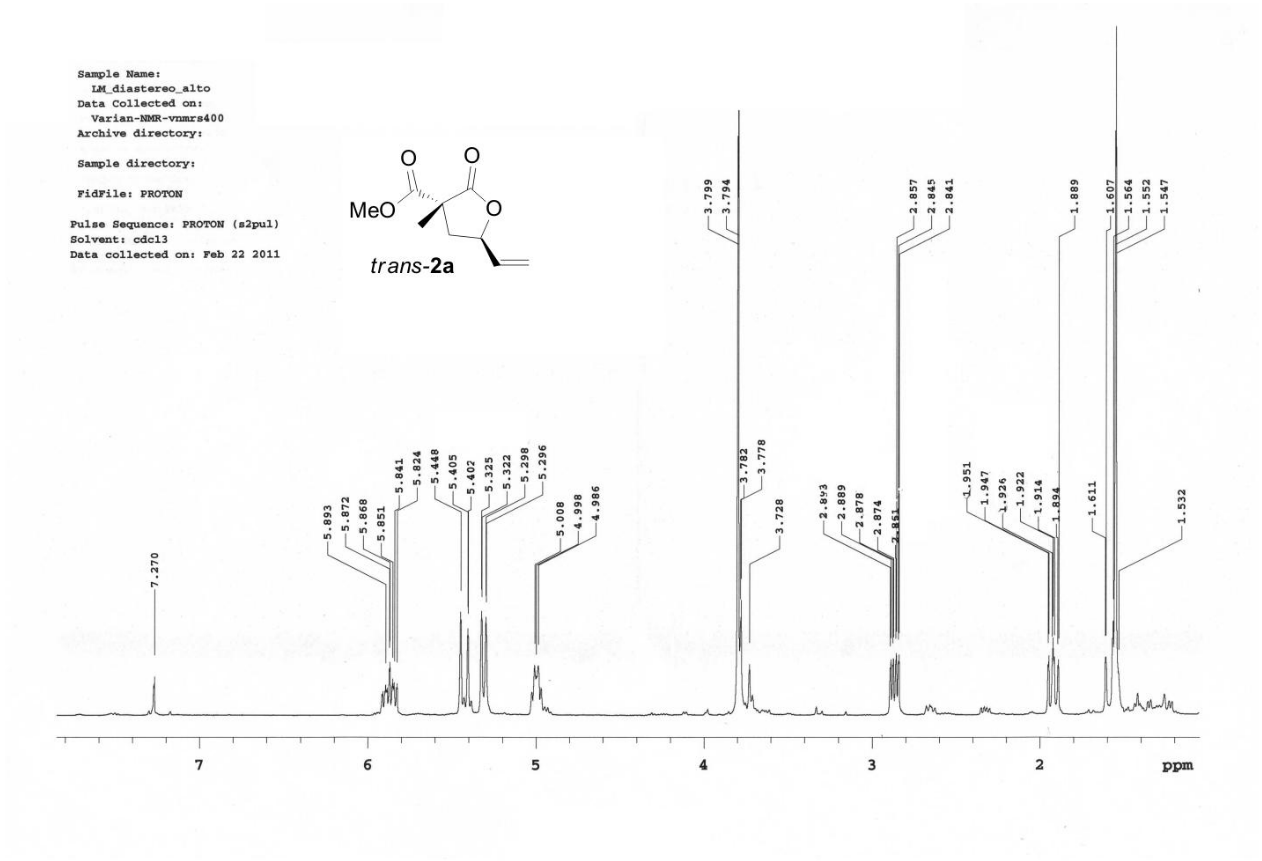
Sample Name:
LM_diastereo_alto
Data Collected on:
Varian-NMR-vnmrs400
Archive directory:
Sample directory:
FidFile: PROTON
Pulse Sequence: PROTON (s2pul)
Solvent: cdcl3
Data collected on: Feb 22 2011
MeO
O
O
O
trans-2a
3.799
3.794
2.857
2.845
2.841
1.889
1.607
1.564
1.552
1.547
7.270
5.893
5.872
5.868
5.851
5.841
5.824
5.448
5.405
5.402
5.325
5.322
5.298
5.296
5.008
4.998
4.986
3.782
3.778
3.728
2.893
2.889
2.878
2.874
2.861
1.951
1.947
1.926
1.922
1.914
1.894
1.611
1.532
7
6
5
4
3
2
ppm

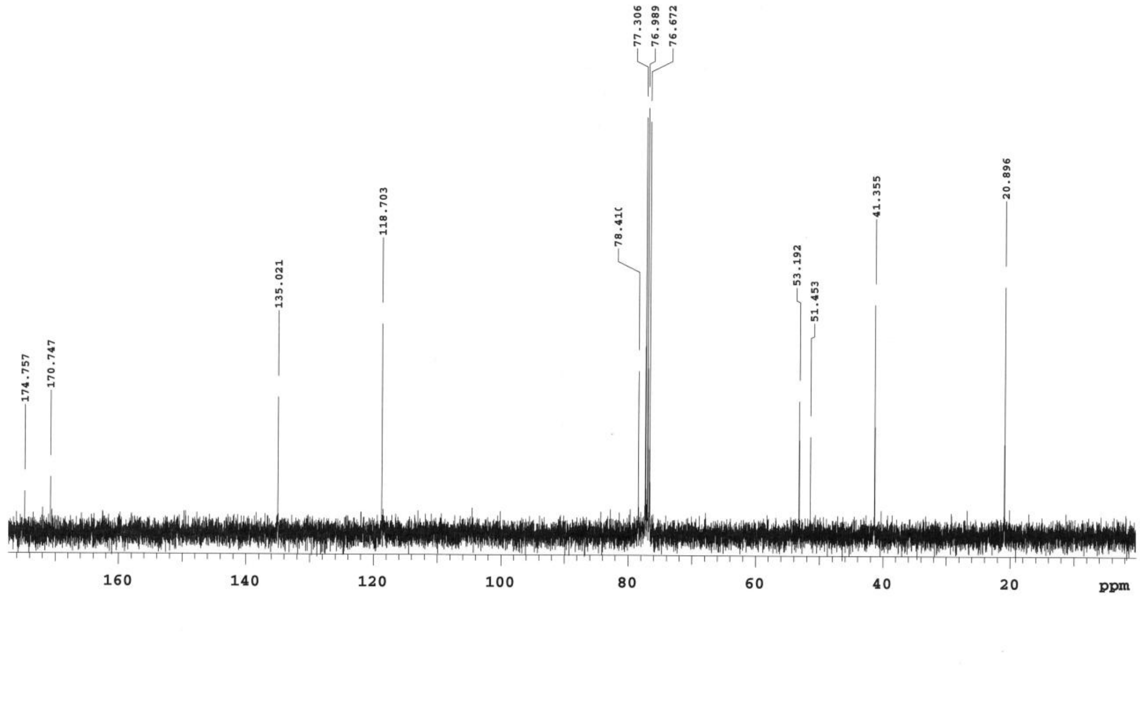
77.306
76.989
76.672
78.410
174.757
170.747
135.021
118.703
53.192
51.453
41.355
20.896
160
140
120
100
80
60
40
20
ppm

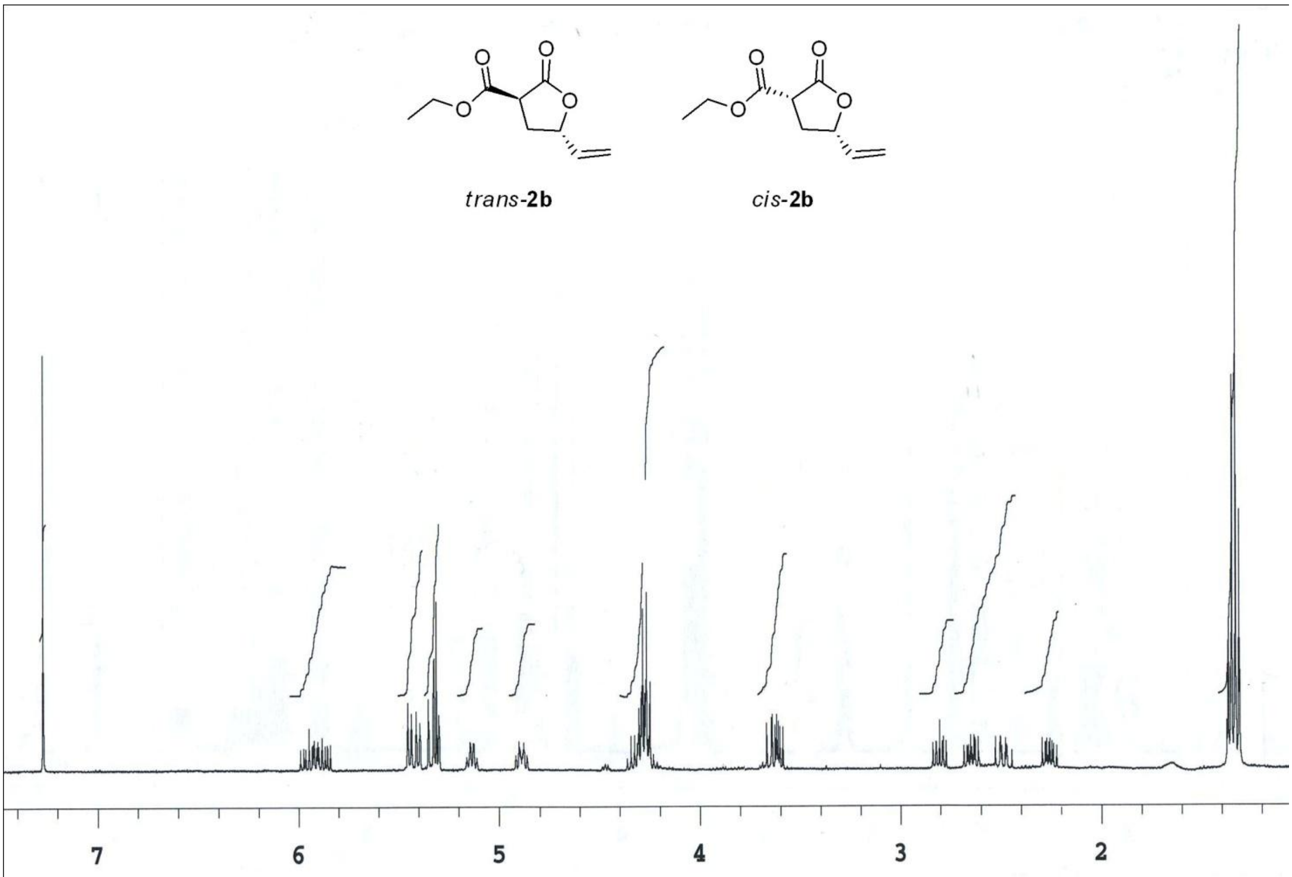
trans-2b
cis-2b
7
6
5
4
3
2

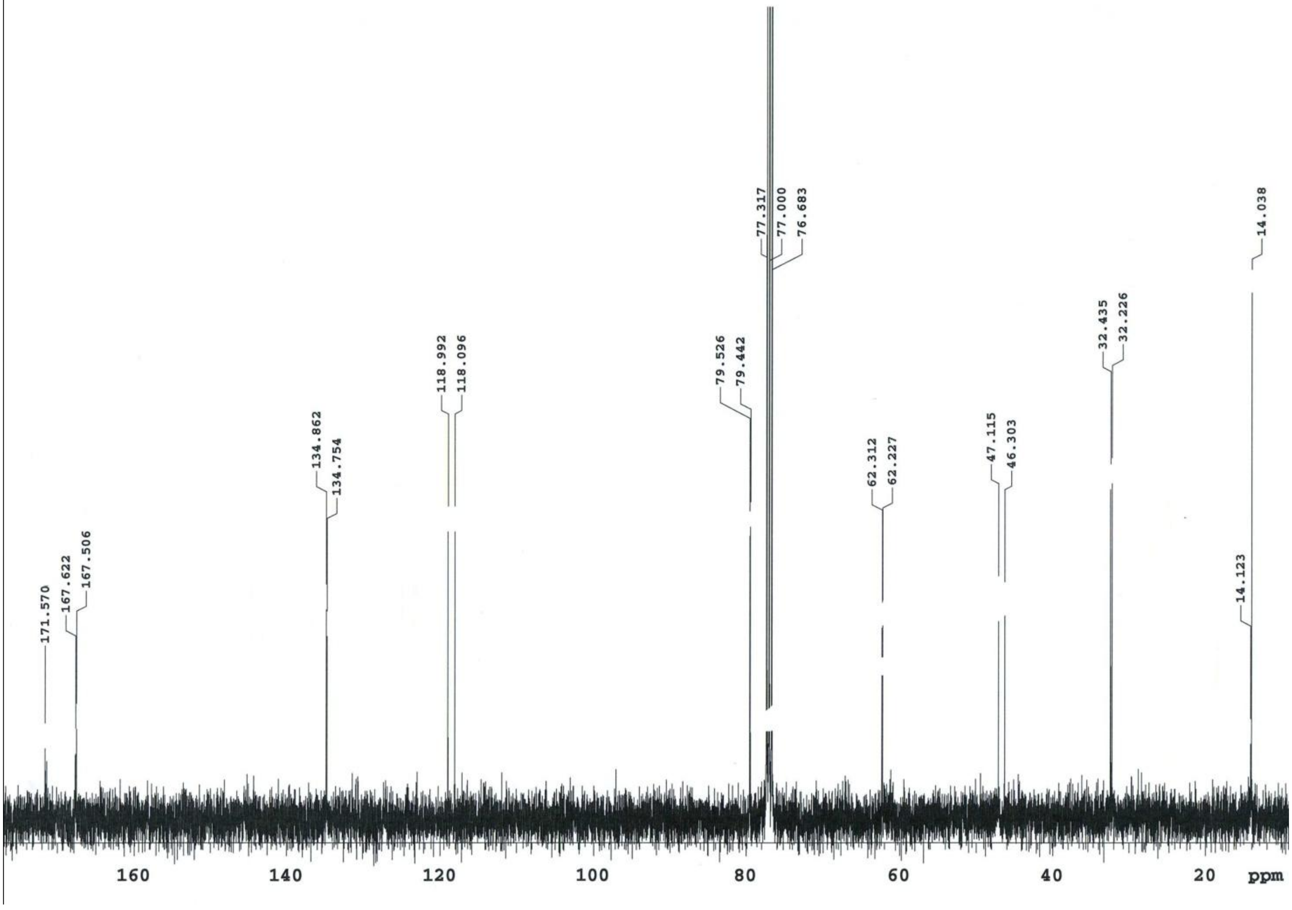
171.570
167.622
167.506
134.862
134.754
118.992
118.096
79.526
79.442
77.317
77.000
76.683
62.312
62.227
47.115
46.303
32.435
32.226
14.038
14.123
160
140
120
100
80
60
40
20
ppm

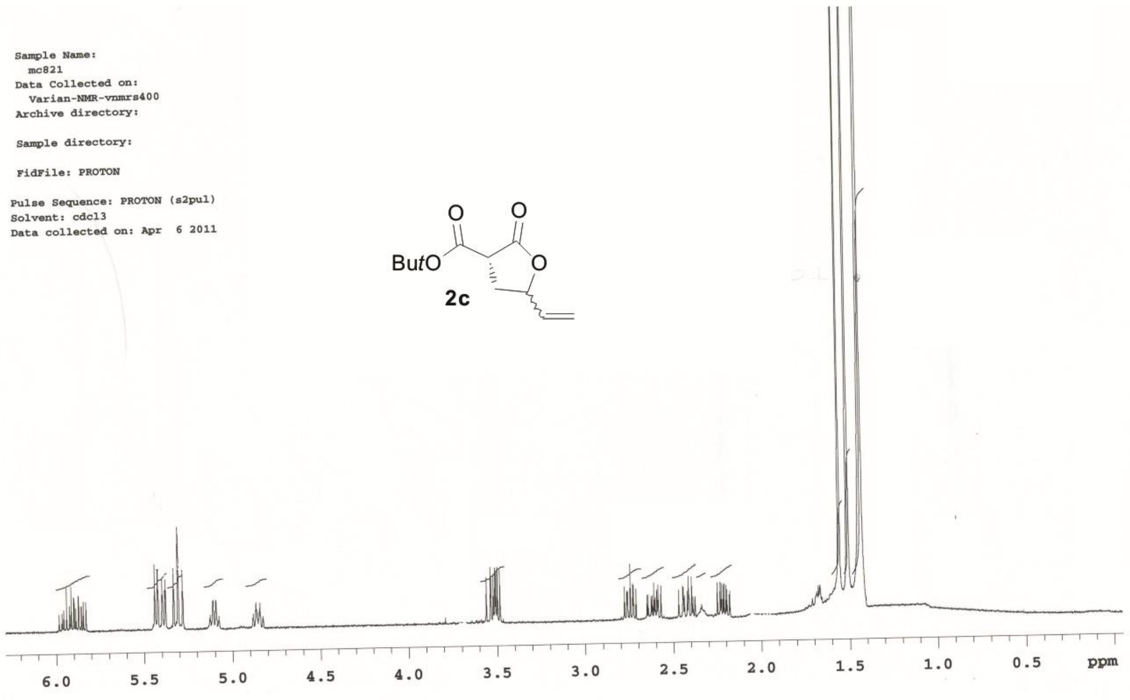
Sample Name:
mc821
Data Collected on:
Varian-NMR-vnmrs400
Archive directory:
Sample directory:
FidFile: PROTON
Pulse Sequence: PROTON (s2pul)
Solvent: cdcl3
Data collected on: Apr 6 2011
ButO
2c
6.0
5.5
5.0
4.5
4.0
3.5
3.0
2.5
2.0
1.5
1.0
0.5
ppm

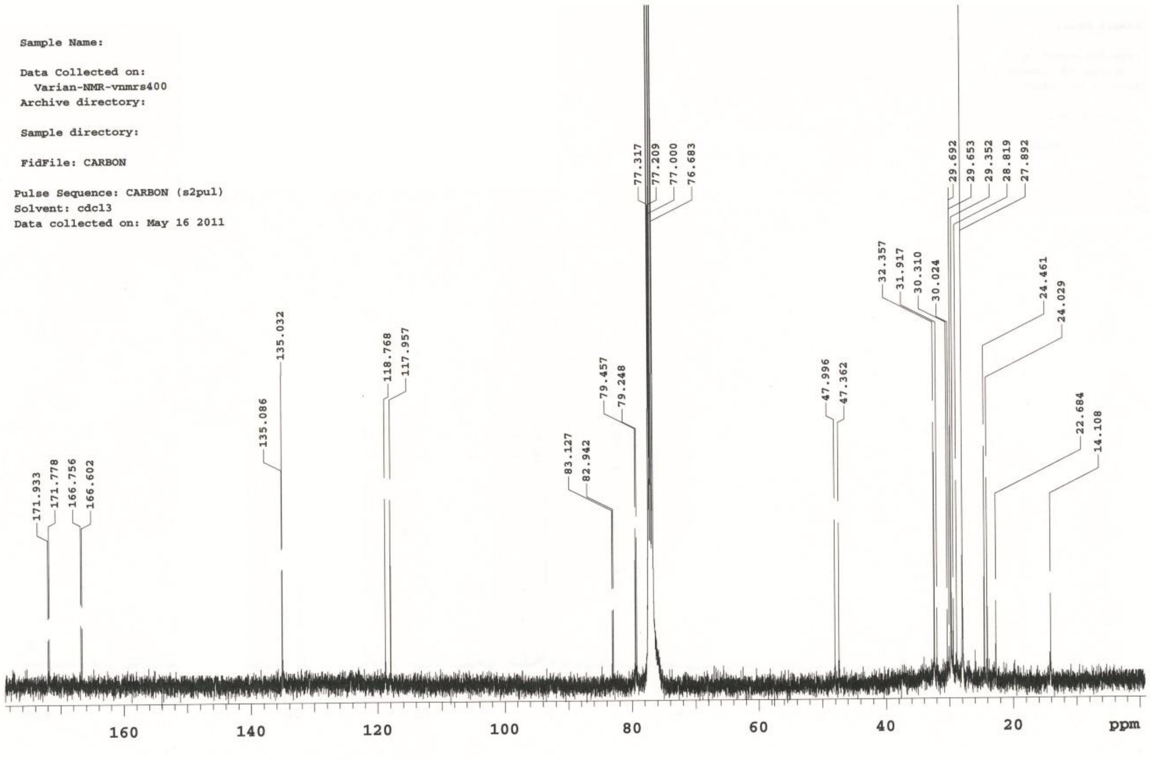
Sample Name:
Data Collected on:
Varian-NMR-vnmrs400
Archive directory:
Sample directory:
FidFile: CARBON
Pulse Sequence: CARBON (s2pul)
Solvent: cdcl3
Data collected on: May 16 2011
171.933
171.778
166.756
166.602
135.086
135.032
118.768
117.957
83.127
82.942
79.457
79.248
77.317
77.209
77.000
76.683
47.996
47.362
32.357
31.917
30.310
30.024
29.692
29.653
29.352
28.819
27.892
24.461
24.029
22.684
14.108
160
140
120
100
80
60
40
20
ppm

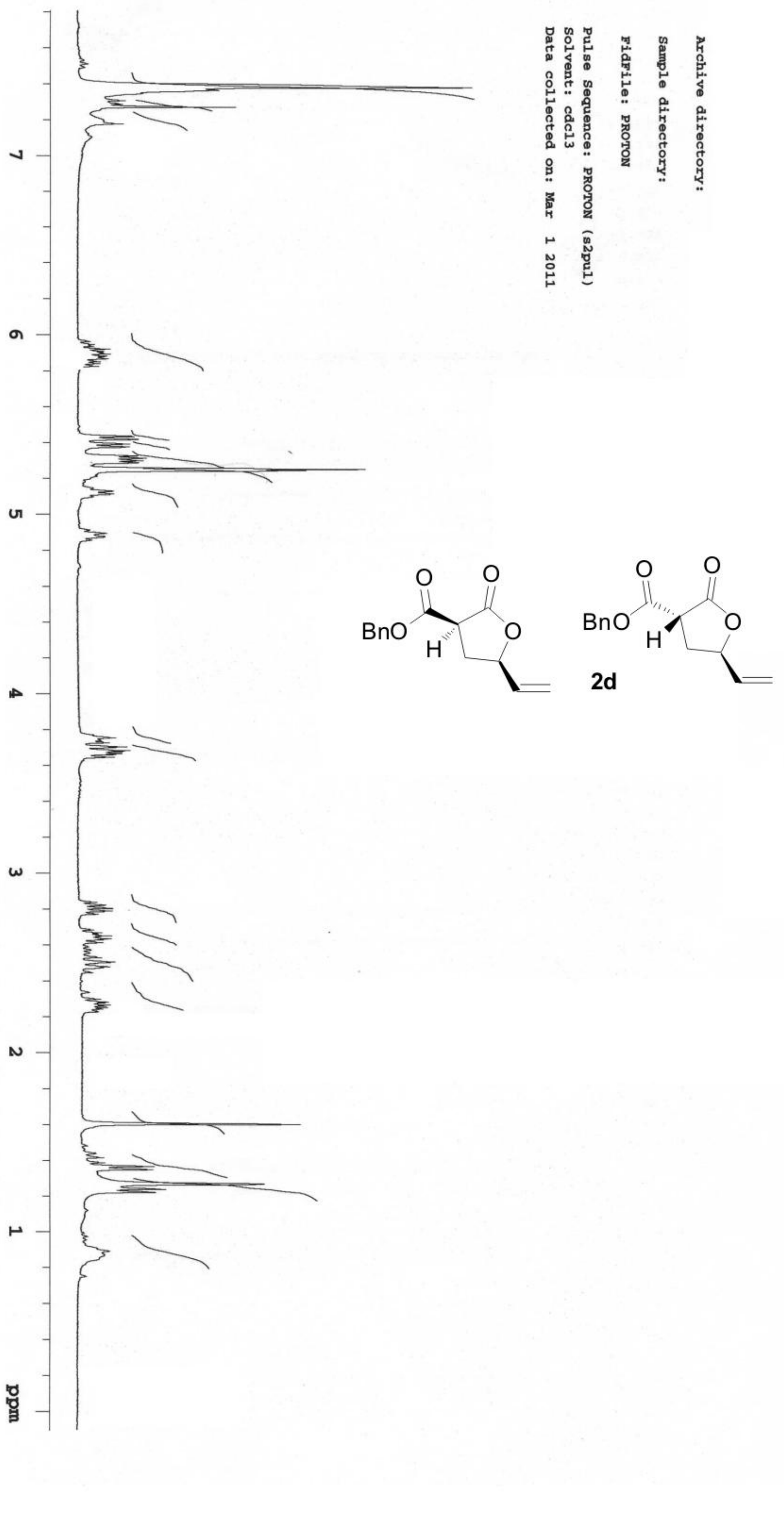
Archive directory:
Sample directory:
FidFile: PROTON
Pulse Sequence: PROTON (s2pul)
Solvent: cdcl3
Data collected on: Mar 1 2011
ppm
7
6
5
4
3
2
1
BnO
H
O
2d

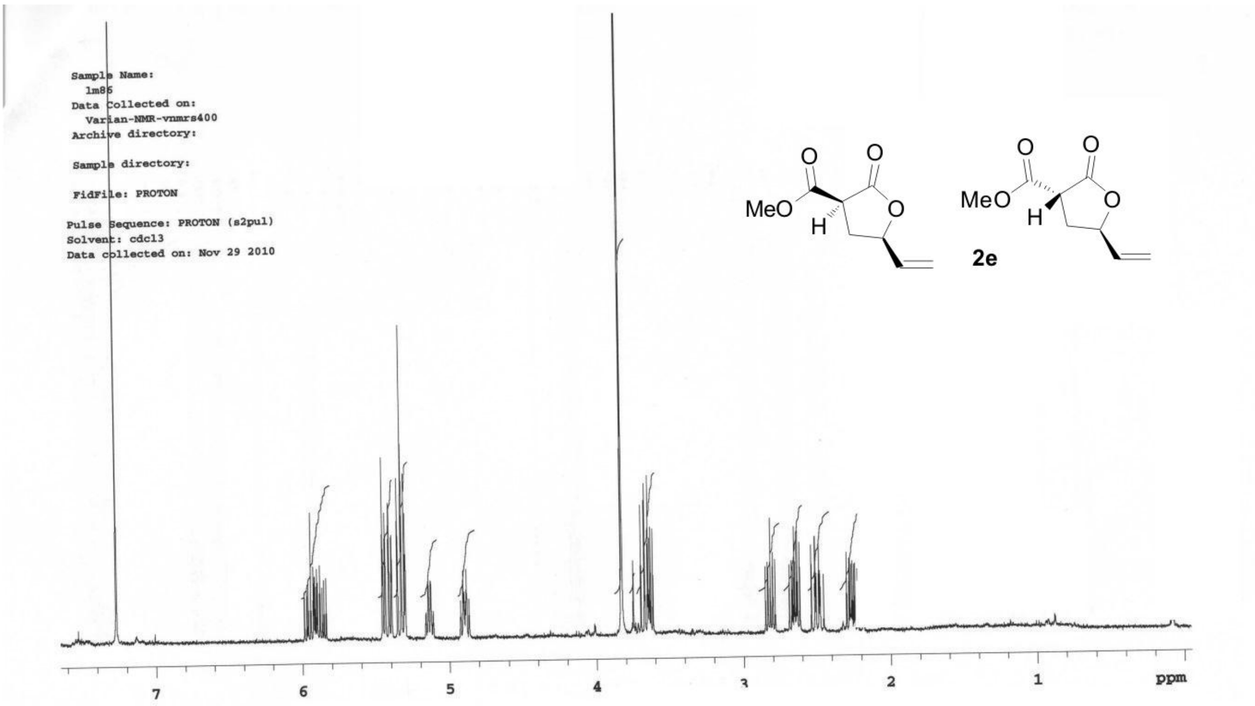
Sample Name:
lm86
Data Collected on:
Varian-NMR-vnmrs400
Archive directory:
Sample directory:
FidFile: PROTON
Pulse Sequence: PROTON (s2pul)
Solvent: cdcl3
Data collected on: Nov 29 2010
MeO
O
O
H
O
2e
7
6
5
4
3
2
1
ppm

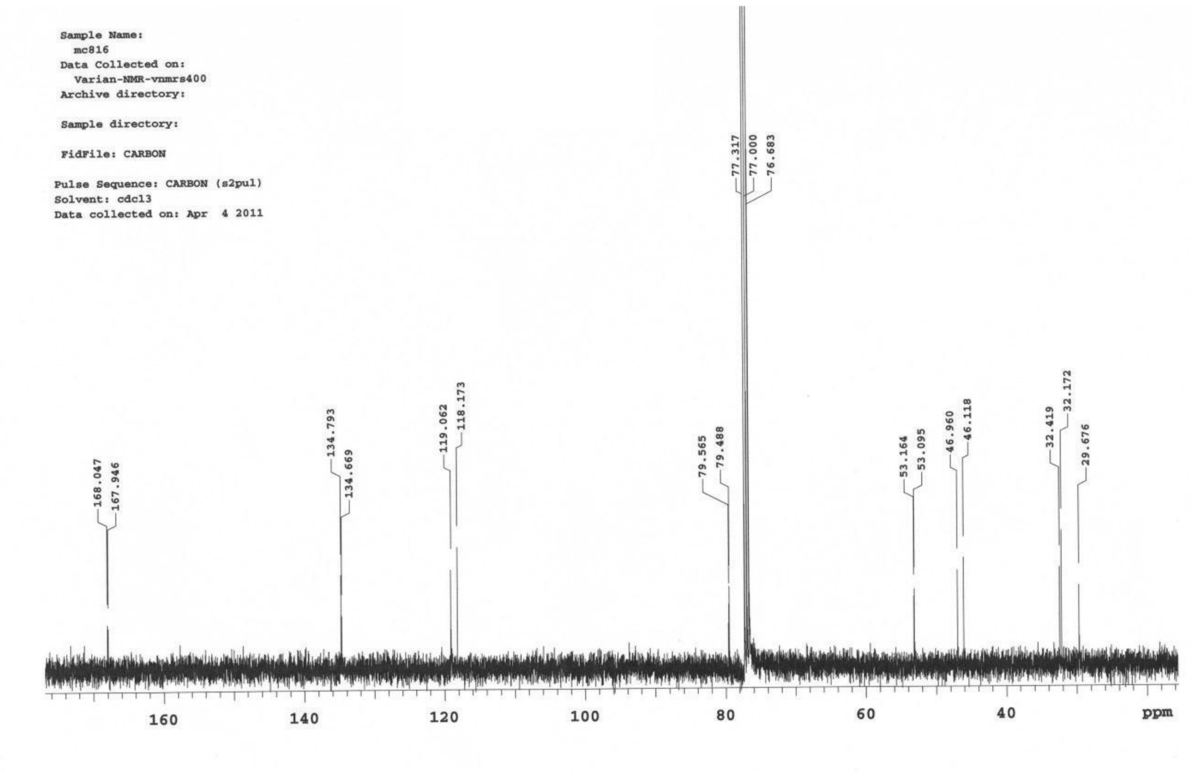
Sample Name:
mc816
Data Collected on:
Varian-NMR-vnmrs400
Archive directory:
Sample directory:
FidFile: CARBON
Pulse Sequence: CARBON (s2pul)
Solvent: cdcl3
Data collected on: Apr 4 2011
168.047
167.946
134.793
134.669
119.062
118.173
79.565
79.488
77.317
77.000
76.683
53.164
53.095
46.960
46.118
32.419
32.172
29.676
160
140
120
100
80
60
40
ppm

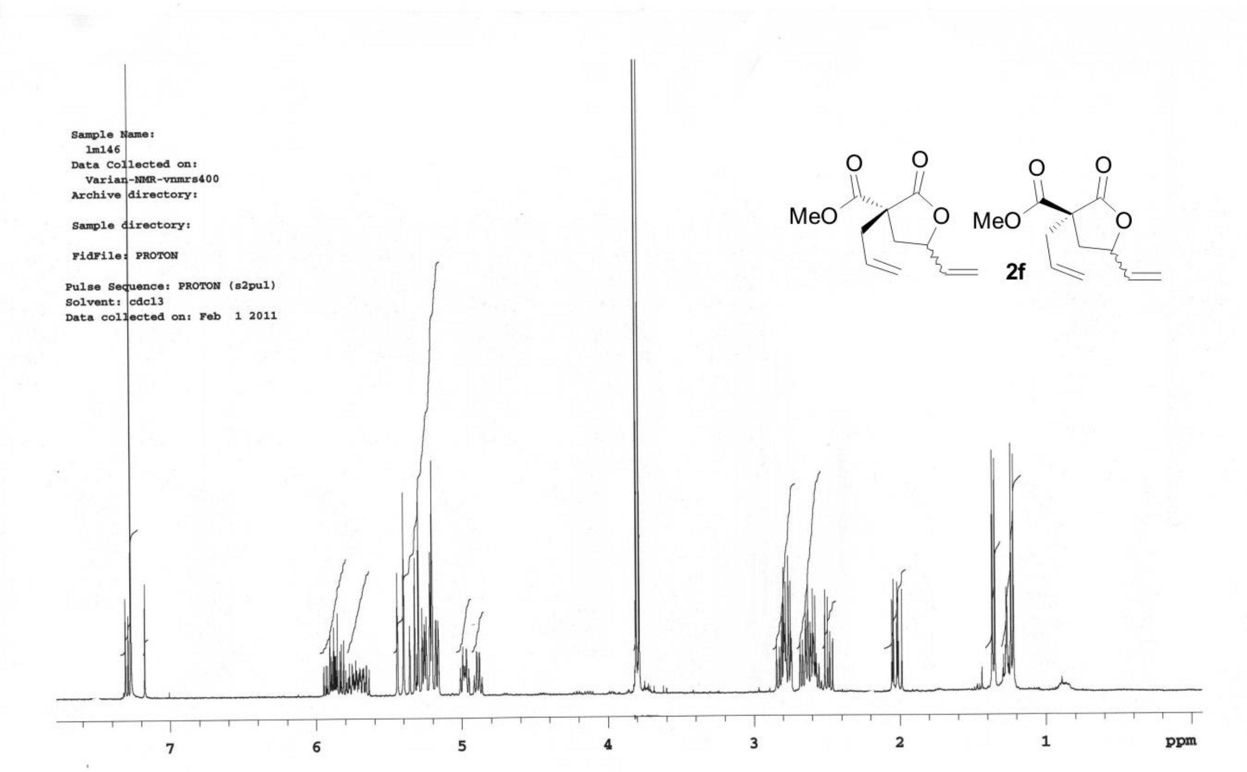

Sample Name:
1m146
Data Collected on:
Varian-NMR-vnmrs400
Archive directory:
Sample directory:
FidFile: PROTON
Pulse Sequence: PROTON (s2pul)
Solvent: cdcl3
Data collected on: Feb 1 2011
MeO
O
2f
7
6
5
4
3
2
1
ppm

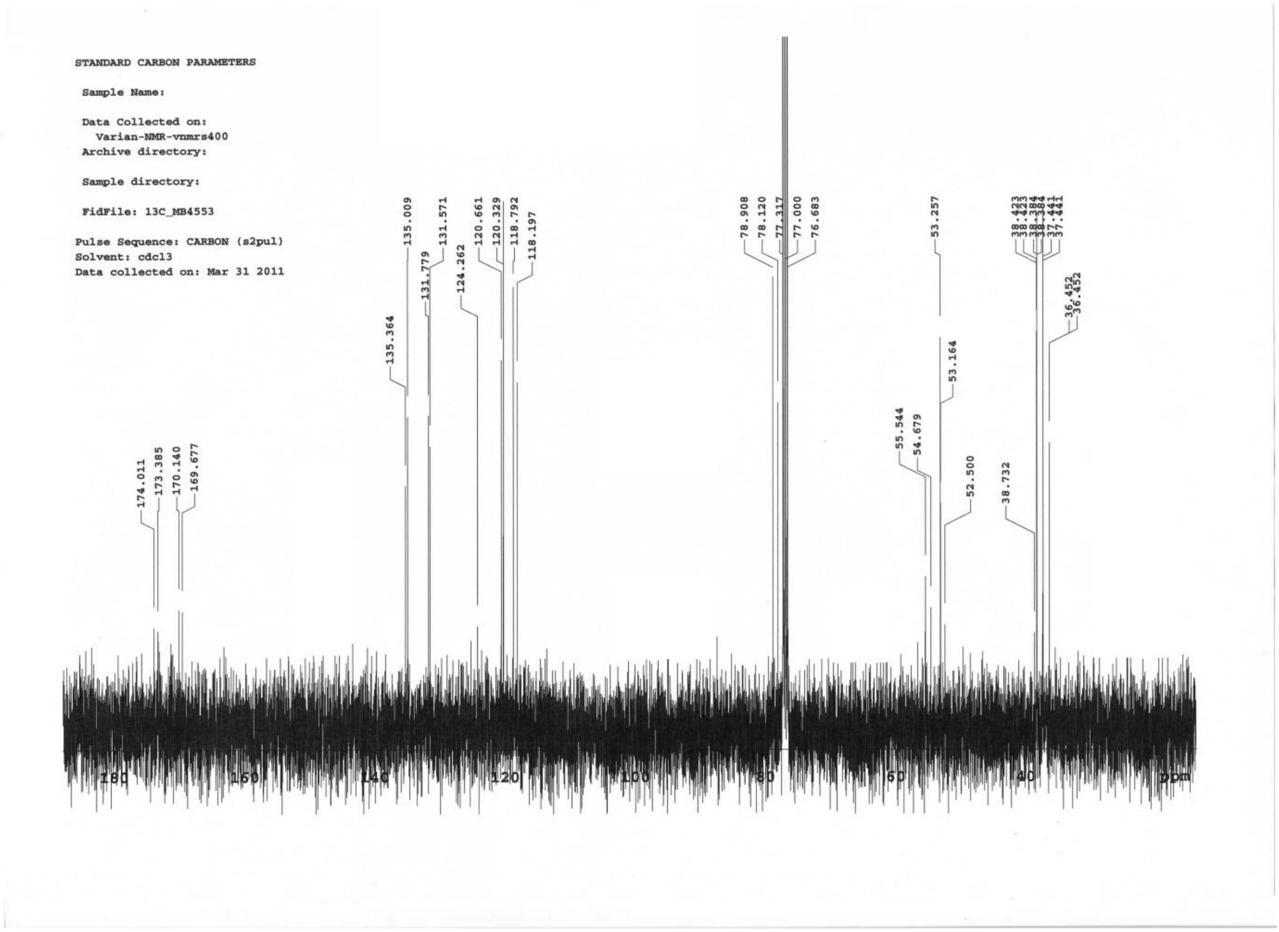

STANDARD CARBON PARAMETERS
Sample Name:
Data Collected on:
Varian-NMR-vnmrs400
Archive directory:
Sample directory:
FidFile: 13C_MB4553
Pulse Sequence: CARBON (s2pul)
Solvent: cdcl3
Data collected on: Mar 31 2011
174.011
173.385
170.140
169.677
135.364
135.009
131.779
131.571
124.262
120.661
120.329
118.792
118.197
78.908
78.120
77.317
77.000
76.683
55.544
54.679
53.257
53.164
52.500
38.732
38.423
38.423
38.384
38.384
37.441
37.441
36.452
36.452
180
160
140
120
100
80
60
40
ppm

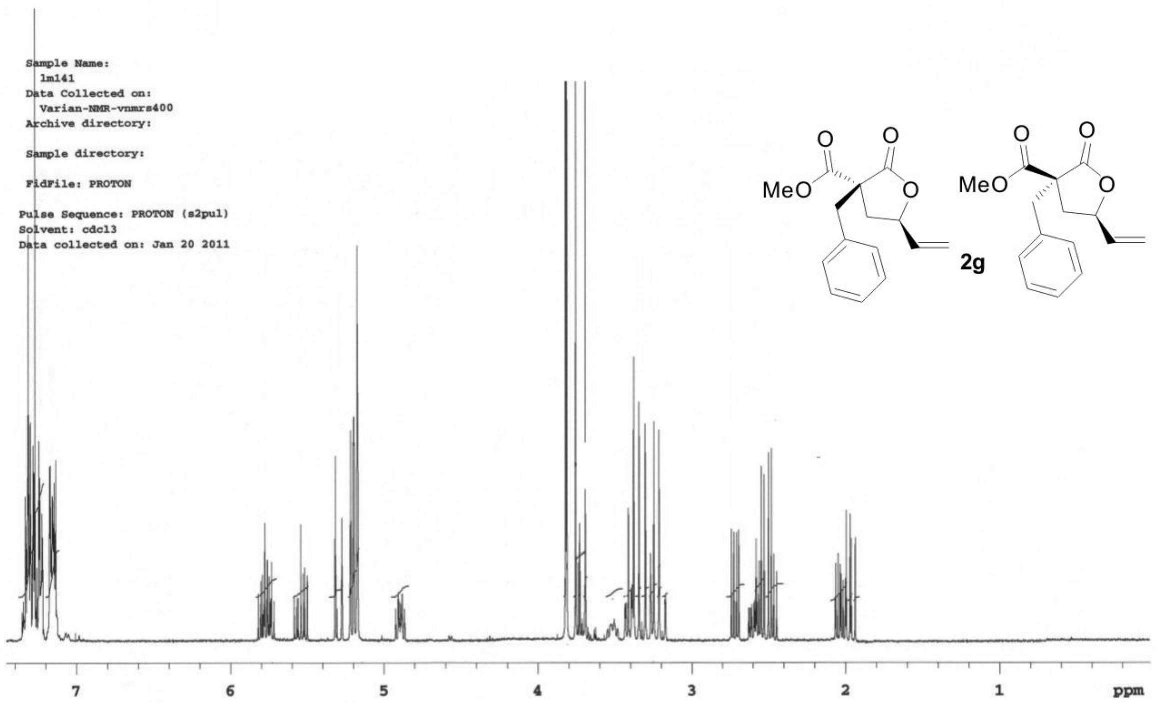
Sample Name:
lm141
Data Collected on:
Varian-NMR-vnmrs400
Archive directory:
Sample directory:
FidFile: PROTON
Pulse Sequence: PROTON (s2pul)
Solvent: cdcl3
Data collected on: Jan 20 2011
MeO
O
O
O
2g
7
6
5
4
3
2
1
ppm

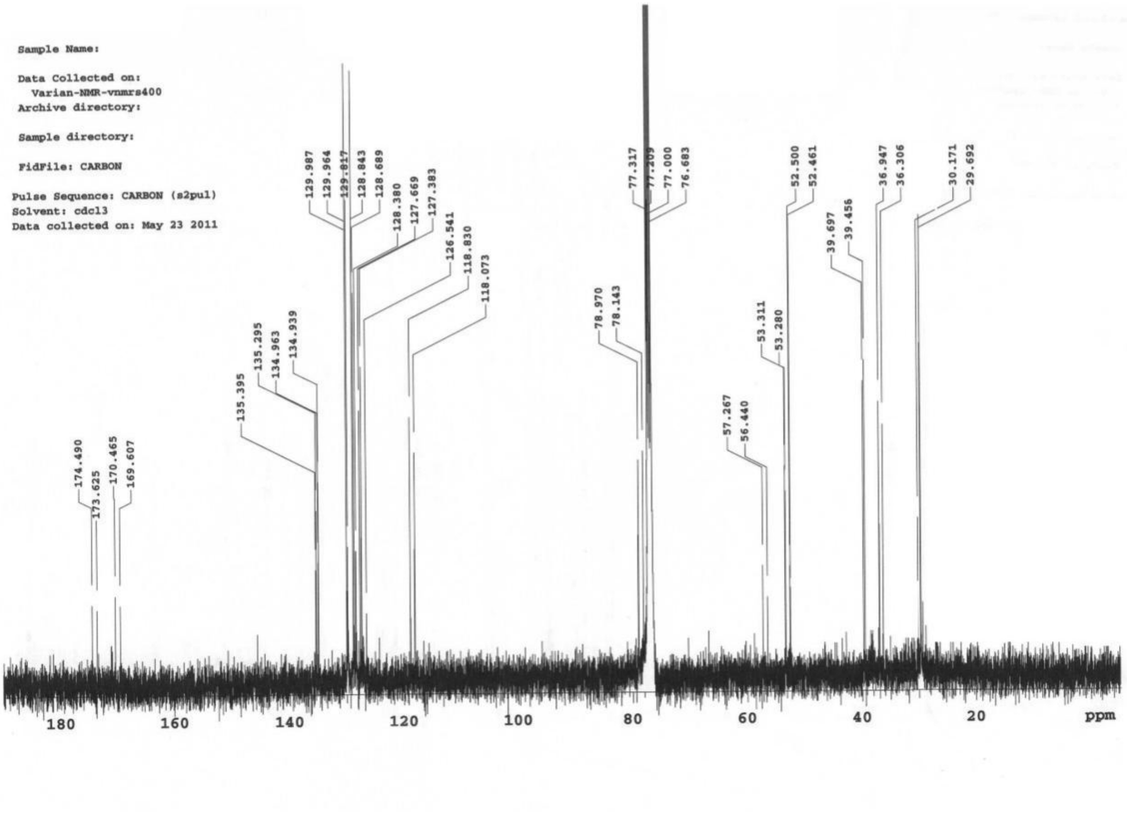
Sample Name:
Data Collected on:
Varian-NMR-vnmrs400
Archive directory:
Sample directory:
FidFile: CARBON
Pulse Sequence: CARBON (s2pul)
Solvent: cdcl3
Data collected on: May 23 2011
174.490
173.625
170.465
169.607
135.395
135.295
134.963
134.939
129.987
129.964
129.817
128.843
128.689
128.380
127.669
127.383
126.541
118.830
118.073
78.970
78.143
77.317
77.209
77.000
76.683
57.267
56.440
53.311
53.280
52.500
52.461
39.697
39.456
36.947
36.306
30.171
29.692
180
160
140
120
100
80
60
40
20
ppm

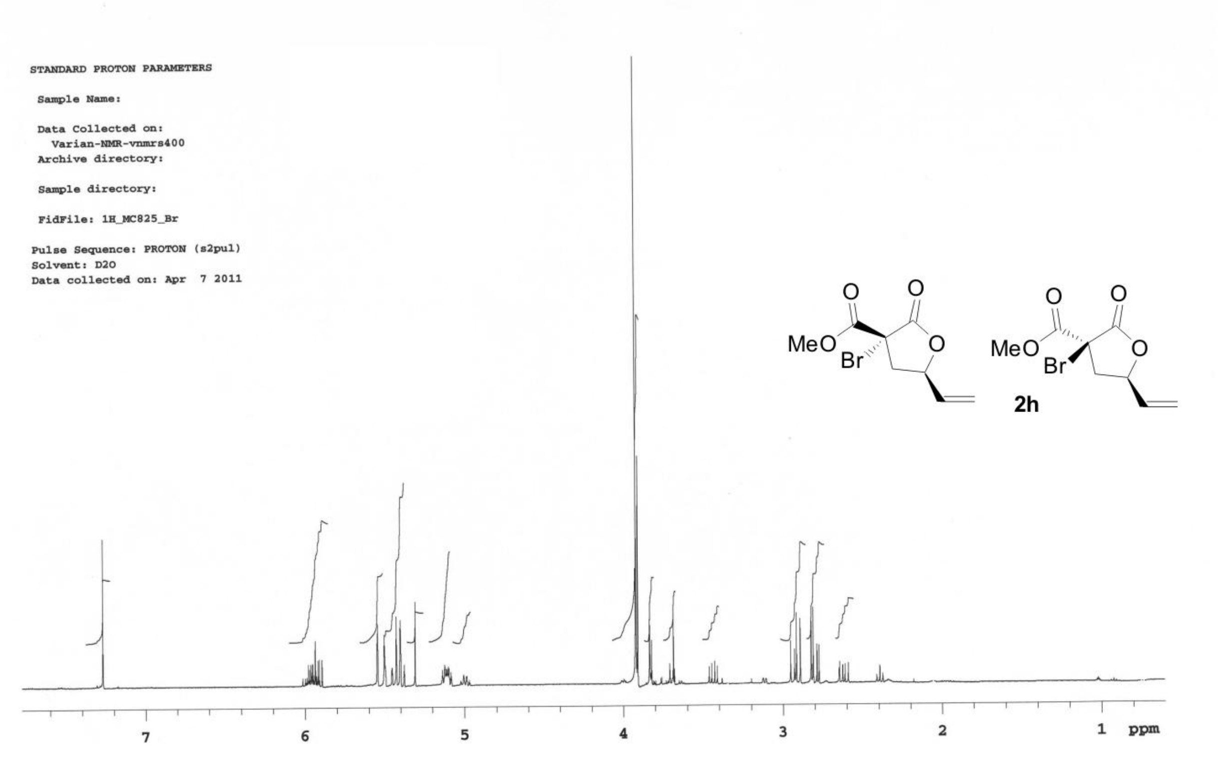

STANDARD PROTON PARAMETERS
Sample Name:
Data Collected on:
Varian-NMR-vnmrs400
Archive directory:
Sample directory:
FidFile: 1H_MC825_Br
Pulse Sequence: PROTON (s2pul)
Solvent: D2O
Data collected on: Apr 7 2011
MeO
Br
O
O
O
MeO
Br
O
O
O
2h
7
6
5
4
3
2
1
ppm

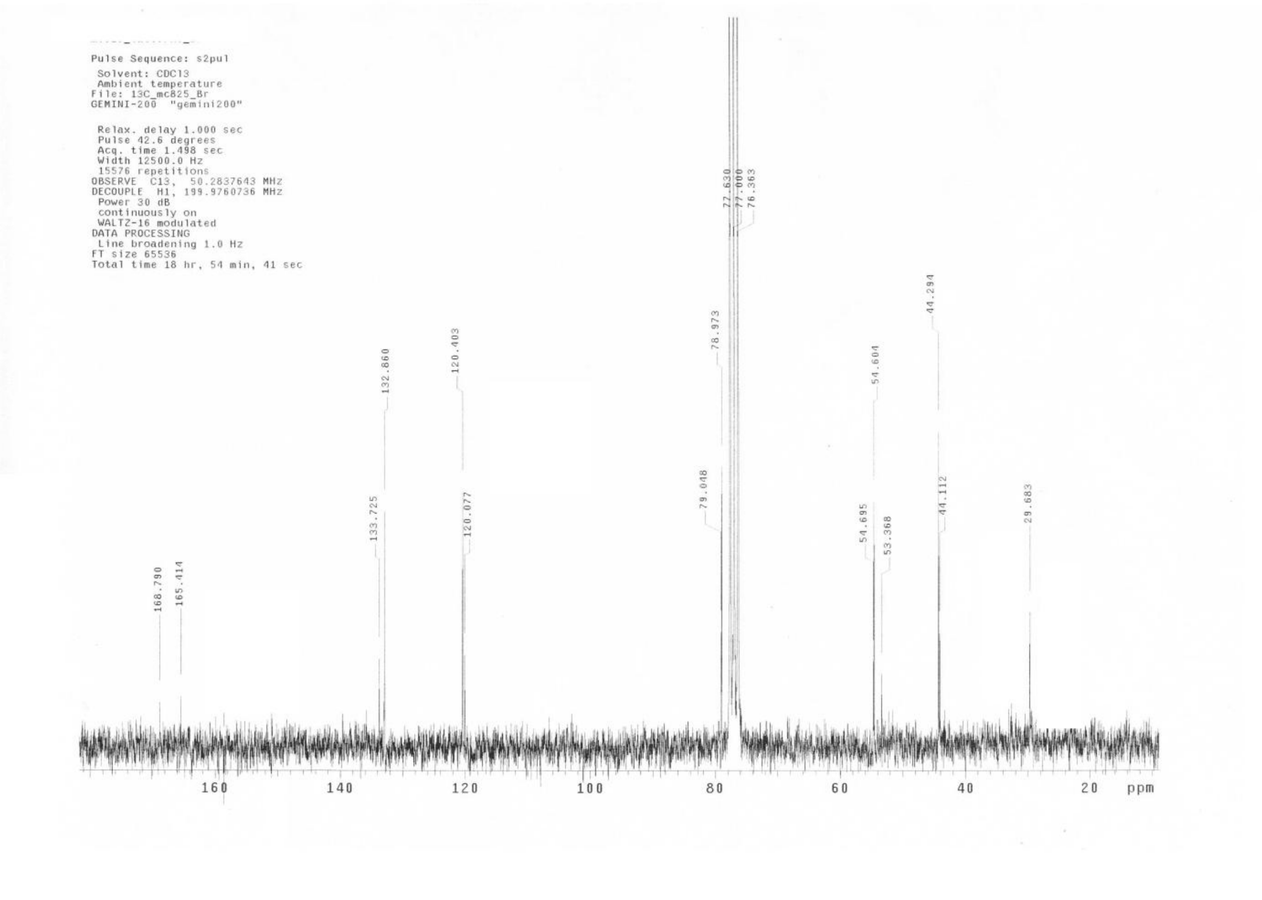

Pulse Sequence: s2pul
Solvent: CDCl3
Ambient temperature
File: 13C_mc825_Br
GEMINI-200 "gemini200"
Relax. delay 1.000 sec
Pulse 42.6 degrees
Acq. time 1.498 sec
Width 12500.0 Hz
15576 repetitions
OBSERVE C13, 50.2837643 MHz
DECOUPLE H1, 199.9760736 MHz
Power 30 dB
continuously on
WALTZ-16 modulated
DATA PROCESSING
Line broadening 1.0 Hz
FT size 65536
Total time 18 hr, 54 min, 41 sec
168.790
165.414
133.725
132.860
120.403
120.077
79.048
78.973
77.630
77.000
76.363
54.695
54.604
53.368
44.294
44.112
29.683
160
140
120
100
80
60
40
20
ppm

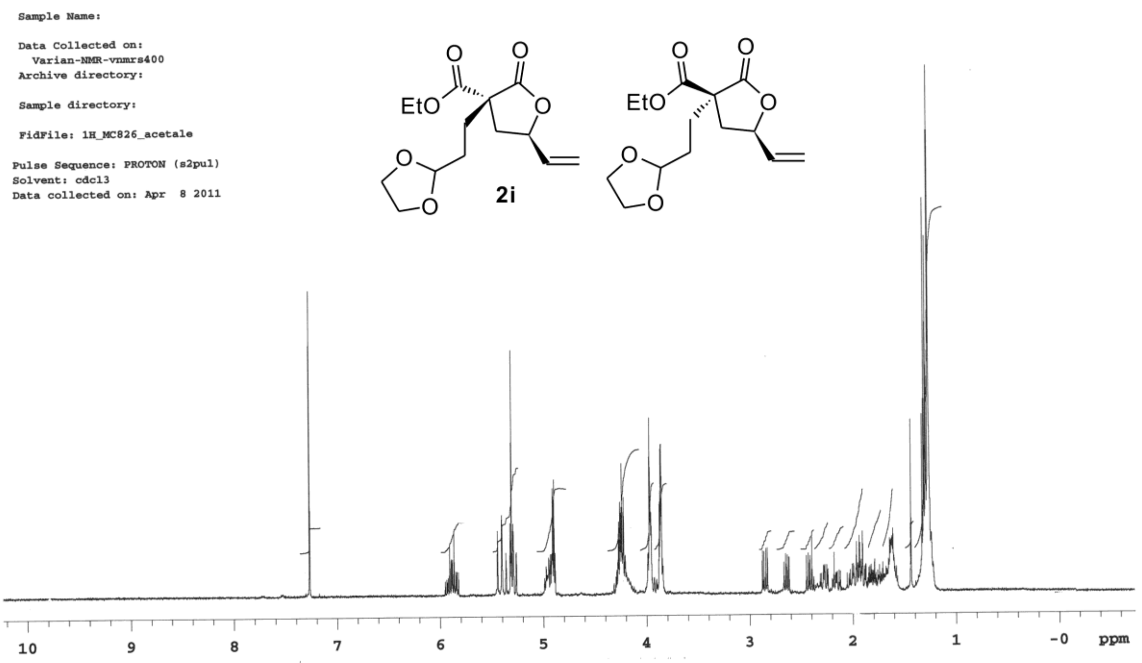
Sample Name:
Data Collected on:
Varian-NMR-vnmrs400
Archive directory:
Sample directory:
FidFile: 1H_MC826_acetale
Pulse Sequence: PROTON (s2pul)
Solvent: cdcl3
Data collected on: Apr 8 2011
EtO
2i
10 9 8 7 6 5 4 3 2 1 -0 ppm

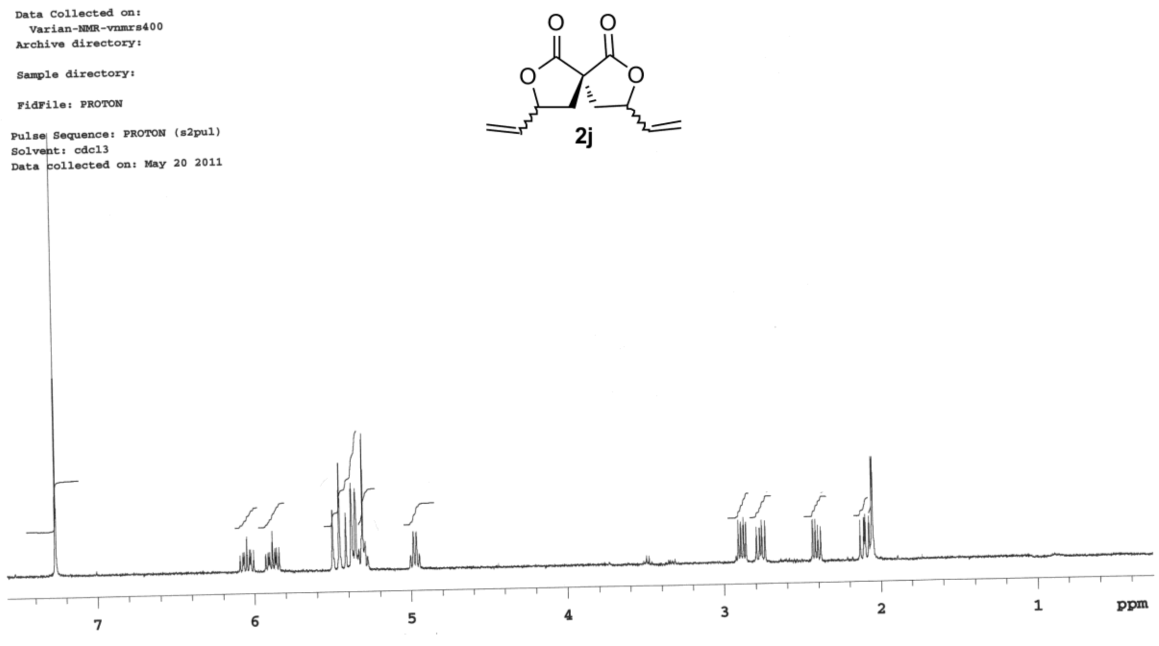
Data Collected on:
Varian-NMR-vnmrs400
Archive directory:
Sample directory:
FidFile: PROTON
Pulse Sequence: PROTON (s2pul)
Solvent: cdcl3
Data collected on: May 20 2011
2j
7 6 5 4 3 2 1 ppm

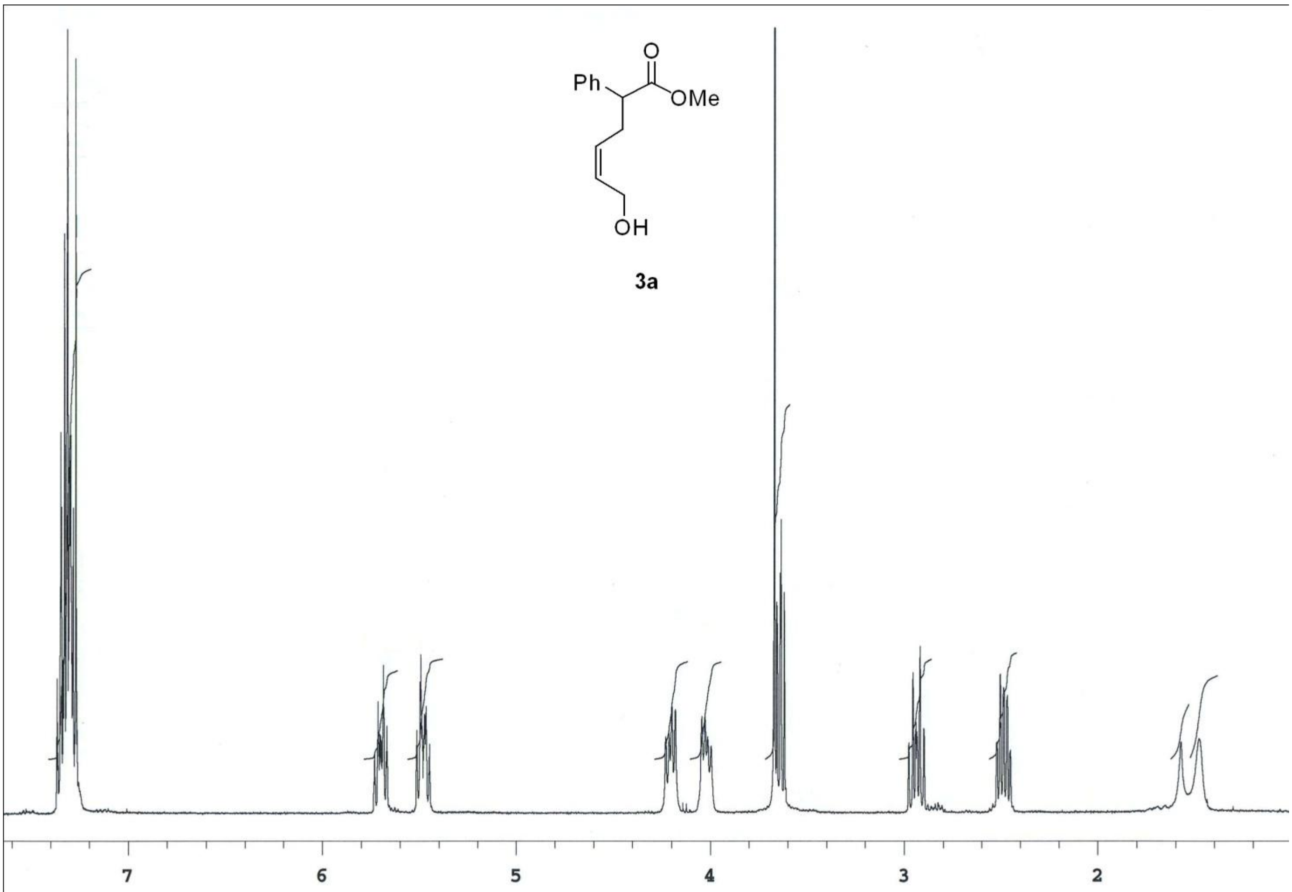
Ph
O
OMe
OH
3a
7
6
5
4
3
2

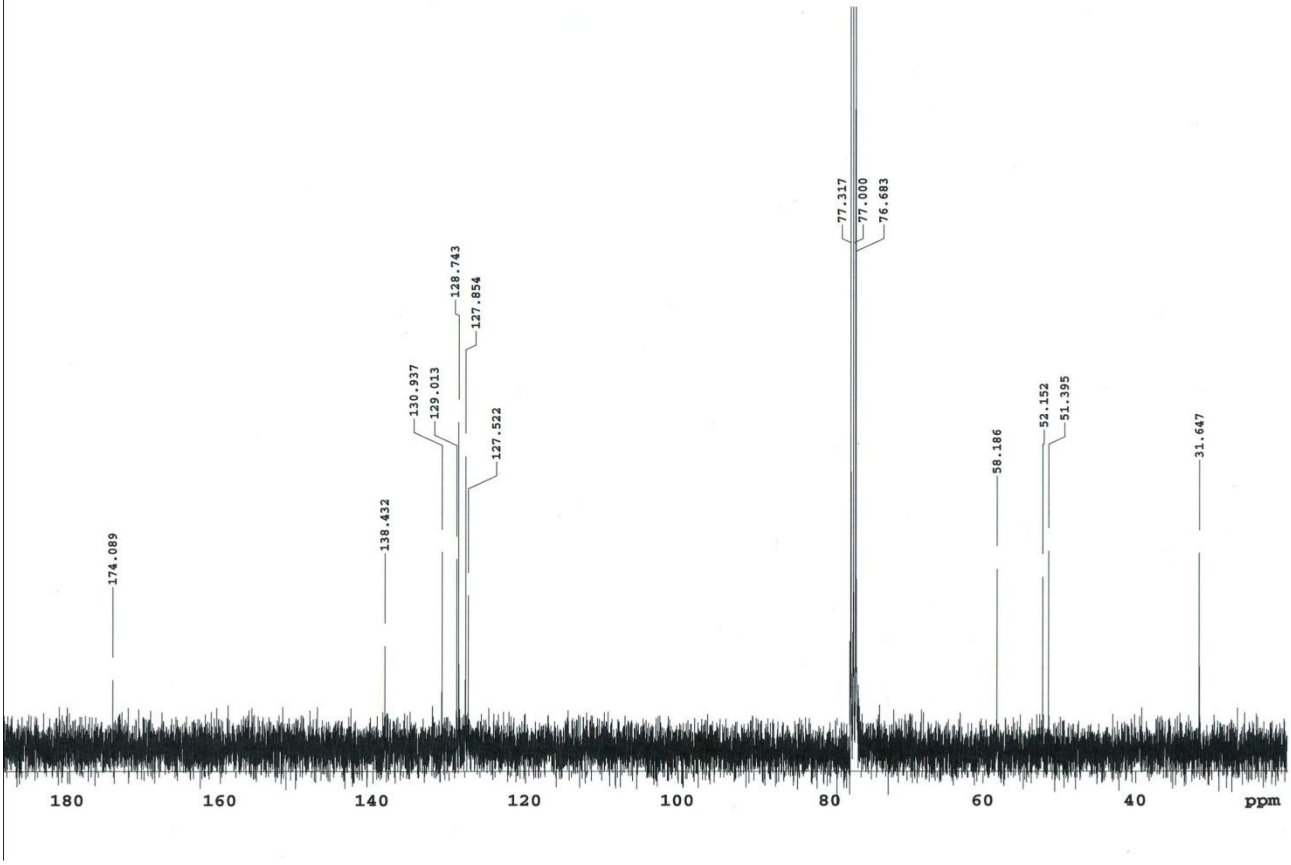
174.089
138.432
130.937
129.013
128.743
127.854
127.522
77.317
77.000
76.683
58.186
52.152
51.395
31.647
180
160
140
120
100
80
60
40
ppm

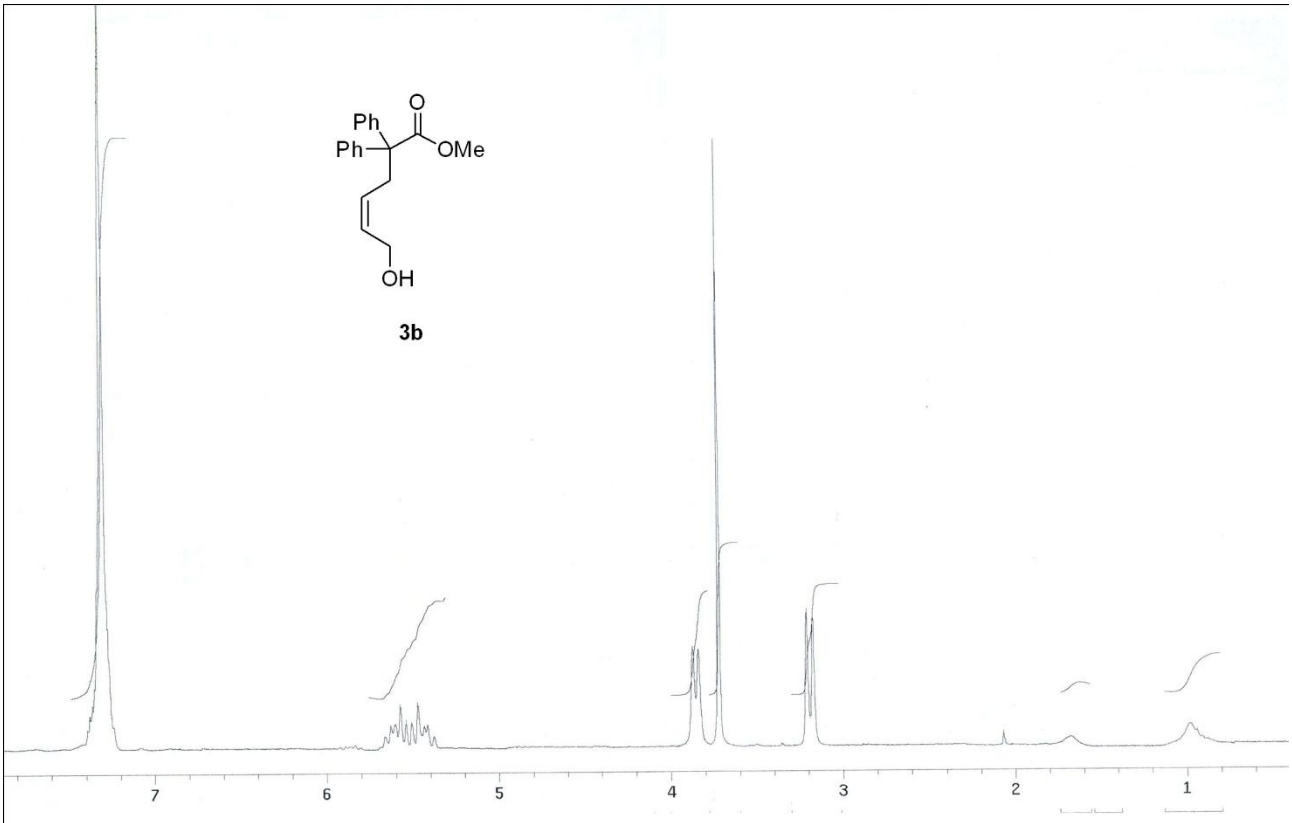
Ph
Ph
O
OMe
OH
3b
7
6
5
4
3
2
1

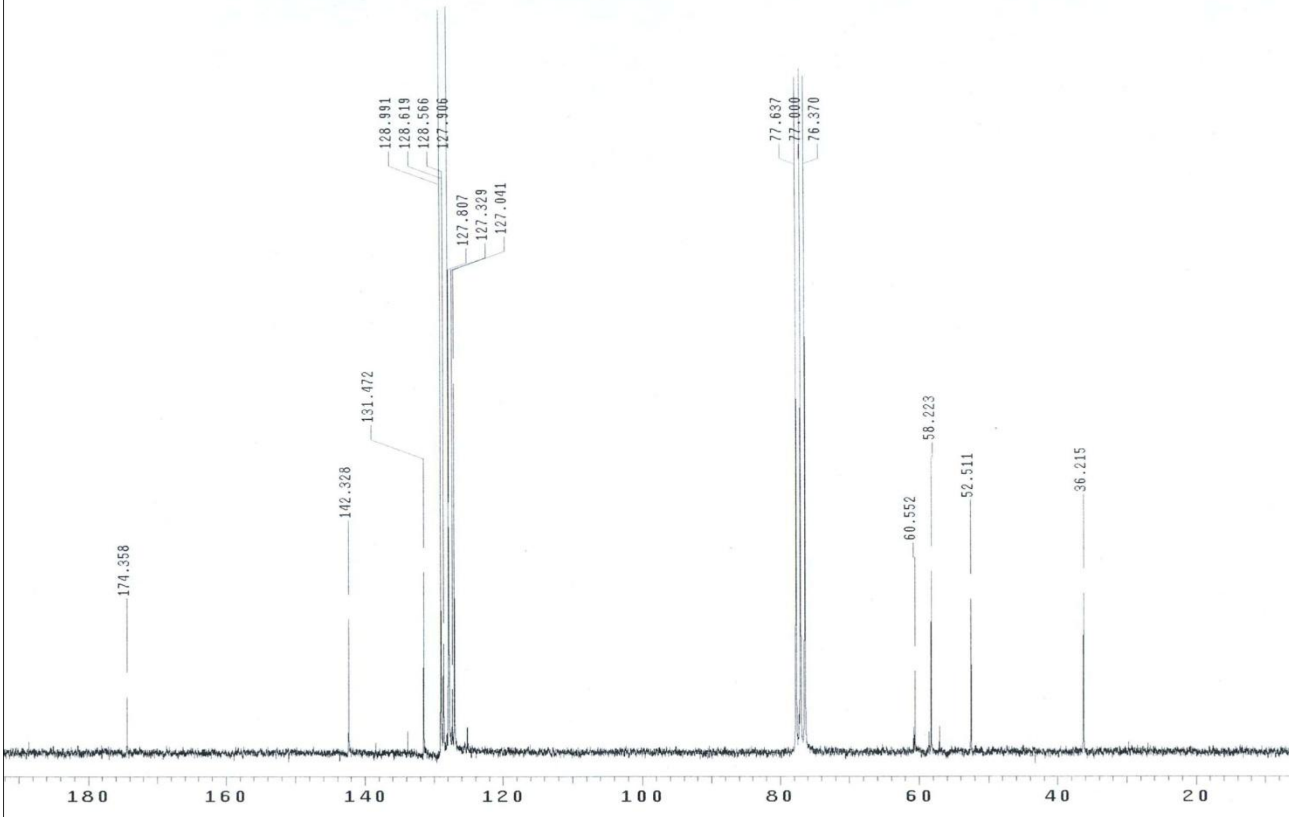
174.358
142.328
131.472
128.991
128.619
128.566
127.906
127.807
127.329
127.041
77.637
77.000
76.370
60.552
58.223
52.511
36.215
180
160
140
120
100
80
60
40
20

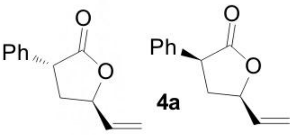

4a

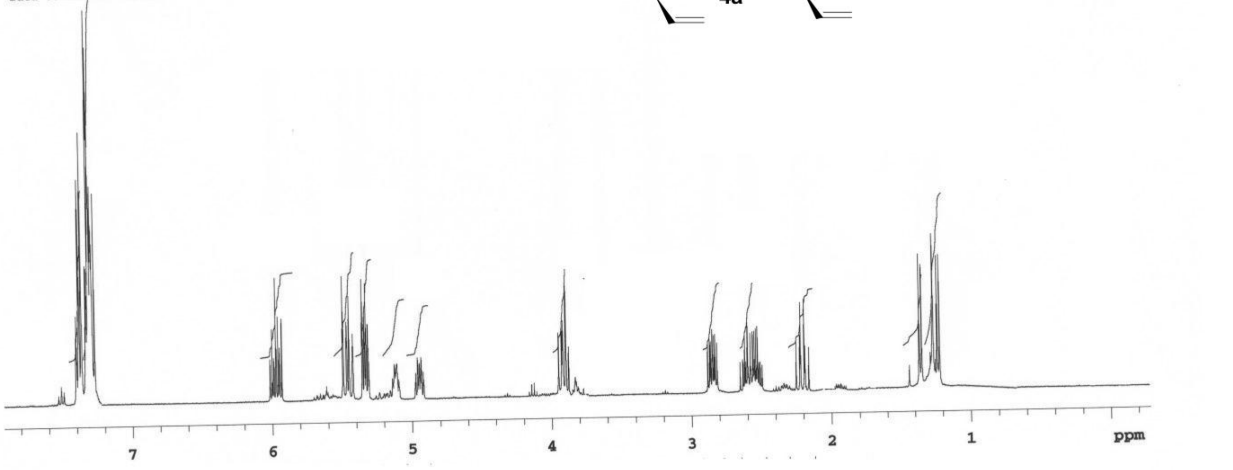
Sample Name:
mc820
Data Collected on:
Varian-NMR-vnmrs400
Archive directory:
Sample directory:
FidFile: PROTON
Pulse Sequence: PROTON (s2pul)
Solvent: cdcl3
Data collected on: Apr 5 2011
7
6
5
4
3
2
1
ppm

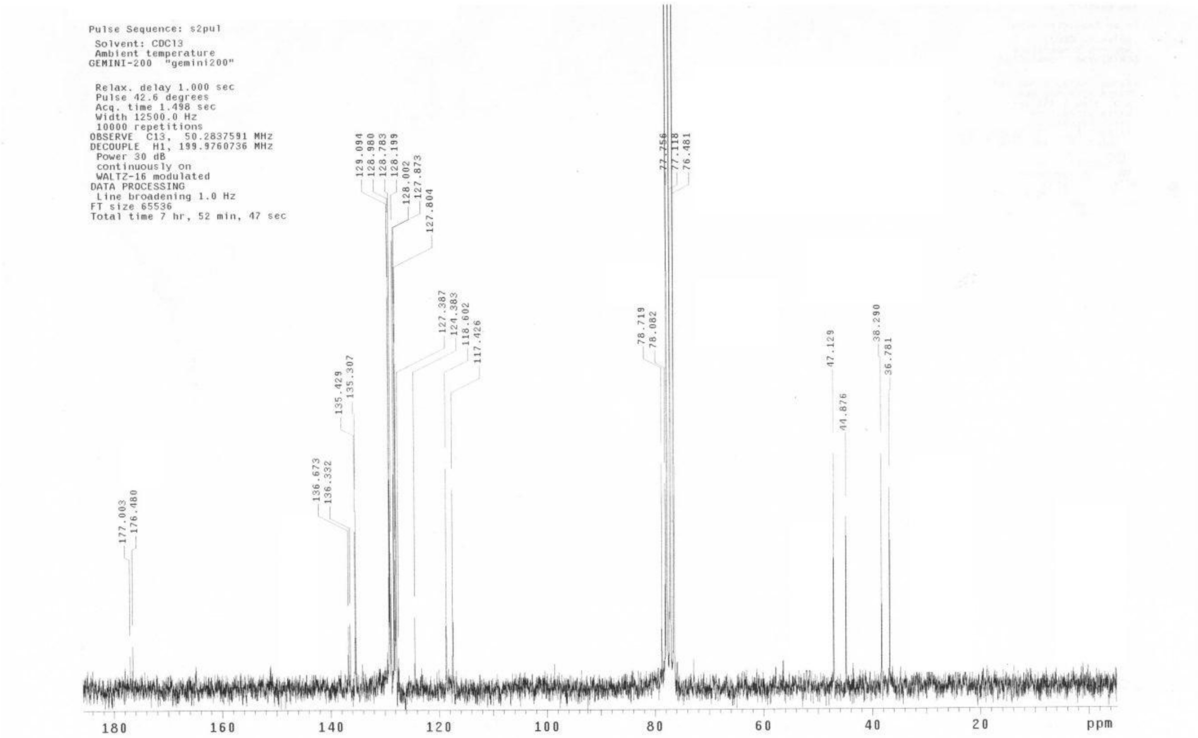
Pulse Sequence: s2pul
Solvent: CDCl3
Ambient temperature
GEMINI-200 "gemini200"
Relax. delay 1.000 sec
Pulse 42.6 degrees
Acq. time 1.498 sec
Width 12500.0 Hz
10000 repetitions
OBSERVE C13, 50.2837591 MHz
DECOUPLE H1, 199.9760736 MHz
Power 30 dB
continuously on
WALTZ-16 modulated
DATA PROCESSING
Line broadening 1.0 Hz
FT size 65536
Total time 7 hr, 52 min, 47 sec
129.094
128.980
128.783
128.199
128.002
127.873
127.804
77.756
77.118
76.481
127.387
124.383
118.602
117.426
78.719
78.082
135.429
135.307
136.673
136.332
177.003
176.480
47.129
44.876
38.290
36.781
180
160
140
120
100
80
60
40
20
ppm

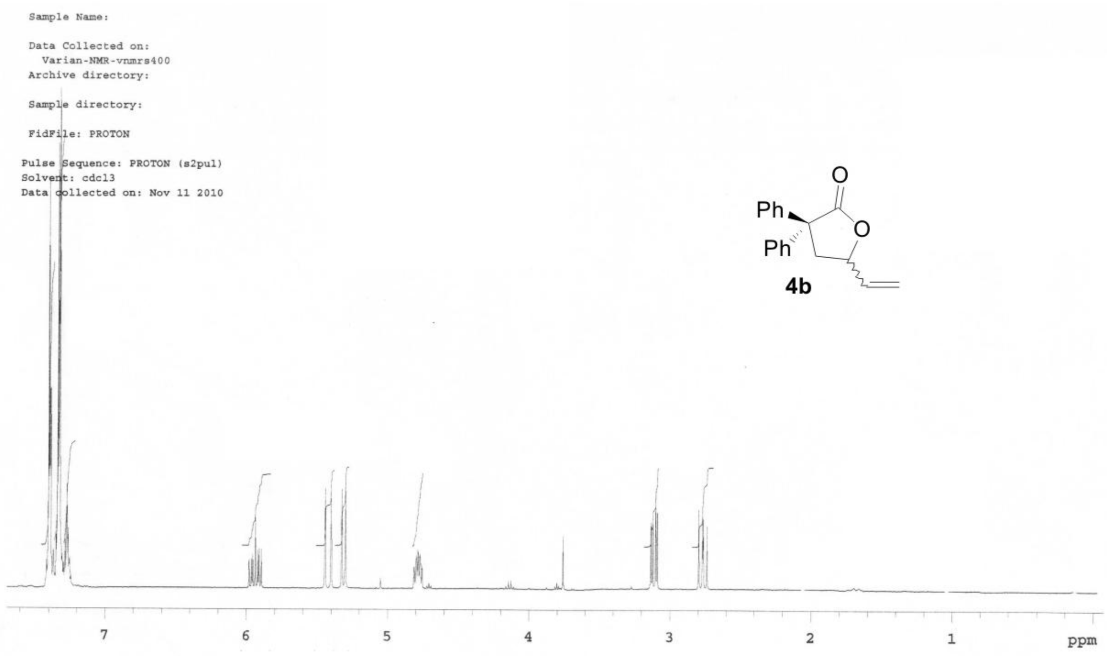
Sample Name:
Data Collected on:
Varian-NMR-vnmrs400
Archive directory:
Sample directory:
FidFile: PROTON
Pulse Sequence: PROTON (s2pul)
Solvent: cdcl3
Data collected on: Nov 11 2010
Ph
Ph
O
O
4b
7
6
5
4
3
2
1
ppm

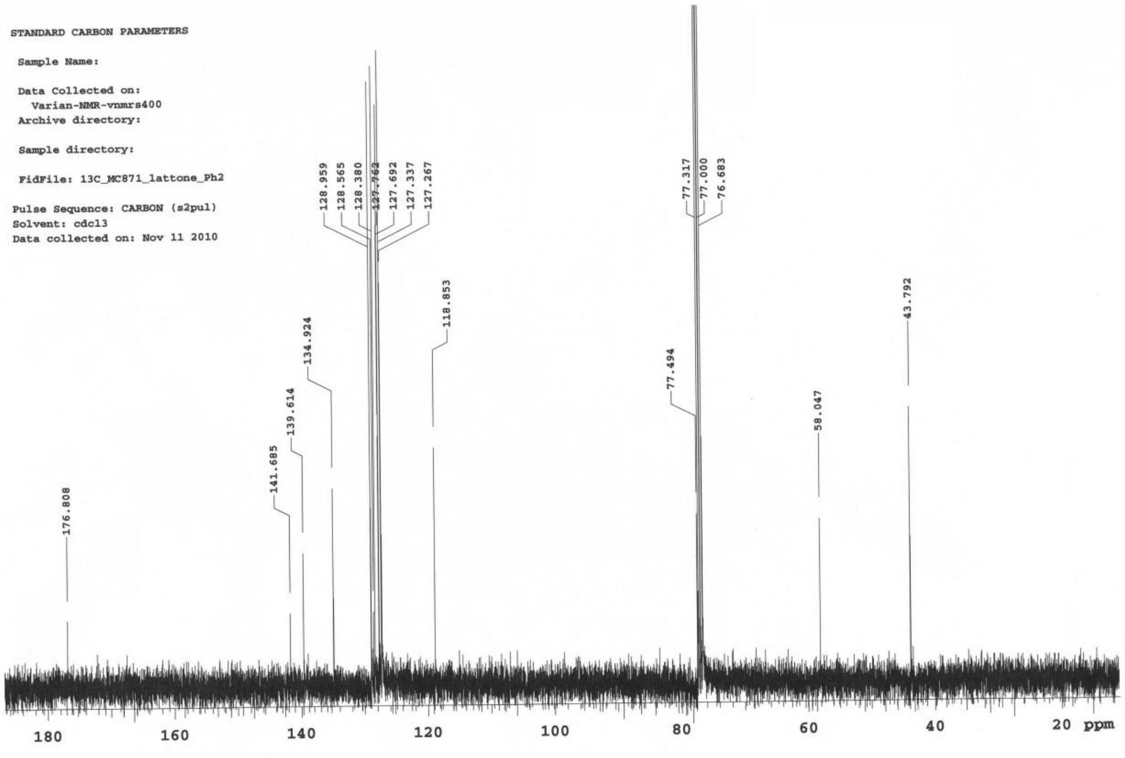
STANDARD CARBON PARAMETERS
Sample Name:
Data Collected on:
Varian-NMR-vnmrs400
Archive directory:
Sample directory:
FidFile: 13C_MC871_lattone_Ph2
Pulse Sequence: CARBON (s2pul)
Solvent: cdcl3
Data collected on: Nov 11 2010
176.808
141.685
139.614
134.924
128.959
128.565
128.380
127.762
127.692
127.337
127.267
118.853
77.494
77.317
77.000
76.683
58.047
43.792
180
160
140
120
100
80
60
40
20 ppm

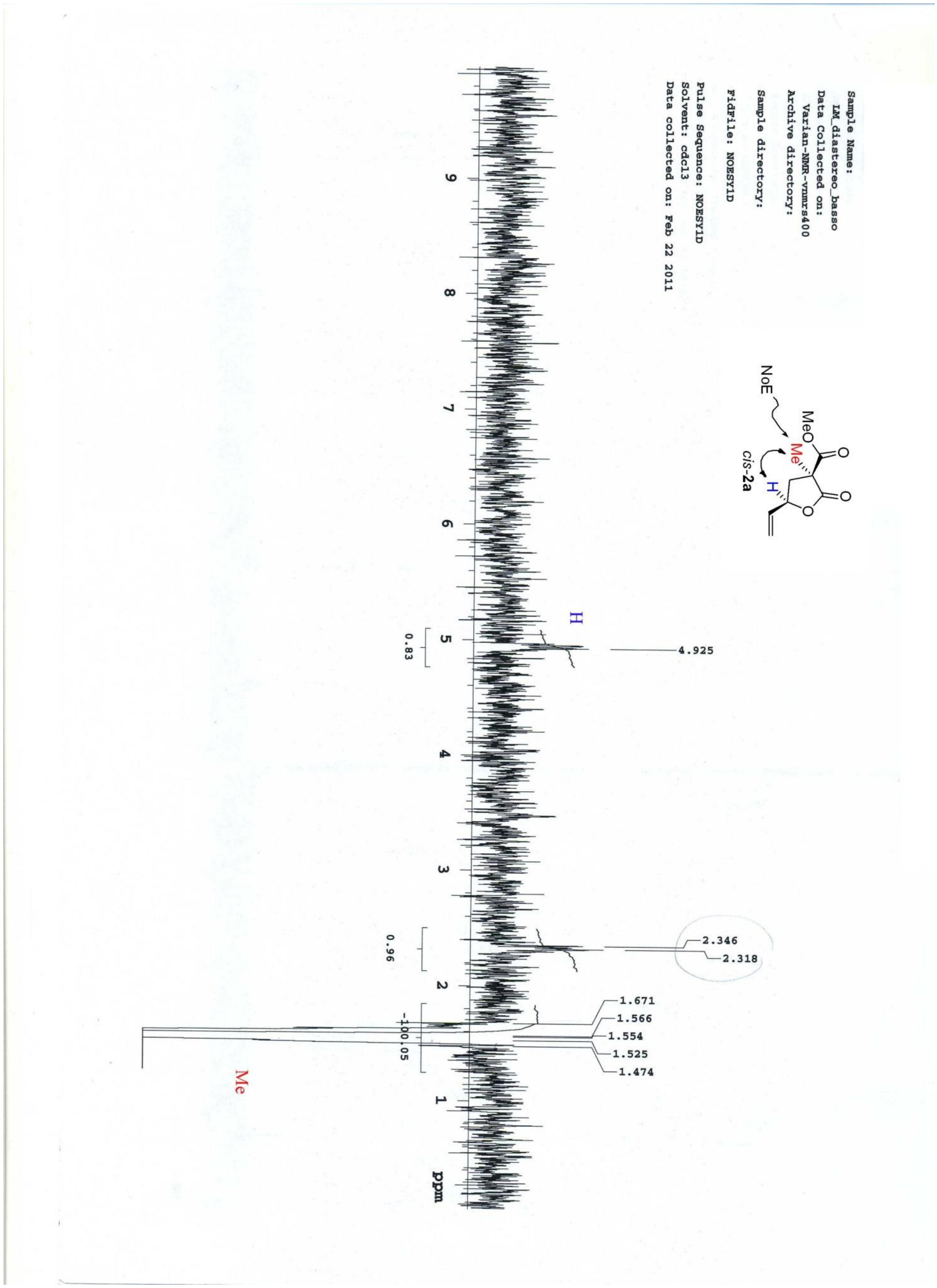
Sample Name:
LM_diastereo_basso
Data Collected on:
Varian-NMR-vnmrs400
Archive directory:
Sample directory:
FidFile: NOESY1D
Pulse Sequence: NOESY1D
Solvent: cdcl3
Data collected on: Feb 22 2011
NoE
MeO
Me
H
cis-2a
H
4.925
0.83
2.346
2.318
0.96
1.671
1.566
1.554
1.525
1.474
-100.05
Me
9
8
7
6
5
4
3
2
1
ppm
